# 2‐Phenylquinolines Exhibit Anti‐Severe Acute Respiratory Syndrome Coronavirus‐2 Activity Through the Nonstructural Protein 13 Helicase Inhibition

**DOI:** 10.1002/cmdc.202501063

**Published:** 2026-04-10

**Authors:** Giada Cernicchi, Maria Giulia Nizi, Roberta Emmolo, Leentje Persoons, Manon Laporte, Dirk Jochmans, Isabella Romeo, Giacomo Pepe, Ciro Milite, Pietro Campiglia, Jacopo Spezzini, Alma Martelli, Simone Brogi, Francesca Esposito, Tommaso Felicetti, Serena Massari, Giuseppe Manfroni, Stefano Sabatini, Gianluca Sbardella, Stefano Alcaro, Johan Neyts, Angela Corona, Enzo Tramontano, Steven De Jonghe, Oriana Tabarrini

**Affiliations:** ^1^ Department of Pharmaceutical Sciences University of Perugia Perugia Italy; ^2^ Department of Life and Environmental Sciences University of Cagliari, Cittadella Universitaria di Monserrato Cagliari Italy; ^3^ KU Leuven Department of Microbiology, Immunology and Transplantation Rega Institute for Medical Research, Molecular Genetics and Therapeutics in Virology and Oncology Research Group Leuven Belgium; ^4^ KU Leuven Department of Microbiology, Immunology and Transplantation Rega Institute for Medical Research, Virology, Antiviral Drug & Vaccine Research Group Leuven Belgium; ^5^ Department of Health Sciences University of “Magna Græcia” of Catanzaro Catanzaro Italy; ^6^ Department of Pharmacy University of Salerno Fisciano Italy; ^7^ Department of Pharmacy University of Pisa Pisa Italy; ^8^ KU Leuven Department of Microbiology, Immunology and Transplantation Rega Institute for Medical Research, Molecular, Structural and Translational Virology Research Group Leuven Belgium

**Keywords:** 2‐phenylquinolines, antivirals, human coronaviruses (HCoVs), nonstructural protein 13 (nsp13) helicase, severe acute respiratory syndrome coronavirus‐2 (SARS‐CoV‐2)

## Abstract

The Severe Acute Respiratory Syndrome Coronavirus‐2 (SARS‐CoV‐2) pandemic has highlighted the fragility of our therapeutic arsenal against human coronaviruses and the urgent need to develop new antivirals. They should exhibit broad‐spectrum activity to address future pandemics and target alternative viral proteins to mitigate resistance. We have previously identified a hit compound based on a 2‐phenylquinoline scaffold that is able to hinder SARS‐CoV‐2 replication through nonstructural protein 13 (nsp13) helicase inhibition. Here we reported a SAR study that led to identify new analogs such as 2‐(4‐butoxyphenyl)‐4‐[2‐(6,7‐dimethoxy‐3,4‐dihydroisoquinolin‐2(1H)‐yl)ethoxy]−5,7‐dimethoxyquinoline (**14**) and 4‐[2‐(6,7‐dimethoxy‐3,4‐dihydroisoquinolin‐2(1H)‐yl)ethoxy]‐2‐(4‐isopropoxyphenyl)−5,7‐dimethoxyquinoline (**15**), which exhibited a good antiviral profile (EC_50_ = 8.06 and 9.11 µM) coupled with a low micromolar inhibition of nsp13 helicase. Time‐of‐addition assays and binding analyses confirmed helicase as their primary target, while kinetic studies revealed ATP‐competitive inhibition. The butoxy derivative **14** also inhibited HCoV‐229E and HCoV‐OC43 replication, indicating broad‐spectrum potential. The safety of the compounds was validated in bronchial epithelium cells BEAS‐2B cells and H9c2 cardiac cells, where they did not affect cell viability or reactive oxygen species (ROS) production. Finally, preliminary ADME studies on **15** showed a positive profile in terms of membrane permeability and metabolic stability in plasma and human liver microsomes. This SAR study, along with mechanistic exploration, paves the way for further optimization of 2‐phenylquinoline‐based compounds.

## Graphical abstract

A SAR study led to the identification of new 2‐phenylquinoline‐based derivatives that exhibit anti‐Severe Acute Respiratory Syndrome Coronavirus‐2 (SARS‐CoV‐2) activity by acting as ATP‐competitive inhibitors of nonstructural protein 13 (nsp13) helicase. They showed a broad spectrum of anti‐CoV activity, have favorable PK properties and were nontoxic in various cell lines. In silico studies provided insights into the binding profiles of the ligands, paving the way for future optimization.

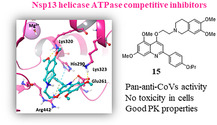

## 1 Introduction

Coronaviruses (CoVs) are a family of enveloped positive‐sense single‐stranded RNA viruses able to infect both humans and animals, mainly resulting in respiratory and enteric diseases.

While four out of the seven known human CoVs (HCoVs) [1] are typically responsible for common cold, in the past decades, humanity also experienced three viral outbreaks caused by the highly pathogenic HCoVs: severe acute respiratory coronavirus (SARS‐CoV, 2003) [2], Middle East respiratory syndrome coronavirus (MERS‐CoV, 2012) [3], and SARS‐CoV‐2 (from 2019 to 2023) [4], the latter evolved into an unprecedented disruptive pandemic event with a severe socioeconomic impact. The coronavirus disease (COVID‐19) pandemic has clearly highlighted the lack of effective therapeutic countermeasures. Indeed, until late 2020, no approved drugs or vaccines were available for the treatment of HCoV infections, thereby facilitating viral spread and increasing mortality rates [5].

In response to the COVID‐19 crisis, a global collaborative effort involving public health agencies, biotech start‐ups, academic institutions, and major pharmaceutical companies such as Johnson & Johnson and Pfizer enabled the rapid development of vaccines. Since December 2020, these vaccines have been deployed in mass immunization campaigns, significantly reducing severe cases, hospitalizations, and mortality worldwide. Parallel to vaccine development, the urgent need for antiviral therapeutics against SARS‐CoV‐2 accelerated the approval of three antiviral drugs, the RNA‐dependent RNA polymerase (RdRp) inhibitors, molnupiravir (Lagevrio) [6] and remdesivir (Veklury) [7], and the main protease (Mpro) inhibitor nirmatrelvir coadministered with ritonavir (Paxlovid) [8]. This rapid progress was largely driven by drug repurposing strategies.

Despite these initial successes, a number of drawbacks are associated with the currently available armamentarium against SARS‐CoV‐2 [9]. In fact, after only 1 year of clinical use, molnupiravir was withdrawn from the market due to its lack of clinical benefit. Because of its need for intravenous administration, use of remdesivir is limited to hospital settings. In addition, drug‐resistant variants have emerged rapidly for all approved drugs [10, 11]. The risk of future spillover events involving highly pathogenic HCoVs remains imminent, emphasizing the urgent need for novel antivirals with innovative mechanisms and broad‐spectrum activity. The World Health Organization (WHO) has reinforced this priority in its 2024 update of the “Blueprint list of priority pathogens”‐ [12].

**FIGURE 1:**
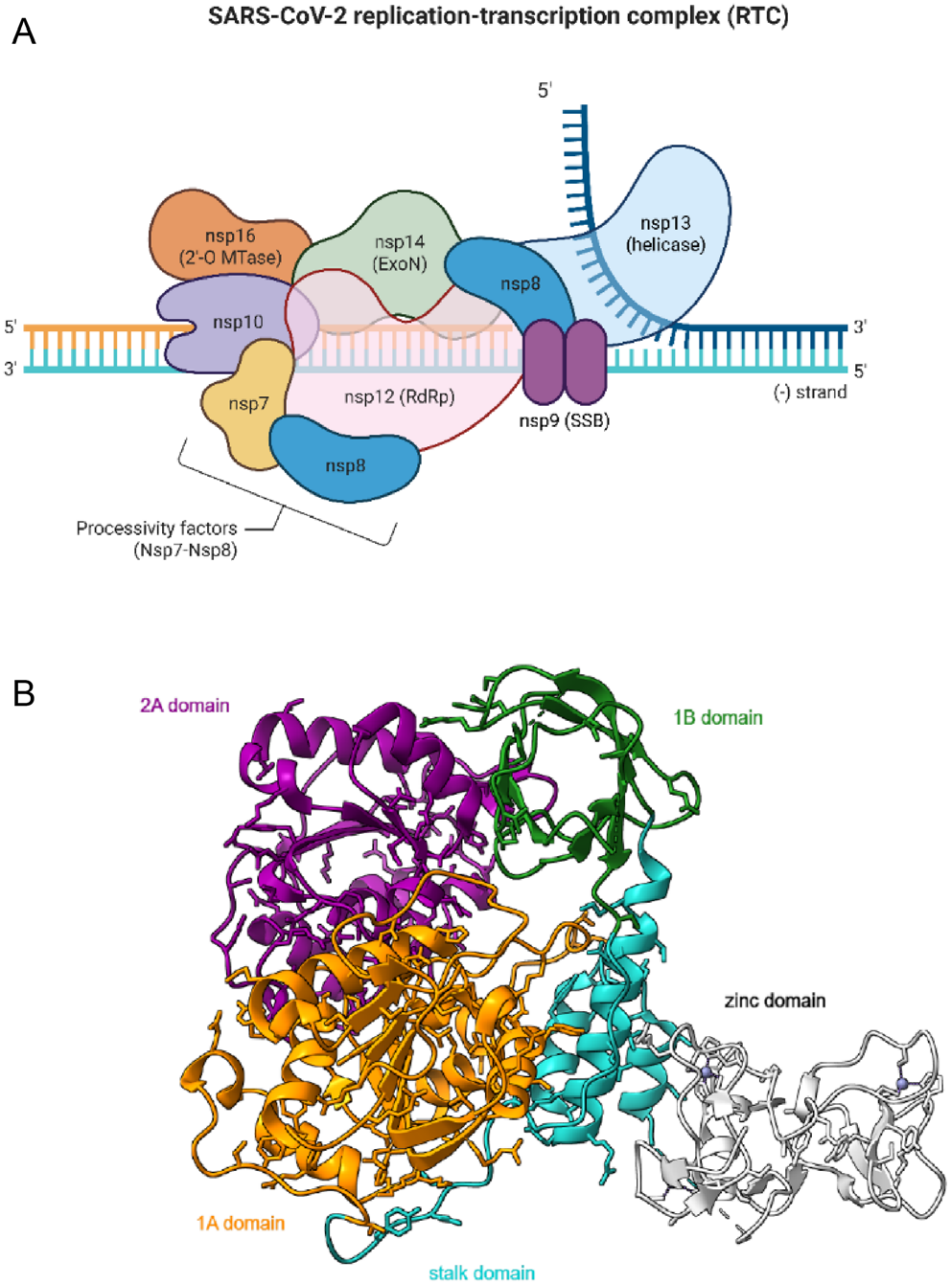
(A) Architecture of the SARS‐CoV‐2 replication transcription complex, adapted from Hartenian et al. [14] using BioRender and (B) structure overview of nsp13 (PDB ID: 5RMC; picture generated with ChimeraX) [15] with domains labeled and colored individually.

The SARS‐CoV‐2 nonstructural protein 13 (nsp13) helicase has been relatively unexplored as an antiviral drug target [13]. It is a key player in the viral replication–transcription complex (RTC), along with the RdRp nsp12, nsp7, and nsp8 proteins (Figure 1A). It belongs to the 1B helicase superfamily and catalyzes the nucleoside triphosphates (NTP)‐dependent unwinding of double‐stranded DNA or RNA in the 5′‐3′ direction. From a structural point of view, SARS‐CoV‐2 nsp13 architecture is shared with that of other nidovirus helicases and it is composed of five domains: two RecA‐like domains (1A and 2A) responsible for nucleotide binding and hydrolysis, an *N*‐terminal zinc binding domain (ZBD) that coordinates two Zn^2+^ ions, a helical “stalk” domain and a beta‐barrel 1B domain (Figure 1B) [16].

The nsp13 helicase is one of the most conserved nsps, with more than 99% conservation among the various SARS‐CoV‐2 variants and only a single amino acid difference between SARS‐CoV‐1 and SARS‐CoV‐2.

**FIGURE 2:**
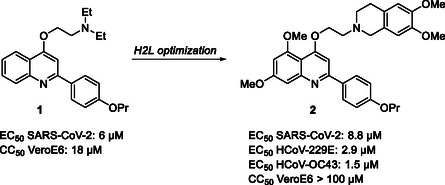
Chemical structures and antiviral profiles of compounds **1** and **2**.

To date, the number of antivirally active SARS‐CoV‐2 nsp13 inhibitors is limited [17, 18, 19, 20, 21, 22]. We previously reported the screening of an in‐house library of structurally diverse small molecules that yielded the 2‐phenylquinoline (2‐PhQ) analog **1** with promising anti‐SARS‐CoV‐2 activity (Figure 2) [17]. A first hit‐to‐lead (H2L) optimization campaign, entailing the synthesis and antiviral evaluation of approximately 40 analogs, confirmed the suitability of the 2‐PhQ scaffold to afford compounds with low µM anti‐SARS‐CoV‐2 activity, whereas lacking cytotoxicity. These compounds also inhibited HCoV‐229E and HCoV‐OC43, suggesting a pan‐anticoronavirus profile. Classical targets, such as RdRp and Mpro, were excluded as the mode of action of the 2‐PhQs. Given the structural similarity of 2‐PhQs to chloroquine, autophagy inhibition in VeroE6 cells was evaluated, but found to be weak and insufficient to explain the antiviral effect. Instead, mechanistic studies revealed that one of the most potent compounds, analog **2** (Figure 2), inhibited the nsp13 helicase unwinding activity with an IC_50_ value of 0.42 µM.

In this study, starting from the hit compound **2**, a series of twenty‐seven novel analogs (compounds **3–29**) was designed and synthesized with the primary aim to better define the SAR around the 2‐PhQ scaffold and validate the nsp13 as a target. Thus, all the compounds were evaluated for anti‐SARS‐CoV‐2 activity and cytotoxicity in Vero cells, as well as for nsp13 inhibition (Table 1). Among the new compounds, **14** and **15** emerged as the most promising candidates, thereby undergoing comprehensive biological characterization. To evaluate the potential broad‐spectrum anti‐HCoV profile, their efficacy was further tested against HCoV‐229E and HCoV‐OC43. Time‐of‐addition (TOA) experiments and binding mode analyses, along with kinetic experiments, were performed to further investigate the mode of action. Finally, their cytotoxicity on bronchial epithelial cells and cardiac myoblasts was tested, and a preliminary ADME profiling was performed to establish their potential for future development.

## 2 Results and Discussion

### 2.1 Chemistry

**SCHEME 1:**
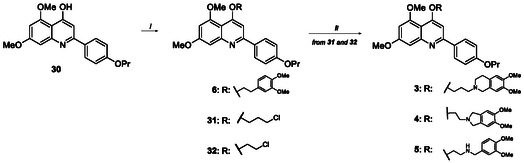
Reagents and conditions: (*i*) 4‐(2‐chloroethyl)−1,2‐dimethoxybenzene, 1‐bromo‐3‐chloropropane or 1‐bromo‐2‐chloroethane, K_2_CO_3_, dry DMF, 80°C, 2–10 h, 33%–50% and (*ii*) 6,7‐dimethoxy‐1,2,3,4‐tetrahydroisoquinoline hydrochloride or 5,6‐dimethoxyisoindoline or 3,4‐dimethoxybenzylamine, K_2_CO_3_, dry DMF, 80°C, 5–8 h, 10%–72%.

The synthesis of compounds **3–6** is reported in Scheme 1 and entailed the functionalization of the dimethoxy‐2‐(4‐propoxyphenyl)quinolin‐4‐ol scaffold **30** [23]. Alkylation with 4‐(2‐chloroethyl)−1,2‐dimethoxybenzene, using K_2_CO_3_ as a base in dry DMF, afforded the target compound **6**, while the reaction with 1‐bromo‐3‐chloropropane or 1‐bromo‐2‐chloroethane, under the same reaction conditions, gave the intermediates **31** and **32**, respectively. The successive nucleophilic substitution reaction of **31** with 6,7‐dimethoxy‐1,2,3,4‐tetrahydroisoquinoline hydrochloride gave compound **3**, while the reaction of **32** with either 5,6‐dimethoxyisoindoline or 3,4‐dimethoxybenzylamine furnished the target compounds **4** and **5**, respectively.

**SCHEME 2:**
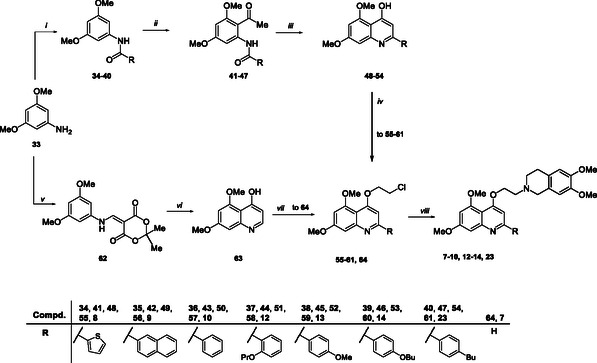
Reagents and conditions: (*i*) properly substituted benzoyl chloride, Et_3_N, dry THF, 0°C → rt, 12 h, 50%–98%; (*ii*) SnCl_4_, acetyl chloride, dry CH_2_Cl_2_, 0°C → rt, 24 h, 20%–78%; (*iii*) *t*‐BuOK, *t*‐BuOH, r.t./50°C, 12 h, 26%–95%; (*i* *v*) 1‐bromo‐2‐chloroethane, K_2_CO_3_, 80°C, 4–24 h, 61%–85%; (*v*) Meldrum's acid, trimethyl orthoformate, DMF, reflux, 2 h, 85%; (*vi*) Dowtherm A 240°C, 1.5 h, 40%; (*vii*) PPh_3_, DIAD, dry THF, 2‐chloroethanol, 0°C to rt, 48 h, 38%; and (*viii*) 6,7‐dimethoxy‐1,2,3,4‐tetrahydroisoquinoline hydrochloride, K_2_CO_3_, dry DMF, 80°C, 3–12 h, 25%–63%.

The synthesis of the 5,7‐dimethoxy quinoline derivatives **7**‐**10**, **12**‐**14**, and **23** is reported in Scheme 2. Intermediates **34–40**, that were obtained by coupling of properly functionalized benzoyl chlorides with 3,5‐dimethoxyaniline **33**, were subjected to a Friedel–Crafts acylation with acetyl chloride and SnCl_4_ in dry CH_2_Cl_2_ to give compounds **41–47**. The subsequent cyclization in the presence of *t*‐BuOK in *t*‐BuOH afforded the quinolines **48–54**. Alkylation of the hydroxyl group at C‐4 by reaction with 1‐bromo‐2‐chloroethane furnished intermediates **55–61**, which were subjected to a nucleophilic substitution with 6,7‐dimethoxy‐1,2,3,4‐tetrahydroisoquinoline hydrochloride to obtain target compounds **8**‐**10**, **12**‐**14** and **23**.

The synthesis of derivative **7** also started from the commercially available 3,5‐dimethoxyaniline **33**. Following the strategy reported by Peng et al. [24], trimethyl orthoformate was refluxed with Meldrum's acid, followed by the addition of 3,5‐dimethoxyaniline **33** in the presence of DMF to give intermediate **62**, which was then cyclized using preheated Dowtherm A at 240°C, yielding the 5,7‐dimethoxy quinoline core **63**. The subsequent Mitsunobu reaction with 2‐chloroethanol afforded chloroethyl derivative **64** which was substituted with 6,7‐dimethoxy‐1,2,3,4‐tetrahydroisoquinoline hydrochloride to obtain target compound **7**.

**SCHEME 3:**
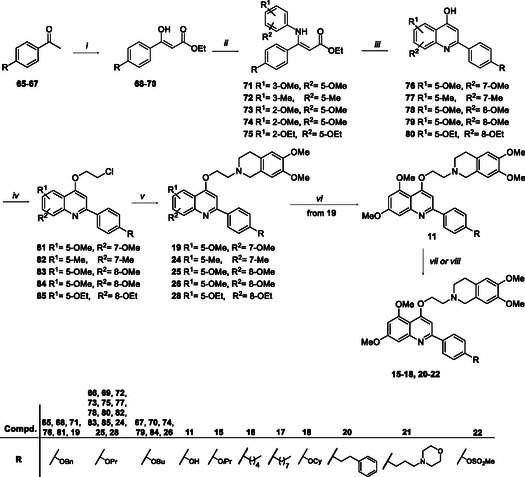
Reagents and conditions: (*i*) 60% NaH, (EtO)_2_CO, rt, → 70°C, 1–24 h, 76%–95%; (*ii*) properly substituted aniline, *p*‐TsOH, dry benzene, reflux, 24 h, 22%–58%; (*iii*) Dowtherm A, 240°C, 1.5–4 h, 34%–80%; (*iv*) 1‐bromo‐2‐chloroethane, K_2_CO_3_, dry DMF, 80°C, 4–24 h, 30%–98%; (*v*) 6,7‐dimethoxy‐1,2,3,4‐tetrahydroisoquinoline hydrochloride, K_2_CO_3_, dry DMF, 80°C, 5–12 h, 21%–50%; (*vi*) Pd/C, ammonium formate, MeOH, rt, 2 h, 60%, (*vii*) proper halogen derivatives, K_2_CO_3_ dry DMF, 80°C, 3–12 h, 20%–63%; and (*viii*) pentyl 4‐methylbenzenesulfonate or octyl 4‐methylbenzenesulfonate or cyclohexyl *p*‐toluenesulfonate, Cs_2_CO_3_, dry DMF, 80°C, 1–12 h, 29%–41%.

For the synthesis of 5,8‐dimethoxy, 5,8‐diethoxy‐, 5,7‐dimethoxy and 5,7‐dimethyl‐quinoline derivatives **11**, **15–22**, **24**‐**26**, and **28**, the key intermediates **76–** **80** were prepared as shown in Scheme 3. The commercially available acetophenones **65–** **67** were reacted with diethylcarbonate and NaH to obtain acrylates **68–** **70** in good yields, which were reacted with properly substituted anilines in the presence of a catalytic amount of *p*‐TsOH and using dry benzene as a solvent affording aminoacrylates **71–** **75**. Intermediates **71–** **75** were, in turn, cyclized in preheated Dowtherm A at 240°C, yielding the quinolines **76–** **80** in 34 to 80% yields. The following reaction of **76–** **80** with 1‐chloro‐2‐bromoethane furnished intermediates **81–85** that were reacted with 6,7‐dimethoxy‐1,2,3,4‐tetrahydroisoquinoline hydrochloride using K_2_CO_3_ as base in dry DMF, furnishing target compounds **19**, **24**‐**26** and **28**. Compound **19** was then debenzylated using Pd/C and ammonium formate in MeOH, affording compound **11**. Reaction of **11** with properly selected alkyl halides gave the target compounds **15** and **20–** **22.** Moreover, reaction of **11** with pentyl 4‐methylbenzenesulfonate, octyl 4‐methylbenzenesulfonate and cyclohexyl *p*‐toluenesulfonate yielded target compounds **16**‐**18**, respectively.

**SCHEME 4:**
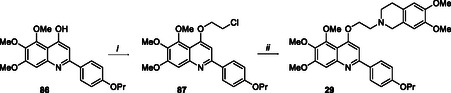
Reagents and conditions: (*i*) 1‐bromo‐2‐chloroethane, K_2_CO_3_, dry DMF, 80°C, 3 h, 60% and (*ii*) 6,7‐dimethoxy‐1,2,3,4‐tetrahydroisoquinoline hydrochloride, K_2_CO_3_, dry DMF, 80°C, 12 h, 60%.

The synthesis of the trimethoxy derivative **29** is depicted in Scheme 4. Intermediate **86**, synthesized as previously reported [17], was alkylated with 1‐bromo‐2‐chloroethane to give intermediate **87** in moderate yield. The subsequent substitution with 6,7‐dimethoxy‐1,2,3,4‐tetrahydroisoquinoline hydrochloride afforded the target compound **29**.

### 2.2 Design and Evaluation of Analogs of Hit Compound 2 for SARS‐CoV‐2 and nsp13 Inhibition (Table 1)

Compound **2** was previously identified as a promising anti‐SARS‐CoV‐2 agent acting via nsp13 helicase inhibition [17]. SAR exploration of compound **2** started with elongation of the linker between the dimethoxytetrahydroisoquinoline (DMTHI) and quinoline moiety from 2 to 3 carbon atoms, furnishing derivative **3**. In the other compounds, the DMTHI group was modified by reducing the size, as in the isoindoline derivative **4**, opening or deleting one of the rings, as in **5** and **6**, respectively. Once it was confirmed that the DMTHI moiety was crucial for conferring potent nsp13 inhibition, it was kept constant in the subsequent series of derivatives, in which the C‐2 position was extensively explored, preparing compounds **7–23**. Minor modifications concerned the benzene substituents on the quinoline scaffold, as in compounds **24–29**.

**TABLE 1:** Chemical structures and biological profiles of 2‐PhQs: anti‐SARS‐CoV‐2 activity, cytotoxicity, and helicase inhibition.

Compd	R/Ar		**SARS‐CoV‐2** **EC**_**50**_ **, µM** a		**VeroE6 cells** **CC**_**50**_ **, µM** b	**ATPase** **IC**_**50**_ **, µM** c	**Unwinding IC**_**50**_ **, µM** d	
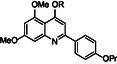								
**2**	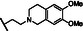		8.83 ± 0.58^e^		>100^e^	18.8 ± 3.2	21.2 ± 1.8	
**3**	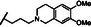		>4.52		4.52 ± 0.53	24.67 ± 1.33	>30 (55%)^f^	
**4**	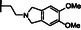		7.18 ± 1.70		23.96 ± 1.12	>30 (67%)	28.2 ± 2.4	
**5**	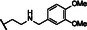		>6.86		6.86 ± 0.27	>30	>30	
**6**	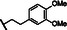		>100		>100	>30 (87%)	>30 (100%)	
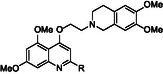								
**7**	H			>28.30	28.30 ± 3.04	>30(100%)	>30 (100%)	
**8**	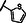			>11.74	>100	22.81 ± 3.80	23.78 ± 1.02	
**9**	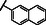			9.70 ± 0.38	14.74 ± 3.88	8.99 ± 1.94	22.2 ± 3.6	
**10**	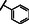			>100	>100	>30 (90%)	22.7 ± 6.6	
**11**	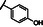			>4.97	4.97 ± 0.11	25.73 ± 2.19	>30 (46%)	
**12**	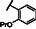			>100	>100	19.47 ± 1.43	20.08 ± 0.96	
**13**	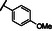			>8.98	8.98 ± 0.30	18.83 ± 1.21	>30 (44%)	
**14**	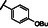			8.06 ± 0.38	>80.5	11 ± 3.19	10.39 ± 0.6	
**15**	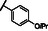			9.11 ± 0.61	>100	19.36 ± 0.43	23.98 ± 0.12	
**16**	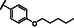			>20.13	>85.75	>30 (96%)	23.9 ± 2.6	
**17**	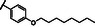			>78.47	78.47 ± 5.28	>30 (100%)	22.2 ± 0.8	
**18**	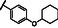			>90.02	>90.02	7.38 ± 0.54	13.79 ± 1.03	
**19**	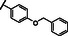			>98.05	98.05 ± 0.80	5.15 ± 0.91	19.4 ± 2.9	
**20**	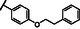			>100	>100	14.02 ± 0.18	11.6 ± 2.32	
**21**	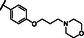			>20.69	20.69 ± 10.09	>30 100%	>30 (100%)	
**22**	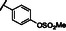			>12.99	12.99 ± 2.33	15.68 ± 1.72	29.05 ± 0.14	
**23**	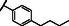			12.72 ± 0.09	>100	>30 (74%)	22.4 ± 4.3	
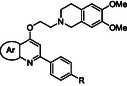								
**24**	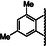	R = OPr		>100	>100	24.27 ± 3.41	>30 (55%)	
**25**	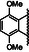	R = OPr		6.82 ± 0.31	>100	15.4 ± 1.4	>30 (77%)	
**26**	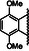	R =OBu		26.16 ± 16.07	>100	9.31 ± 0.14	29.65 ± 1.11	
**27** [23]	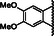	R OPr		>24.46	>100	10.13 ± 2.7	>30 (34%)	
**28**	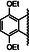	R = OPr		>100	>100	24.1 ± 6.7	15.5 ± 2.9	
**29**	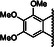	R = OPr		>100	>100	23.39 ± 5.02	17.43 ± 2.75	
**GS‐441524**				0.7 ± 0.07	72.9 ± 12.2			
**SSYA10‐001**				NA^g^	NA^g^	1.8 ± 0.22	3.78 ± 1.5	

a EC_50_ = concentration of compound that gives 50% rescue of the virus‐reduced eGFP signals as compared to the untreated virus‐infected control cells.

b CC_50_ = 50% cytotoxic concentration, as determined by measuring the cell viability with the colorimetric formazan‐based MTS assay. The values represent the means ± SD of data derived from duplicate experiments.

c Compound concentration required to inhibit the SARS‐CoV‐2 nsp13 ATPase‐associated activity by 50%.

d Compound concentration required to inhibit the SARS‐CoV‐2 nsp13 helicase‐associated activity by 50%.

e Data from Nizi et al*.* [17]*.*

f Percentage of enzymatic residual activity.

g NA, not active.

All compounds were evaluated for their antiviral activity in SARS‐CoV‐2‐infected VeroE6 cells and cytotoxicity was assessed in mock‐infected VeroE6 cells. GS‐441524, the parent nucleoside of remdesivir and obeldesivir, was included as a positive control [25]. In parallel, the newly synthesized derivatives were also tested for inhibition of the two enzymatic functions of nsp13 (i.e. ATPase and unwinding activity), using the known nsp13 inhibitor, SSAY10−001 [26], as a reference. To rule out inhibition by nonspecific aggregators, assays were performed in the presence of bovine serum albumin (BSA) and tris(2‐carboxyethyl)phosphine (TCEP). Under these conditions, hit compound **2** retained its ability to inhibit helicase unwinding, albeit with a reduced potency (IC_50_ = 21.2 µM). and inhibited also the helicase ATPase activity with an IC_50_ value of 18.8 µM.

Concerning the modifications on the C‐4 substituent, with the exception of **4**, which showed a decent EC_50_ value of 7.18 µM, all other compounds (**3**, **5**, and **6**) were completely inactive against SARS‐CoV‐2, with some of them also showing toxicity. They were also unable to recognize the helicase, with only a residual ATPase activity for **3**, which indeed still maintained an intact DMTHI.

Different outcomes were observed following modifications at the C‐2 position, while maintaining the DMTHI group fixed at the C‐4 position. The removal of the C‐2 substituent, as in compound **7**, led to a completely inactive compound both in the enzymatic and cellular assays, while some toxicity appeared. Absence of antiviral activity was also observed for compound **10**, which lacks the *para* substituent on the phenyl ring, or in the bioisoster thiophene **8**. Nonetheless, these compounds inhibited at least one of the helicase activities: the unwinding activity for **10** (IC_50_ of 22.7 µM), and both the ATPase and unwinding activities for **8** with similar potencies (IC_50_s of 22.81 and 23.78 µM, respectively).

Conversely, introduction of the bulky naphthalene substituent at the C‐2 position afforded compound **9**, which was a potent antiviral agent (EC_50_ = 9.71 µM), but was, also, accompanied by cytotoxicity (CC_50_ = 14.74 µM). This derivative exhibited potent inhibition of the helicase ATPase activity (IC_50_ = 8.9 µM) and retained measurable activity in the unwinding assay (IC_50_ = 22.2 µM) Therefore, in all forthcoming compounds, a C‐2 phenyl ring was maintained, however, with different functionalization.

Shifting the propoxy group of compound **2** from the *para* to the *ortho* position gave **12**, which was completely inactive as an antiviral agent. However, it exhibited modest inhibition of both the ATPase and unwinding activities of the nsp13 helicase, with IC_50_ values of 19.47 and 20.08 µM, respectively. Among the compounds bearing a *para‐*substituent on the C‐2 phenyl ring, the presence of a hydroxy (**11**) or methoxy group (**13**) did not confer antiviral activity, but instead resulted in marked cytotoxicity. Conversely, elongation of the propoxy chain of compound **2** to an *n*‐butoxy group (**14**) or branching into an isopropoxy group (**15**) afforded potent antiviral compounds, with EC_50_ values of 8.06 and 9.11 µM, respectively, and favorable selectivity indices (>7.6 and >11, respectively). When the propoxy group was further elongated to 5 and 8 carbons (**16** and **17**, respectively), the antiviral activity was lost. Similarly unproductive was the introduction of aromatic (**19** and **20**) and (hetero)cycloalkyl (**18**, **21**) substituents as well as the mesylate group (**22**). On the other hand, compound **23**, characterized by the presence of an *n*‐butyl side chain, still maintained good antiviral activity (EC_50_ of 12.72 µM).

Notably, with the exception of morpholine derivative **21**, all compounds demonstrated interaction with helicase regardless of their antiviral activity. In particular, while **16**, **17** and **23** only inhibited the unwinding activity with IC_50_ approaching 20 µM, the other compounds (**12**, **14**, **15**, **18–** **20**, and **22**) inhibited both helicase activities, with IC_50_ values ranging from 5.15 to 19.47 µM for the ATPase activity and from 10.39 to 29.05 µM for the unwinding activity.

The replacement of the methoxy groups at positions 5 and 7 of the 2‐PhQ core by methyl groups (**24**) caused a complete loss of antiviral activity and a slight reduction of helicase inhibition. In contrast, shifting both methoxy groups to positions 5 and 8 furnished compound **25**, which has a profile which is superimposable to that of the hit **2**. Derivative **26**, combining structural features of **25** and **14**, emerged as a less potent anti‐SARS‐CoV‐2 compound when compared with its precursors, but was one of the most potent ATPase inhibitors with an IC_50_ value of 9.31 µM. Activity markedly decreased when the methoxy groups were shifted to positions 6 and 7 (**27**) or when they were elongated to an ethoxy group (**28**). The introduction of a third methoxy group on the phenyl ring, as in compound **29**, was also deleterious for the antiviral activity, although moderate enzyme inhibition persisted (ATPase IC_50_ = 23.4 µM and unwinding IC_50_ = 17.4 µM).

Overall, these findings confirm that the DMTHI moiety is the primary determinant of helicase inhibition while the substituents at the C‐2 position mainly govern the antiviral efficacy.

Compounds **14** and **15** demonstrated the most favorable anti‐SARS‐CoV‐2 profiles, effectively inhibiting both enzymatic activities of nsp13. Consequently, these derivatives underwent a series of in‐depth studies to better understand their potential for further optimization.

### 2.3 In‐Depth Investigation of Compounds 14 and 15

#### 2.3.1 Antiviral Spectrum of Action

To determine potential broad‐spectrum anti‐coronavirus activity, compounds **14** and **15** were both evaluated against HCoV‐229E (an *α*‐coronavirus) and HCoV‐OC43 (similar to SARS‐CoV‐2, also a *β*‐coronavirus). The nucleoside analog GS‐441524, included as positive control, displayed potent antiviral activity against both viruses, consistent with previous reports (Table 2) [17, 27].

**TABLE 2:** Antiviral activity and cytotoxicity of **14** and **15** against HCoVs 229E and OC43.

Compd.	HCoV‐229E EC_50_, µM^a^	HCoV‐OC43 EC_50_, µM^a^	HEL‐299 CC_50_, µM^b^
**14**	3.66 ± 1.25	24.02 ± 6.82	>50
**15**	2.47 ± 1.94	>50	>48.53
**GS‐441** **524**	0.9 ± 0.1	1.3 ± 0.07	>100

a EC_50_ = Effective concentration producing 50% inhibition of virus‐induced cytopathic effect, as determined by measuring the cell viability with the colorimetric formazan‐based MTS assay.

b CC_50_ = 50% cytotoxic concentration, as determined by measuring the cell viability with the colorimetric formazan‐based MTS assay. The values represent the means ± SD of data derived from triplicate experiments.

Both compounds **14** and **15** were nontoxic to the HEL‐299 cells at the highest tested concentration, but displayed distinct antiviral profiles. Compound **15** was active only against HCoV‐229E affording an IC_50_ value of 2.47 µM, slightly more potent than its activity against SARS‐CoV‐2. In contrast, derivative **14** inhibited the replication of both viruses with EC_50_ values of 3.66 and 24.02 µM against HCoV‐229E and HCoV‐OC43, respectively.

These findings indicate that the *n*‐butoxy substituent in compound **14** confers broader anti‐HCoVs activity when compared to the isopropoxy group in **15**.

#### 2.3.2 Time‐of‐Addition Assay

**FIGURE 3:**
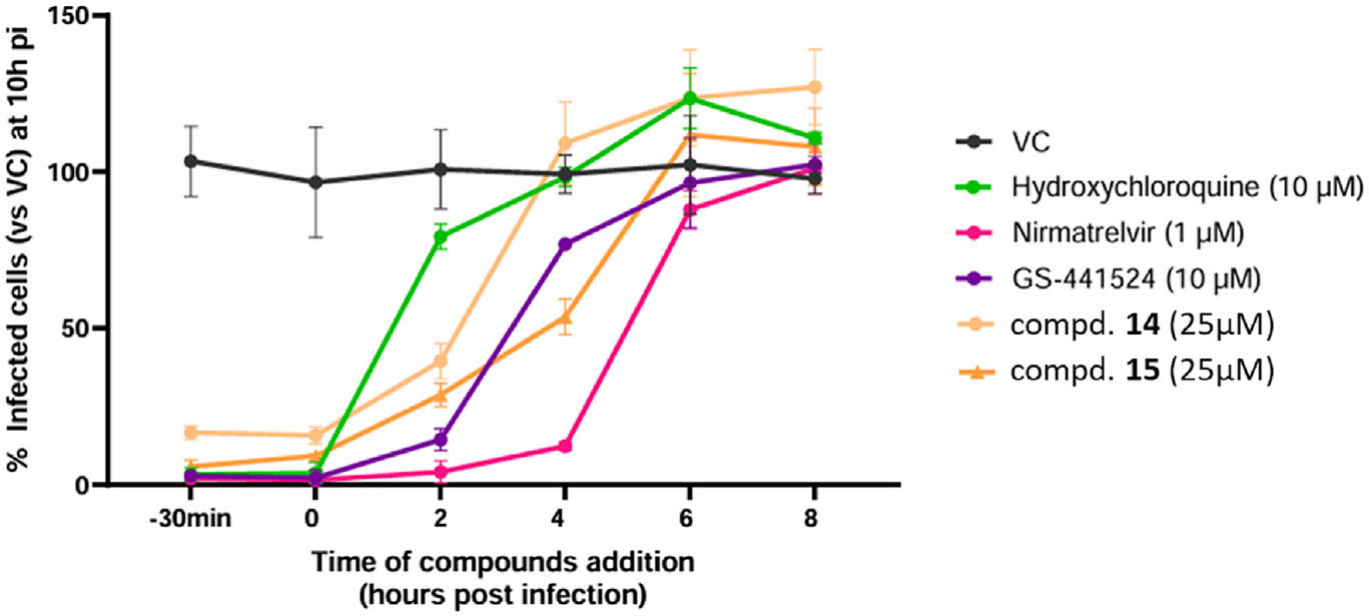
Time‐of‐addition assay for 2‐PhQs **14** and **15** and the controls hydroxychloroquine, nirmatrelvir and GS‐441524. VC = virus control.

As a further investigation of the mode of action of the 2‐PhQs, a TOA assay was performed for compounds **14** and **15** (Figure 3). In a single‐cycle replication assay (read‐out 10 h after infection), performed in VeroE6‐H2B‐mCherry cells infected with a SARS‐CoV‐2‐mNeonGreen reporter virus, the compounds were added at different time points (30 min before the infection, 2, 4, 6, and 8 h postinfection). Three reference compounds with an established mode of action were included in this study. Hydroxychloroquine, blocking the SARS‐CoV‐2 endosomal entry, which is the dominant entry pathway in VeroE6 cells, only shows antiviral effect when added prior to or at the time of infection. The addition of GS‐441524 (a RdRp inhibitor) and nirmatrelvir (a Mpro inhibitor) could be postponed to 2 and 4 h after infection, respectively. In contrast, compounds **14** and **15** exhibited reduced antiviral efficacy when administered 2 h or later after infection. This pattern mirrors that of GS‐441524, consistent with the role of nsp13 helicase within the RTC alongside RdRp.

#### 2.3.3 Binding Mode Analysis

The ATP‐binding pocket of SARS‐CoV‐2 helicase, which is located at the interface of the two RecA‐like domains, is highly conserved and essential for catalysis. It features residues that coordinate ATP binding, magnesium ion stabilization, and hydrolysis, including the phosphate‐binding and aspartate‐containing motifs, as well as the arginine finger, providing a structural framework for ligand interactions. Given its structural conservation and central role in coupling chemical energy to RNA translocation [16], the ATPase site was selected for detailed investigation. In this study, we conducted comparative molecular docking and interaction analyses to explain the binding profiles of our ligand set. The computational comparison was therefore designed to identify structural determinants that differentiate (a) ligands that effectively block ATPase catalysis (exemplified by **19**, the most potent nsp13 ATPase inhibitor), (b) ligands with strong antiviral efficacy (**14** and **15**), and (c) ligands that lack both enzymatic and antiviral activity (**5**, **7**, and **21**).

**FIGURE 4:**
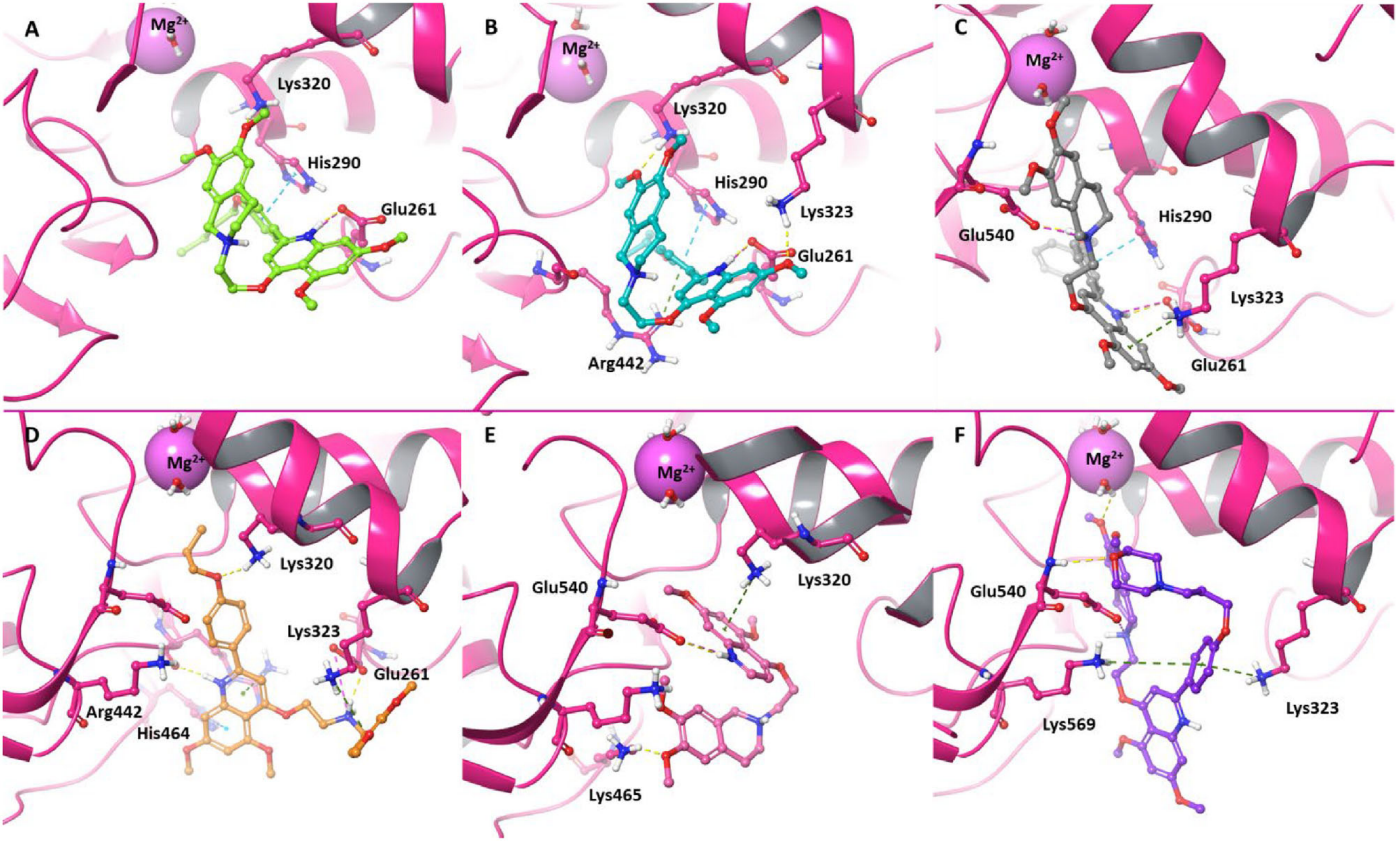
3D representation of the best docking pose of (A) **14** (green carbon sticks), (B) **15** (teal carbon sticks), (C) **19** (gray carbon sticks), (D) **5** (orange carbon sticks), (E) **7** (rose carbon sticks), and (F) **21** (violet carbon sticks) into the SARS‐CoV‐2 nsp13 pocket 3. The enzyme and the residues involved in crucial contacts with the compounds are shown as salmon pink carbon sticks, respectively.

Docking poses and interaction fingerprints were inspected with particular attention to contacts with catalytic and auxiliary residues previously implicated in nucleotide recognition and catalysis. Recent crystallographic data of SARS‐CoV‐2 nsp13 helicase bound to ATP and ADP revealed a highly organized nucleotide‐binding pocket, where the purine ring is sandwiched between His290 (*π*–*π* stacking) and Arg442 (cation–*π* and donor–*π* contacts, plus an H‐bond to Ser264) [28]. Within this framework, compounds **14** and **15** closely mimic the native nucleotide binding mode. Compound **14** establishes a salt bridge between the quinolinic NH and Glu261, a *π*–*π* interaction with His290, and a hydrogen bond between the tetrahydroisoquinoline methoxy oxygen and Lys320 (Figure 4A). Additionally, compound **15** forms a further H‐bond with Lys323 and a *π*–cation interaction with Arg442 (Figure 4B). Compound **19**, although inactive in cell‐based assays, adopts a binding geometry that perturbs key residues involved in ATP hydrolysis, consistent with its strong inhibition of isolated ATPase activity. Indeed, in addition to contacts with His290, Lys323, and Glu261, the DMTHI ring forms a stabilizing hydrogen bond with Glu540, further anchoring the ligand within the catalytic cleft (Figure 4C). Conversely, when compounds **5**, **7**, and **21** bind to the active site, interactions with the Lys and Arg networks are maintained, but the contact with His290 is lost in all three compounds, and the interaction with Glu261 is additionally absent in **21** and **7** (Figure 4D–F). His290, located near the P‐loop, is critical for stabilizing the transition state during ATP hydrolysis, participating in a proton relay network and potentially acting as a general base in concert with the Walker B motif. Loss of interaction with His290 in inactive compounds severely compromises proper substrate alignment and transition‐state stabilization. Although Glu261, which is more distant from the Mg^2+^ center, helps maintain the electrostatic balance of the catalytic cavity and orients the triphosphate group, preservation of this contact does not compensate for the critical loss of that with His290, explaining the reduced affinity of the inactive analogs.

#### 2.3.4 Determination of Kinetics of nsp13 Inhibition

**FIGURE 5:**
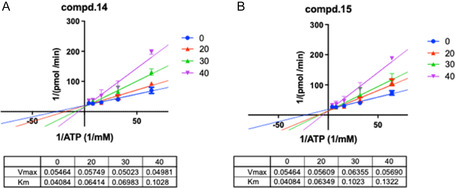
Lineweaver–Burk analysis of the kinetics of (A) the nsp13‐associated ATPase activity in the absence or presence of compound **14** 20 μM (red line), 30 μM (green line), and 40 μM (purple line). (B) Kinetics of the nsp13‐associated ATPase activity in the absence (blue line) or presence of compound **15** 20 μM (red line), 30 μM (green line), and 40 μM (purple line).

Considering the major activity of the 2‐PhQs on the ATPase activity of the enzyme, as further supported by in silico analyses, we assessed the kinetics of inhibition of the nsp13‐associated ATPase activity by titrating the ATP in the reaction mixture in the absence or presence of an increasing amount of inhibitors **14** and **15**. Results analyzed by a Lineweaver–Burk plot, reported in Figure 5, showed an increase in Km for both compounds along with a constant *V*_max_ (approximately 0.5 nmol/min), suggesting that both compounds inhibited the enzyme by competing with the natural substrate ATP for its binding in the active site.

#### 2.3.5 Cytotoxicity Profiling

The cytotoxic profile of compounds **14** and **15** in two different cell lines was evaluated. Since HCoVs, including SARS‐CoV‐2, show a high tropism for the bronchial epithelium, we first investigated the compounds on BEAS‐2B cells (Figure S16). Neither compound **14** nor compound **15** showed any signs of cytotoxicity on BEAS‐2B cells at any concentrations tested (from 5 to 100 µM). In contrast, both compounds provoked a small increase of BEAS‐2B viability which is significant, if compared with vehicle, at 10 and 50 µM for **14** (% cell viability of vehicle: 100.0 ± 2.7; **14**, 10 µM: 138.1 ± 3.8; **14**, 50 µM: 149.3 ± 1.8), and at 5 and 10 µM for **15** (% cell viability of vehicle: 100.0 ± 2.7; **15**, 5 µM: 122.7 ± 4.5; **15**, 10 µM: 131.4 ± 8.2). Moreover, both **14** and **15** did not affect the reactive oxygen species (ROS) amount in BEAS‐2B cells compared to the vehicle. We also evaluated the cytotoxic profile of the same derivatives on H9c2 cells (Figure S17), representing a recognized cardiac in vitro model, for the known cardiac effects of SARS‐CoV‐2 infections. In this cell line, both compounds were nontoxic up to 10 µM, while they showed a reduction of viability, compared to the vehicle, at the highest tested concentrations of 50 and 100 µM (% Cell viability of vehicle: 100.0 ± 2.4; **14**, 50 µM: 50.5 ± 4.5; **14**, 100 µM: 47.9 ± 4.9) (% Cell viability of vehicle: 100.0 ± 2.4; **15**, 50 µM: 61.3 ± 4.9; **15**, 100 µM: 56.8 ± 7.0) and at the same concentrations, also a consequent increase in ROS production was recorded (% ROS production of vehicle: 100.0 ± 2.7; **14**, 50 µM: 138.1 ± 6.7; **14**, 100 µM: 136.1 ± 8.0) (% ROS production of vehicle: 100.0 ± 2.7; **15**, 50 µM: 131.8 ± 8.1; **15**, 100 µM: 123.4 ± 4.7). However, because compounds **14** and **15** exerted their antiviral effects at low single‐digit µM concentrations, a high safety margin is still guaranteed in H9c2 cells.

#### 2.3.6 Preliminary In Vitro PK Evaluation of Compound 15

To verify its suitability for in vivo studies, the key pharmacokinetic properties of compound **15** were determined. These properties include solubility, membrane permeability, plasma stability and liver metabolic stability.

Solubility of **15** in aqueous solutions with 1% DMSO was assessed using nephelometric measurements. The compound was completely soluble (Figure S18) at 10 µM in phosphate‐buffered saline (PBS) within a time frame of 0, 30, 60, 90, and 120 min.

Membrane permeability was determined via the Parallel Artificial Membrane Permeability Assay (PAMPA) [29, 30], employing an *l*‐*α*‐phosphatidylcholine lipid membrane. Propranolol and caffeine were used as high and medium permeability controls, respectively. Furosemide was used as the negative control. The apparent permeability coefficient (*P*_app_) was calculated using the Faller‐modified Sugano equation [31]. Compound **15** showed a good P_app_ of 4.83 × 10^−6^ cm/s, higher than the medium permeability standard caffeine (Table S1).

The metabolic stability of compound **15** in the presence of both human and mouse liver microsomes (HLMs and MLMs, respectively) was determined. Testosterone, included as a reference compound, showed a half‐life below 40 and 15 min, upon incubation with HLMs and MLMs, respectively. Incubation without NADPH showed no NADPH‐independent metabolism or nonenzymatic degradation, with 101.7% ± 2.6% of the initial concentration remaining after 60 min. Compound **15** exhibited a time‐dependent disappearance during microsomal incubation (Figure S19A), with a t_1/2_ of 179.3 min and a CL_int_ of 9.7 mL·min^−1^·kg^−1^ following incubation with HLMs. In contrast, **15** showed greater susceptibility to metabolic degradation after incubation with MLMs (Figure S19B), as evidenced by a 40% reduction in half‐life (110.0 min) and a consequent fivefold increase in intrinsic hepatic clearance (CL_int_: 49.6 mL·min^−1^·kg^−1^). The progressive disappearance of the parent compound over time was accompanied by the formation of several metabolites in a time‐dependent manner (Figure S19C,D).

LC‐MS/MS analysis revealed that the primary metabolites of compound **15** were derivatives generated through terminal desaturation (**M1**) and hydroxylation (**M2**) reactions. The predominant metabolite (**M1**) was formed through oxidative desaturation of the isopropyl group within the isopropoxy moiety, resulting in the generation of an alkyl vinyl derivative (Figure S20A) with a precursor ion [M + H]^+^ at m/z 557.26 (C_33_H_37_N_2_O_6_^+^, RT: 2.97 min, Figure S20C). The **M2** metabolite (Figure S20B) exhibited a protonated precursor ion [M + H]^+^ at m/z 575.27 (C_33_H_39_N_2_O_7_^+^, RT: 2.80 min), corresponding to a +16 Da mass shift relative to the parent compound. Detailed analysis of the MS/MS fragmentation spectra indicated that hydroxylation predominantly occurred on the DMTHI moiety, as suggested by the presence of a key fragment ion at m/z 236.13 (C_13_H_18_NO_3_^+^, Figure S20D). Minor metabolites were generated by *O*‐dealkylation of compound **15**, involving both the 5,7‐methoxy groups (**M3**, m/z 545.26, C_32_H_37_N_2_O_6_^+^, RT: 2.72–2.76 min, Figure S21A,C) and the isopropoxy (**M4**, m/z 517.23, C_30_H_33_N_2_O_6_^+^, RT: 2.25 min, Figure S21B,D) moieties.

Hepatic biotransformation can significantly alter a compound's physicochemical properties but may also generate reactive or toxic metabolites, increasing the risk of adverse effects. Early identification of these metabolites is crucial for minimizing toxicological risks and ensuring safety in drug development.

To investigate reactive metabolite formation, we performed a trapping assay by incubating the test compound with liver microsomes in the presence of the nucleophile glutathione (GSH). This assay captures reactive intermediates as stable GSH conjugates, which are then analyzed LC‐MS. Acetaminophen and diclofenac were used as reference compounds. The formation of GSH conjugates was confirmed for both, with the corresponding adducts detected at m/z 457 and 617, respectively, thereby validating the sensitivity and reliability of the trapping assay [32, 33]. In contrast, no GSH‐conjugated species were detected after incubation of **15** with liver microsomes, indicating that the compound did not form reactive electrophilic intermediates able to react with GSH. These findings suggest that **15** does not undergo metabolic bioactivation into potentially toxic species under the tested conditions, supporting its favorable metabolic safety profile.

## 3 Conclusion

Undoubtedly, the discovery of new antiviral drugs has acceleration compared to the past, with about 39 new direct‐acting antivirals that have been approved by the U.S. FDA in the last ten years [34]. However, the current antiviral arsenal is still characterized by numerous limitations, including its efficacy against only a small number of viruses, leaving many other viral infections without an effective treatment. CoVs are among the viruses that do not yet have an adequate remedy. One way to identify new drugs that are active against resistant strains and show a broad spectrum of action is to inhibit highly conserved targets that have not yet been tackled by currently available drugs. The nsp13 helicase, which is essential for viral replication and highly conserved among CoVs, addresses these requirements.

We recently reported compound **2**, based on a 2‐PhQ scaffold, as one of the few nsp13 inhibitors that also displayed antiviral activity.

In this article, a series of new analogs was prepared that enriched the SAR around the 2‐PhQ scaffold, which was confirmed as appropriate to impart nsp13 helicase inhibition, especially when a DMTHI moiety is present at the C‐4 position of the quinoline core. Notably, almost all the compounds having this moiety inhibit one or both of the nsp13 activities. However, the enzyme inhibition did not always directly correlate with the antiviral activity, with only a few compounds that were also able to inhibit viral replication, specifically when a medium‐length *O*‐alkyl group is positioned at the *para* position of the 2‐phenyl ring. Additional analogs will certainly need to be prepared to define the optimal substitution pattern of the quinoline core that is able to impart potent antiviral activity through this innovative mechanism of action. In particular, we will try to boost nsp13 inhibition in order to recover antiviral activity, guided by computational studies.

In summary, this study led to the identification of new 2‐PhQs derivatives, such as **14** and **15**, which showed a promising profile. TOA experiments and kinetic analysis confirmed the helicase as the main target underlying their antiviral activity, by competing with ATP, as suggested by docking experiments. They are also active against other HCoVs, thus emerging as having a broad spectrum of antiviral action. The safety assessment of the two compounds also highlighted their suitability, as they were found to be nontoxic not only on two different cell lines (VeroE6 and HEL cells), but also on bronchial epithelium and cardiac cell models, where cell viability and ROS production remained unaffected. Preliminary PK properties of compound **15** showed good apparent permeability, as well as a favorable metabolic profile.

## 4 Material and Methods

### 4.1 Chemistry

#### 4.1.1 General Chemistry

All starting materials, reagents and solvents were purchased from common commercial suppliers and were used as such, unless otherwise indicated. Organic solutions were dried over anhydrous Na_2_SO_4_ and concentrated with a rotary evaporator at low pressure. All reactions were routinely checked by thin‐layer chromatography (TLC) on silica gel 60_F254_ (Merck) and visualized by using UV or iodine. Flash chromatography separations were carried out on Merck silica gel 60 (mesh 230–400). Yields were those of purified products and were not optimized. Bruker Avance DRX‐400 and DRX‐600 (Bruker Corporation, Massachusetts, USA) were used to record ^1^H NMR and ^13^C NMR spectra at 400 and 101 MHz, and at 600 and 150 MHz, respectively. Chemical shifts are given in ppm (δ) relative to TMS. Spectra were acquired at 298 K unless otherwise indicated. Data processing was performed with standard Bruker software TopSpin (Vers. 4.1.4) and the spectral data are consistent with the assigned structures. The purity of the tested compounds (>95% sample purity) was evaluated by HPLC analysis using a Jasco LC‐4000 instrument equipped with a UV–visible Diode Array Jasco MD‐4015 and an XTerra MS C18 Column, 5 μm, 4.6 mm × 150 mm (flow: 1 mL/min). Chromatograms were analyzed by ChromNAV 2.0 Chromatography Data System software. For each compound, the method used for the analysis is specified and the peak retention time (tR) is given in minutes. Detection mass was based on electrospray ionization (ESI) in positive polarity using an Agilent 1290 Infinity System equipped with a MS detector Agilent 6540UHD Accurate Mass Q‐TOF.

The target compound **27**, along with the intermediates **30**, **32**, **45**, **52**, **62**, **63**, **69**, **73**, **78**, and **83** were synthesized according to the already reported procedures [17, 23].

**General procedure (A) for the synthesis of compounds 34–40**. Under nitrogen atmosphere, to a suspension of **33** (1 eq) in dry THF (20 mL), Et_3_N (2 eq) was slowly added in 15 min at 0°C. To the mixture, a solution of properly substituted benzyl chloride (1.1 eq) in dry THF (2 mL ×mmol) was then added drop wise. The reaction was stirred at r.t for 12 h. The mixture was concentrated under vacuum and then poured in ice/water to give a precipitate that was filtered, obtaining the desired intermediates.

**General procedure (B) for the synthesis of compounds 41–47**. Under nitrogen atmosphere, to a solution of the proper intermediate **34–** **40** (1 eq) in dry CH_2_Cl_2_ (5 mL × mmol), SnCl_4_ (4 eq) and a solution of acetyl chloride (4 eq) in dry CH_2_Cl_2_ (1 mL × mmol) were added at 0°C. The reaction mixture was stirred at r.t. for 24 h, then poured in 2 N HCl solution and extracted with CH_2_Cl_2_ (×3). The organic layer was washed with brine, dried over Na_2_SO_4_ and evaporated to dryness under reduced pressure to obtain the desired intermediates.

**General procedure (C) for the synthesis of compounds 48–54**. To a solution of the proper intermediate **41–47** (1 eq) in *t‐*BuOH (10 mL × mmol), *t‐*BuOK (5 eq) was added at 0°C. The reaction mixture was stirred at rt/50°C for 12 h and then concentrated under vacuum and poured in 2N HCl solution. The formed precipitate was filtered to obtain the desired compounds.

**General procedure (D) for the synthesis of compounds 6**, **20**, **31**, **55–61**, **81–85**, and **87**. Under N_2_ atmosphere, to a suspension of the proper intermediate **30**, **48–54**, **76–** **80**, or **11** (1 eq) and K_2_CO_3_ (5 eq) in dry DMF (5 mL × mmol), 1‐bromo‐2‐chloroethane (4 eq) or 1‐bromo‐3‐chloropropane (4 eq) or 2‐chloroethyl benzol (4 eq) or 4‐(2‐chloroethyl)−1,2‐dimethoxybenzene (4 eq) were added. The reaction was stirred at 80°C for 2–24 h. The mixture was then poured into ice/water and extracted with EtOAc (x3). The organic layer was washed with brine, dried over Na_2_SO_4_ and evaporated to dryness under reduced pressure. The obtained residues were purified by flash column chromatography to give target compounds **6** and **20** and the intermediates **31**, **55–61**, **81–85**, and **87**.

**General procedure (E) for the synthesis of compounds 3–** **5**, **7**‐ **10**, **12–** **14**, **19**, **23–** **26**, **28**, and **29**. Under N_2_ atmosphere, to a solution of the proper intermediate **31**, **32**, **55–61**, **64**, **81–85**, or **87** (1 eq) in dry DMF (2 mL × mmol), K_2_CO_3_ (6 eq) and various nitrogen‐based heterocycles (3 eq) were added. The mixture was stirred at 80°C for 3–12 h. The mixture was then poured into ice/water and extracted with EtOAc (x3). The organic layers were washed with brine, dried over Na_2_SO_4_ and evaporated to dryness under reduced pressure. The target compounds were obtained after purification by flash column chromatography as described for each compound.

**General procedure (F) for the synthesis of compounds 71–75.** To a solution of the proper intermediate **68**, **67** [23], or **70** (1 eq) in dry benzene (5 mL × mmol), 3,5‐dimethoxy aniline or 2,5‐dimethoxy aniline or 2,5‐diethoxy aniline (5 eq) and *p*‐TsOH (20% w/w) were added. The reaction mixture was stirred at reflux using a Dean–Stark apparatus for 24 h. Then the reaction mixture was concentrated under vacuum, poured in 2 N HCl solution and extracted with CH_2_Cl_2_ (x3). The organic layers were washed with brine, dried over Na_2_SO_4_ and evaporated to dryness under reduced pressure. The desired intermediates were obtained after purification by flash chromatography column, as described for each compound.

**General procedure (G) for the synthesis of compounds 76–80**. The proper intermediate **71–75** (1 eq) was reacted in Dowtherm A (2 mL × mmol) at 240°C for 1.5 h. After cooling at rt, the mixture was treated with cyclohexane, obtaining the desired intermediate as a solid recovered by filtration.

**General procedure (H) for the synthesis of compounds 15–** **18**, **21**, and **22**. Under nitrogen atmosphere, to a solution of compound **11** (1 eq) in dry DMF (1 mL × mmol), K_2_CO_3_, or Cs_2_CO_3_ (6 eq) and properly halogen derivatives or properly sulfonyl derivatives (4 eq) were added. The reaction was stirred at 80°C for 1–12 h, then poured into ice/water and extracted with EtOAc (×3). The organic layer was washed with brine, dried over Na_2_SO_4_ and evaporated to dryness under reduced pressure. The desired intermediates were obtained after purification by flash chromatography column, as described for each compound.

**4‐[3‐(6,7‐Dimethoxy‐3,4‐dihydroisoquinolin‐2(1H)‐yl)propoxy]−5,7‐dimethoxy‐2‐(4‐propoxyphenyl)quinoline** (**3**). General procedure (**E**), starting from **31** and using 6,7‐dimethoxytetrahydroisoquinoline, time = 5 h. After purification by flash column chromatography (CHCl_3_/MeOH 95:5), compound **3** was obtained as a solid in 72% yield. ^1^H NMR (400 MHz, CDCl_3_) δ: 1.09 (3H, t, *J* = 7.4 Hz, OCH_2_CH_2_ *CH*_3_), 1.84–1.89 (2H, m, OCH_2_ *CH*_2_CH_3_), 2.24–2.28 (2H, m, CH_2_), 2.82–2.87 (6H, m, tetrahydroisoquinoline CH_2_, tetrahydroisoquinoline CH_2_, CH_2_N), 3.66 (2H, s, tetrahydroisoquinoline CH_2_), 3.86 (3H, s, OCH_3_), 3.87 (3H, s, OCH_3_), 3.95 (3H, s, OCH_3_), 3.96 (3H, s, OCH_3_), 4.01 (2H, t, *J* = 6.6 Hz, O*CH*_2_CH_2_CH_3_), 4.34 (2H, t, *J* = 6.0 Hz, OCH_2_), 6.48 (1H, d, *J* = 2.2 Hz, H6), 6.55 (1H, s, tetrahydroisoquinoline H), 6.63 (1H, s, tetrahydroisoquinoline H), 7.00–7.03 (3H, m, H3’, H5’, and H3), 7.07 (1H, d, *J* = 2.3 Hz, H8), 8.04 (2H, d, *J* = 8.8 Hz, H2’ and H6’). ^13^C NMR (101 MHz, CDCl_3_) δ: 10.65, 22.69, 27.11, 28.80, 51.30, 55.00, 55.63, 56.00, 56.14, 66.92, 69.68, 97.61, 98.14, 100.69, 107.41, 109.48, 111.38, 114.66, 126.25, 126.60, 128.77, 132.40, 147.29, 147.60, 153.29, 158.05, 160.34, 160.99, and 164.22. HPLC acquisition time = 15 min with a gradient consisting of ACN and water containing 0.1% diethylamine, with a linear increase of ACN from 10% to 100%, tR = 4.08 min. HRMS: m/z calcd for C_34_H_40_N_2_O_6_ 573.2964 [M + H^+^], found 573.2963.

**4‐[2‐(5,6‐Dimethoxy‐1,3‐dihydro‐2H‐isoindol‐2‐yl)ethoxy]−5,7‐dimethoxy‐2‐(4‐propoxyphenyl)quinoline** (**4**). General procedure (**E**), starting from **32** [23] and using 5,6‐dimethoxy‐2,3‐dihydro‐1H‐isoindole hydrochloride, time = 5 h. After purification by flash column chromatography (CHCl_3_/MeOH 99:1), compound **4** was obtained as a gray solid in 10% yield. ^1^H NMR (600 MHz, CDCl_3_) δ_H_: 0.99 (3H, t, *J* = 7.4 Hz, OCH_2_CH_2_ *CH*_3_), 1.73–1.82 (2H, m, OCH_2_ *CH*_2_CH_3_), 3.33 (2H, t, *J* = 4.9 Hz, CH_2_N), 3.79 (6H, s, OCH_3_), 3.91 (3H, s, OCH_3_), 3.93 (3H, s, OCH_3_), 3.94 (2H, t, *J* = 6.6 Hz, O*CH*_2_CH_2_CH_3_), 4.12–4.14 (4H, m, isoindole CH_2_x2), 4.40 (2H, t, *J* = 4.9 Hz, OCH_2_), 6.41 (1H, d, *J* = 2.3 Hz, Ar‐H), 6.69 (2H, s, isoindole Hx2), 6.93–6.96 (2H, m, H3’, Ar‐H), 7.06 (1H, s, Ar‐H), 7.97 (2H, d, *J* = 8.8 Hz, Ar‐H). ^13^CNMR (150 MHz, CDCl_3_) δ_C_: 10.53, 22.59, 29.71, 54.39, 55.57, 55.95, 56.17, 60.00, 68.38, 69.64, 97.62, 98.20, 100.59, 105.79, 107.27, 114.67, 124.83, 128.74, 131.27, 132.06, 148.60, 153.11, 157.84, 158.62, 160.40, 161.05, and 163.81 ppm. HPLC acquisition time = 15 min. with a gradient consisting of ACN and water containing 0.1% diethylamine, with a linear increase of ACN from 20% to 100%, tR = 9.96 min. HRMS: m/z calcd for C_32_H_36_N_2_O_6_ 545.2651 [M + H^+^], found 545.2648.

**N‐(3,4‐dimethoxybenzyl)‐2‐{[5,7‐dimethoxy‐2‐(4‐propoxyphenyl)quinolin‐4‐yl]oxy}ethanamine** (**5**). General procedure (**E**) starting from compound **32** [23] and using 3,4‐dimethoxybenzyl amine, time = 12 h. After purification by flash column chromatography (CHCl_3_/MeOH 99:1), compound **5** was obtained as a white solid in 60% yield. ^1^H NMR (600 MHz, CDCl_3_) δ: 1.07 (3H, t, *J* = 7.4 Hz, OCH_2_CH_2_ *CH*_3_), 1.83–1.86 (2H, m, OCH_2_ *CH*_2_CH_3_), 4.77 (2H, t, *J* = 4.7 Hz, CH_2_N), 3.77 (3H, s, OCH_3_), 3.85 (3H, s, OCH_3_), 3.89 (3H, s, OCH_3_), 3.89 (2H, s, benzylic CH_2_), 3.93 (3H, s, OCH_3_), 3.99 (2H, t, *J* = 6.5 Hz, O*CH*_2_CH_2_CH_3_), 4.35 (2H, t, *J* = 4.7 Hz, OCH_2_), 6.42 (1H, d, *J* = 1.5 Hz, Ar‐H), 6.82 (1H, d, *J* = 8.0 Hz, Ar‐H), 6.90 (1H, d, *J* = 7.9 Hz, Ar‐H), 6.95 (1H, s, Ar‐H), 6.99–7.02 (3H, m, Ar‐H), 7.05 (1H, d, *J* = 1.5 Hz, Ar‐H), 8.02 (2H, d, *J* = 8.5 Hz, Ar‐H). ^13^CNMR (150 MHz, CDCl_3_) δ_C_: 10.51, 22.58, 47.93, 53.53, 55.52, 55.85, 55.94, 55.99, 67.59, 69.62, 97.66, 98.20, 100.70, 107.29, 111.03, 111.39, 114.64, 120.22, 128.67, 132.24, 132.84, 148.13, 149.06, 153.20, 157.80, 158.67, 160.33, 160.98, and 163.79 ppm. HPLC acquisition time = 15 min with a gradient consisting of ACN and water containing 0.1% diethylamine, with a linear increase of ACN from 20% to 100%, tR = 9.54 min. HRMS: m/z calcd for C_31_H_36_N_2_O_6_ 533.2651 [M + H^+^], found 533.2652.

**4‐[2‐(3,4‐Dimethoxyphenyl)ethoxy]−5,7‐dimethoxy‐2‐(4‐propoxyphenyl)quinoline** (**6**). General procedure (**D**) starting from **30** [23] and using 4‐(2‐chloroethyl)−1,2‐dimethoxybenzene, time 2 h. After purification by flash column chromatography (CH_2_Cl_2_/MeOH 99:1), compound **6** was obtained as a white solid in 50% yield. ^1^H NMR (400 MHz, CDCl_3_) δ: 1.06 (3H, t, *J* = 7.4 Hz, OCH_2_CH_2_ *CH*_3_), 1.79–1.88 (2H, m, OCH_2_ *CH*_2_CH_3_), 3.22 (2H, t, *J* = 6.7 Hz, OCH_2_ *CH*_2_), 3.86 (3H, s, OCH_3_), 3.87 (3H, s, OCH_3_), 3.92 (3H, s, OCH_3_), 3.94 (3H, s, OCH_3_), 4.04 (2H, t, *J* = 4.8 Hz, O*CH*_2_CH_2_CH_3_), 4.39 (2H, t, *J* = 6.7 Hz, OCH_2_CH_2_), 6.46 (1H, d, *J* = 2.3 Hz, Ar‐H), 6.83 (1H, d, *J* = 7.0 Hz, Ar‐H), 6.93–7.00 (5H, m, Ar‐H), 7.05 (1H, d, *J* = 2.2 Hz, Ar‐H), 8.01 (2H, d, *J* = 8.8 Hz, Ar‐H). ^13^C NMR (100 MHz, CDCl_3_) δ_C_: 10.52, 22.59, 35.24, 55.54, 55.88, 55.96, 56.09, 69.62, 69.75, 97.57, 98.29, 100.62, 107.31, 111.28, 112.48, 114.62, 121.15, 128.70, 130.89, 132.15, 147.88, 149.00, 153.16, 157.94, 158.54, 160.35, 161.03, 163.92 ppm. HPLC acquisition time = 15 min. with a gradient consisting of ACN and water containing 0.1% formic acid, with a linear increase of ACN from 20% to 100%, tR = 6.01 min. HRMS: m/z calcd for C_30_H_33_NO_6_ 504.2386 [M + H^+^], found 504.2386.

**4‐[2‐(6,7‐Dimethoxy‐3,4‐dihydroisoquinolin‐2(1*H*)‐yl)ethoxy]−5,7‐dimethoxyquinoline**(**7**). General procedure (**E**) starting from **64** and using 6,7‐dimethoxytetrahydroisoquinoline hydrochloride, time = 5 h. After purification by flash column chromatography (CHCl_3_/MeOH 98:2), compound **7** was obtained as a solid in 25% yield. ^1^H NMR (400 MHz, CDCl_3_) δ: 2.88–2.90 (2H, m, tetrahydroisoquinoline CH_2_), 2.99–3.00 (2H, m, tetrahydroisoquinoline CH_2_), 3.13–3.15 (2H, m, CH_2_N), 3.81 (2H, s, tetrahydroisoquinoline CH_2_), 3.85 (3H, s, OCH_3_), 3.87 (3H, s, OCH_3_), 3.95 (6H, s, OCH_3_), 4.37 (2H, m, OCH_2_), 6.52–6.53 (2H, m, Ar‐H), 6.63–6.65 (2H, m, Ar‐H), 7.02 (1H, s, Ar‐H), 8.59 (1H, d, *J* = 5.2 Hz, Ar‐H). ^13^C NMR (100 MHz, CDCl_3_) δ_C_: 28.80, 51.83, 55.57, 56.03, 56.33, 56.63, 68.03, 98.63, 100.44, 108.83, 109.51, 111.50, 126.02, 126.54, 147.36, 147.70, 151.72, 153.29, 158.04, 160.93, and 163.41 ppm. HPLC: acquisition time = 10 min. with a gradient consisting of ACN and water containing 0.1% diethylamine, with a linear increase of ACN from 10% to 100%, tR = 3.69 min. HRMS: m/z calcd for C_24_H_28_N_2_O_5_ 425.2076 [M + H^+^], found 425.2072.

**4‐[2‐(6,7‐Dimethoxy‐3,4‐dihydroisoquinolin‐2(1*H*)‐yl)ethoxy]−5,7‐dimethoxy‐2‐(2‐thienyl)quinoline** (**8**). General procedure (**E**) starting from compound **55** and using 6,7‐dimethoxytetrahydroisoquinoline, time = 12 h. After purification by flash column chromatography (CHCl_3_/acetone 98:2), compound **8** was obtained as a solid in 40% yield. ^1^H NMR (400 MHz, CDCl_3_) δ: 2.91 (2H, t, *J* = 5.1 Hz, tetrahydroisoquinoline CH_2_), 3.01 (2H, t, *J* = 5.9 Hz, tetrahydroisoquinoline CH_2_), 3.18 (2H, t, *J* = 5.6 Hz, CH_2_N), 3.83 (2H, s, tetrahydroisoquinoline CH_2_), 3.85 (3H, s, OCH_3_), 3.87 (3H, s, OCH_3_), 3.94 (3H, s, OCH_3_), 3.97 3H, s, OCH_3_), 4.45 (2H, t, *J* = 5.6 Hz, OCH_2_), 6.48 (1H, d, *J* = 2.3 Hz, Ar‐H), 6.55 (1H, s, tetrahydroisoquinoline H), 6.64 (1H, s, tetrahydroisoquinoline H), 7.03 (1H, d, *J* = 2.3 Hz, Ar‐H), 7.06 (1H, s, Ar‐H), 7.15 (1H, dt, *J* = 3.7 and 1.3 Hz, thienyl H4), 7.46 (1H, dd, *J* = 0.9 and 5.0 Hz, Ar‐H), 7.67 (1H, dd, *J* = 0.9 and 3.7 Hz, Ar‐H). ^13^C NMR (100 MHz, CDCl_3_) δ_C_: 27.70, 50.79, 54.54, 54.90, 54.93, 55.23, 55.58, 66.88, 95.53, 97.36, 99.53, 106.63, 108.43, 110.41, 124.34, 124.91, 125.41, 126.87, 127.12, 144.41, 146.27, 146.61, 152.08, 152.66, 156.83, 160.10, and 162.73 ppm. HPLC acquisition time = 10 min. with a gradient consisting of ACN and water containing 0.1% diethylamine, with a linear increase of ACN from 10 to 100%, tR = 3.24 min. HRMS: m/z calcd for C_28_H_30_N_2_O_5_S 507.1953 [M + H^+^], found 507.1953.

**4‐[2‐(6,7‐Dimethoxy‐3,4‐dihydroisoquinolin‐2(1*H*)‐yl)ethoxy]−5,7‐dimethoxy‐2‐(2‐naphthyl)quinoline** (**9**). General procedure (**E**) starting from compound **56** and using 6,7‐dimethoxytetrahydroisoquinoline, time = 12 h. After purification by flash column chromatography (CHCl_3_/MeOH 99:1), compound **9** was obtained as a solid in 25% yield. ^1^H NMR (400 MHz, CDCl_3_) δ: 2.89–2.91 (2H, m, tetrahydroisoquinoline CH_2_), 3.01–3.03 (2H, m, tetrahydroisoquinoline CH_2_), 3.20–3.21 (2H, m, CH_2_N), 3.85 (5H, s, tetrahydroisoquinoline CH_2_ and OCH_3_), 3.87 (3H, s, OCH_3_), 3.98 (3H, s, OCH_3_), 3.99 (3H, s, OCH_3_), 4.51–4.53 (2H, m, OCH_2_), 6.53 (1H, s, tetrahydroisoquinoline H), 6.56 (1H, s, tetrahydroisoquinoline H), 6.64 (1H, s, Ar‐H), 7.16 (1H, s, Ar‐H), 7.29 (1H, s, Ar‐H), 7.54–7.55 (2H, m, Ar‐H), 7.89–7.91 (1H, m, Ar‐H), 7.98–7.99 (2H, m, Ar‐H), 8.27–8.29 (1H, m, Ar‐H), 8.55 (1H, s, Ar‐H). ^13^C NMR (100 MHz, CDCl_3_) δ_C_: 28.63, 51.80, 55.60, 55.93, 55.96, 56.03, 56.19, 56.62, 67.84, 98.40, 98.55, 100.82, 107.65, 109.44, 111.42, 125.02, 125.84, 126.29, 126.62, 126.92, 127.70, 128.43, 128.79, 130.92, 133.44, 133.86, 137.22, 147.34, 147.70, 153.31, 157.89, 158.86, 161.13, and 163.92. HPLC acquisition time = 10 min., with a gradient consisting of ACN and water containing 0.1% diethylamine, with a linear increase of ACN from 10% to 100%, tR = 3.82 min. HRMS: m/z calcd for C_34_H_34_N_2_O_5_ 551.6628 [M + H^+^], found 551.2545.

**4‐[2‐(6,7‐Dimethoxy‐3,4‐dihydroisoquinolin‐2(1*H*)‐yl)ethoxy]−5,7‐dimethoxy‐2‐phenylquinoline** (**10**). General procedure (**E**) starting from **57** and using 6,7‐dimethoxytetrahydroisoquinoline, time = 5 h. After purification by flash column chromatography (CH_2_Cl_2_/MeOH 99:1), compound **10** was obtained as a white solid in 35% yield. ^1^H NMR (400 MHz, CDCl_3_) δ: 2.83–2.84 (2H, m, tetrahydroisoquinoline CH_2_), 2.88–2.95 (2H, m, tetrahydroisoquinoline CH_2_), 3.11 (2H, t, *J* = 5.0 Hz, CH_2_N), 3.76 (5H, s, OCH_3_ and tetrahydroisoquinoline CH_2_), 3.78 (3H, s, OCH_3_), 3.91 (3H, s, OCH_3_), 3.92 (3H, s, OCH_3_), 4.41 (2H, t, *J* = 5.0 Hz, OCH_2_), 6.41 (1H, d, *J* = 2.3 Hz, Ar‐H), 6.45 (1H, s, tetrahydroisoquinoline H), 6.54 (1H, s, tetrahydroisoquinoline H), 7.01 (1H, s, Ar‐H), 7.04 (1H, s, Ar‐H), 7.35–7.44 (3H, m, Ar‐H), 7.99 (2H, d, *J* = 8.4 Hz, Ar‐H). ^13^C NMR (100 MHz, CDCl_3_) δ_C_: 28.54, 51.73, 55.58, 55.93, 55.96, 56.01, 56.11, 56.55, 67.75, 98.27, 98.50, 100.81, 107.59, 109.43, 111.42, 125.79, 127.45, 128.73, 129.27, 139.97, 147.36, 147.72, 153.22, 157.85, 159.10, 161.08, and 163.85 ppm. HPLC acquisition time = 15 min. with a gradient consisting of ACN and water containing 0.1% diethylamine, with a linear increase of ACN from 20% to 100%, tR = 9.15 min. HRMS: m/z calcd for C_30_H_32_N_2_O_5_ 501.2389 [M + H^+^], found 501.2389.

**4‐{4‐[2‐(6,7‐Dimethoxy‐3,4‐dihydroisoquinolin‐2(1*H*)‐yl)ethoxy]−5,7‐dimethoxyquinolin‐2‐yl}phenol** (**11**). To a solution of **19** (1.25 g, 2.06 mmol) in EtOH (20 mL) and DMF (1 mL), Pd/C (0.125 g, 10% w/w) and H_2_ were bubbled into the reaction mixture. After 1 h, the reaction was filtered on celite and the solvent was removed under reduced pressure. After purification by flash column chromatography (CHCl_3_/MeOH 95:5), compound **11** was obtained as a yellow solid in 60% yield. ^1^H NMR (400 MHz, CDCl_3_) δ: 2.90–2.92 (2H, m, tetrahydroisoquinoline CH_2_), 3.00 −3.04 (2H, m, tetrahydroisoquinoline CH_2_), 3.18 (2H, t, *J* = 5.0 Hz, CH_2_N), 3.85 (2H, s, tetrahydroisoquinoline CH_2_), 3.86 (6H, s, OCH_3_ x2), 3.92 (3H, s, OCH_3_), 3.95 (3H, s, OCH_3_), 4.44 (2H, t, *J* = 5.1 Hz, OCH_2_), 6.48 (1H, d, *J* = 1.7 Hz, Ar‐H), 6.55 (1H, s, tetrahydroisoquinoline H), 6.63 (1H, s, tetrahydroisoquinoline H), 6.80 (2H, d, *J* = 8.4 Hz, Ar‐H), 6.93 (1H, s, Ar‐H), 7.10 (1H, d, *J* = 1.6 Hz, Ar‐H), 8.02 (2H, d, *J* = 8.6 Hz, Ar‐H), 9.80 (1H, s, OH). ^13^C NMR (100 MHz, CDCl_3_) δ_C_: 27.38, 28.52, 51.88, 55.62, 56.02, 56.31, 56.64, 67.78, 98.23, 100.26, 107.32, 109.44, 111.44, 116.03, 121.75, 125.83, 126.11, 129.08, 131.67, 140.07, 147.38, 147.74, 153.03, 157.93, 159.19, 161.18, and 163.86 ppm. HPLC acquisition time = 10 min with a gradient consisting of ACN and water containing 0.1% diethylamine, with a linear increase of ACN from 30% to 100%, tR = 3.42 min. HRMS: m/z calcd for C_30_H_32_N_2_O_6_ 517.2338 [M + H^+^], found 517.2336.

**4‐[2‐(6,7‐Dimethoxy‐3,4‐dihydroisoquinolin‐2(1*H*)‐yl)ethoxy]−5,7‐dimethoxy‐2‐(2‐propoxyphenyl)quinolone** (**12**). General procedure (**E**): starting from compound **58** and using 6,7‐dimethoxy‐1,2,3,4‐tetrahydroisoquinoline hydrochloride, time = 12 h. After flash column chromatography eluted with CHCl_3_ 100%, compound **12** was obtained as a white solid in 53% yield. ^1^H NMR (400 MHz, CDCl_3_) δ: 0.87 (3H, t, *J* = 7.5 Hz, OCH_2_CH_2_ *CH*_3_), 1.74–1.89 (2H, m, OCH_2_ *CH*_2_CH_3_), 2.85–2.86 (2H, m, tetrahydroisoquinoline CH_2_), 2.95–2.96 (2H, m, tetrahydroisoquinoline CH_2_), 3.13–3.14 (2H, m, CH_2_N), 3.79 (1H, s, tetrahydroisoquinoline CH_2_), 3.82 (3H, s, OCH_3_), 3.84 (3H, s, OCH_3_), 3.93 (3H, s, OCH_3_), 3.94 (3H, s, OCH_3_), 3.94–3.99 (2H, m, O*CH*_2_CH_2_CH_3_), 4.37–4.38 (2H, m, OCH_2_), 6.50 (1H, s, tetrahydroisoquinoline H), 6.51 (1H, s, tetrahydroisoquinoline H), 6.61 (1H, s, H3), 6.99 (1H, d, *J* = 8.2 Hz, aromatic H), 7.07–7.09 (2H, m, aromatic H), 7.35–7.39 (1H, m, aromatic H), 7.86 (1H, d, *J* = 7.1 Hz, aromatic H). ^13^C NMR (101 MHz, CDCl_3_) δ: 10.81, 22.78, 28.71, 51.60, 55.53, 55.92, 55.94, 55.99, 56.16, 56.65, 67.63, 70.23, 98.30, 100.83, 102.80, 107.48, 109.44, 111.43, 112.69, 121.10, 125.98, 126.54, 129.98, 130.09, 131.24, 147.27, 147.60, 153.02, 156.67, 157.77, 158.38, 160.64, and 162.37. HPLC acquisition time = 10 min. with a gradient consisting of ACN and water containing 0.1% diethylamine, with a linear increase of ACN from 50% to 100%, tR = 6.77 min. HRMS: m/z calcd for C_33_H_38_N_2_O_6_ 559.2808 [M + H^+^], found 559.2808.

**4‐[2‐(6,7‐Dimethoxy‐3,4‐dihydroisoquinolin‐2(1*H*)‐yl)ethoxy]−5,7‐dimethoxy‐2‐(4‐methoxyphenyl)quinoline** (**13**). General procedure (**E**) starting from compound **59** and using 6,7‐dimethoxytetraidroisoquinoline, time = 6 h. After flash column chromatography (CHCl_3_/MeOH 99:1) compound **13** was obtained as white solid in 30% yield. ^1^H NMR (400 MHz, CDCl_3_) δ: 2.90–2.91 2.98−2.99 (2H, m, tetrahydroisoquinoline CH_2_), 3.18 (2H, t, *J* = 5.2 Hz, CH_2_N), 3.83 (2H, s, tetrahydroisoquinoline CH_2_), 3.85 (3H, s, OCH_3_), 3.87 (3H, s, OCH_3_), 3.90 (3H, s, OCH_3_), 3.96 (3H, s, OCH_3_), 3.97 (3H, s, OCH_3_), 4.47 (2H, t, *J* = 5.2 Hz, OCH_2_), 6.49 (1H, s, Ar‐H), 6.55 (1H, s, tetrahydroisoquinoline H), 6.64 (1H, s, tetrahydroisoquinoline H), 7.03 (2H, d, *J* = 8.6 Hz, Ar‐H), 7.07–7.08 (2H, m, Ar‐H), 8.06 (2H, d, *J* = 8.7 Hz, Ar‐H). ^13^C NMR (100 MHz, CDCl_3_) δ_C_: 28.85, 51.92, 55.49, 55.63, 56.01, 56.06, 56.37, 56.76, 65.98, 67.96, 97.74, 98.21, 100.76, 107.41, 109.45, 111.43, 114.14, 126.01, 126.54, 128.81, 132.61, 147.31, 147.65, 153.34, 157.95, 158.70, 160.79, 161.05, and 163.85 ppm. HPLC acquisition time = 10 min with a gradient consisting of ACN and water containing 0.1% diethylamine, with a linear increase of ACN from 20% to 100%, tR = 3.27 min. HRMS: m/z calcd for C_31_H_34_N_2_O_6_ 531.2495 [M + H^+^], found 531.2491.

**2‐(4‐Butoxyphenyl)‐4‐[2‐(6,7‐dimethoxy‐3,4‐dihydroisoquinolin‐2(1*H*)‐yl)ethoxy]−5,7‐dimethoxyquinoline** (**14**). General procedure (**E**) starting from **60** and using 6,7‐dimethoxytetraidroisoquinoline, time = 5 h. After purification by flash column chromatography (CHCl_3_/MeOH 99:1), compound **14** was obtained as a yellow solid in 38% yield. ^1^H NMR (400 MHz, CDCl_3_) δ: 1.02 (3H, t, *J* = 7.3 Hz, OCH_2_CH_2_CH_2_ *CH*_3_), 1.51–1.56 (2H, m, OCH_2_CH_2_ *CH*_2_CH_3_), 1.81–1.84 (2H, m, OCH_2_ *CH*_2_CH_2_CH_3_), 2.90–2.91 (2H, m, tetrahydroisoquinoline CH_2_), 2.99–3.02 (2H, m, tetrahydroisoquinoline CH_2_), 3.18 (2H, t, *J* = 5.3 Hz, CH_2_N), 3.83 (2H, s, tetrahydroisoquinoline CH_2_), 3.85 (3H, s, OCH_3_), 3.87 (3H, s, OCH_3_), 3.96 (3H, s, OCH_3_), 3.97 (3H, s, OCH_3_), 4.05 (2H, t, *J* = 6.5 Hz, OCH_2_CH_2_ *CH*_2_CH_3_), 4.47 (2H, t, *J* = 5.3 Hz, OCH_2_), 6.49 (1H, d, *J* = 1.9 Hz, Ar‐H), 6.55 (1H, s, tetrahydroisoquinoline H), 6.64 (1H, s, tetrahydroisoquinoline H), 7.02 (2H, d, *J* = 8.7 Hz, Ar‐H), 7.07 (1H, s, Ar‐H), 7.08 (1H, d, *J* = 1.9 Hz, Ar‐H), 8.04 (2H, d, *J* = 8.7 Hz, Ar‐H). ^13^C NMR (100 MHz, CDCl_3_) δ_C_: 14.01, 15.40, 19.36, 28.85, 31.40, 51.92, 55.64, 56.01,56.38, 56.76, 65.98, 67.89, 97.72, 98.17, 100.74, 107.37, 109.43, 111.40, 114.71, 125.99, 126.53, 128.76, 132.33, 147.30, 147.64, 153.33, 157.93, 158.77, 160.40, 161.03, and 163.82 ppm. HPLC acquisition time = 10 min. with a gradient consisting of ACN and water containing 0.1% diethylamine with a linear increase of ACN from 10% to 100%, tR = 4.02 min. HRMS: m/z calcd for C_34_H_40_N_2_O_6_ 573.2964 [M + H^+^], found 573.2961.

**4‐[2‐(6,7‐Dimethoxy‐3,4‐dihydroisoquinolin‐2(1*H*)‐yl)ethoxy]‐2‐(4‐isopropoxyphenyl)−5,7‐dimethoxyquinoline** (**15**). General procedure (**H**) using 2‐iodopropane and K_2_CO_3_, time = 3 h. After flash column chromatography (CHCl_3_/MeOH 99:1), compound **15** was obtained as a solid in 20% yield. ^1^H NMR (400 MHz, CDCl_3_) δ: 1.37 (6H, d, *J* = 6.1 Hz, CH_3_), 2.88 (2H, t, *J* = 5.6 Hz, tetrahydroisoquinoline CH_2_), 2.98 (6.1 Hz, t, *J* = tetrahydroisoquinoline CH_2_), 3.15 (2H, t, *J* = 5.6 Hz, CH_2_N), 3.81 (2H, s, tetrahydroisoquinoline CH_2_), 3.83 (3H, s, OCH_3_), 3.85 (3H, s, OCH_3_), 3.93 (3H, s, OCH_3_), 3.94 (3H, s, OCH_3_), 4.44 (2H, t, *J* = 5.6 Hz, OCH_2_), 4.59–4.65 (1H, m, CH), 6.47 (1H, d, *J* = 2.3 Hz, Ar‐H), 6.52 (1H, s, tetrahydroisoquinoline H), 6.61 (1H, s, tetrahydroisoquinoline H), 3.85 (2H, d, *J* = 8.8 Hz, Ar‐H), 7.04 (1H, s, Ar‐H), 7.07 (1H, d, *J* = 2.3 Hz, Ar‐H), 8.00 (2H, d, *J* = 8.8 Hz, Ar‐H). ^13^C NMR (100 MHz, CDCl_3_) δ_C_: 22.03, 28.75, 51.80, 55.53, 55.92, 55.95, 55.98, 56.26, 56.67, 67.84, 69.97, 97.68, 98.14, 100.72, 107.33, 109.46, 111.43, 115.94, 125.96, 126.49, 128.72, 132.27, 147.29, 147.63, 153.26, 157.87, 158.71, 159.08, 160.96, and 163.75 ppm. HPLC acquisition time = 15 min. with a gradient consisting of ACN and water containing 0.1% of diethylamine with a linear increase of ACN from 10% to 100%, tR = 3.60 min. HRMS: m/z calcd for C_33_H_38_N_2_O_6_ 559.2808 [M +&H^+^], found 559.2803.

**4‐[2‐(6,7‐Dimethoxy‐3,4‐dihydroisoquinolin‐2(1*H*)‐yl)ethoxy]−5,7‐dimethoxy‐2‐[4‐(pentyloxy)phenyl]quinoline** (**16**). General procedure (**H**), starting from **11**, using Cs_2_CO_3_ and pentyl 4‐methylbenzenesulfonate, time = 1 h. After purification by flash column chromatography eluted with CHCl_3_/MeOH 99:1, compound **16** was obtained as a white solid in 29% yield. ^1^H NMR (400 MHz, CDCl_3_) δ: 0.87 (3H, t, *J* = 6.7 Hz, pentyloxy CH_3_), 1.34–1.39 (4H, m, pentyloxy CH_2_), 1.61–1.68 (2H, m, pentyloxy CH_2_), 3.62–3.64 (2H, m, tetrahydroisoquinoline CH_2_), 2.91–2.93 (2H, m, tetrahydroisoquinoline CH_2_), 3.09–3.11 (2H, m, CH_2_N), 3.76–3.78 (8H, m, OCH_3_, OCH_3_ and trahydroisoquinoline CH_2_), 3.86 (3H, s, OCH_3_), 3.88 (3H, s, OCH_3_), 3.95 (2H, t, *J* = 5.7 Hz, pentyloxy OCH_2_), 4.37–4.41 (2H, m, OCH_2_), 6.40 (1H, s, H6), 6.45 (1H, s, tetrahydroisoquinoline H), 6.54 (1H, s, tetrahydroisoquinoline H), 6.92 (2H, d, *J* = 7.9 Hz, H3’ and H5’), 6.97–7.02 (2H, m, H8 and H3), 7.96 (2H, d, *J* = 7.4 Hz, H2’ and H6’). ^13^C NMR (100 MHz, CDCl_3_) δ: 14.05, 22.50, 28.21, 28.59, 28.96, 51.74, 55.58, 55.92, 55.95, 55.99, 56.14, 56.58, 67.73, 68.14, 97.68, 98.21, 100.56, 107.28, 109.43, 111.41, 114.66, 124.83, 125.81, 128.74, 132.15, 147.34, 147.69, 153.06, 157.86, 158.59, 160.41, 161.07, and 163.80. HPLC acquisition time = 10 min. with a gradient consisting of ACN and water containing 0.1% formic acid, with a linear increase of ACN from 20% to 100%, tR = 4.69 min. HRMS: m/z calcd for C_35_H_42_N_2_O_6_ 587.3121 [M + H^+^], found 587.3120.

**2‐[4‐(Octyloxy)phenyl[‐4‐2‐] (6,7‐dimethoxy‐3,4‐dihydroisoquinolin‐2(1*H*)‐yl)ethoxy]−5,7‐dimethoxyquinoline** (**17**). General procedure (**H**), starting from **11**, using Cs_2_CO_3_ and using octyl 4‐methylbenzenesulfonate, time = 12 h. After purification by flash column chromatography eluted with CH_2_Cl_2_/MeOH 99:1, compound **17** was obtained as a solid in 41% yield. ^1^H NMR (400 MHz, CDCl_3_) δ: 0.81 (3H, t, *J* = 6.6 Hz, octyloxy CH_3_), 1.18–1.27 (8H, m, octyloxy CH_2_), 1.36–1.41 (2H, m, octyloxy CH_2_), 1.71–1.76 (2H, m, octyloxy CH_2_), 2.81–2.83 (2H, m, tetrahydroisoquinoline CH_2_), 2.91–2.93 (2H, m, tetrahydroisoquinoline CH_2_), 3.09–3.10 (2H, m, CH_2_N), 3.75–3.78 (8H, m, OCH_3_, OCH_3_ and tetrahydroisoquinoline CH_2_), 3.86 (3H, s, OCH_3_), 3.88 (3H, s, OCH_3_), 3.95 (2H, t, *J* = 6.4 Hz, octyloxy OCH_2_), 4.39 (2H, m, OCH_2_), 6.40 (1H, s, H6), 6.45 (1H, s, tetrahydroisoquinoline H), 6.54 (1H, s, tetrahydroisoquinoline H), 6.93 (2H, d, *J* = 8.5 Hz, H3’ and H5’), 6.97 (1H, s, H3), 7.02 (1H, s, H8), 7.96 (2H, d, *J* = 8.4 Hz, H2’ and H6’). ^13^C NMR (100 MHz, CDCl_3_) δ: 14.12, 22.67, 26.06, 28.60, 29.26, 29.39, 29.71, 31.83, 51.75, 55.57, 55.93, 55.95, 55.99, 56.15, 56.59, 67.75, 68.16, 97.67, 98.21, 100.61, 107.29, 109.44, 111.42, 114.66, 124.84, 125.85, 128.73, 147.34, 147.69, 157.87, 158.61, 160.41, 161.06, and 163.79 ppm. HPLC acquisition time = 10 min. with a gradient consisting of ACN and water containing 0.1% formic acid, with a linear increase of ACN from 20& to 100%, tR = 5.51 min. HRMS: m/z calcd for C_38_H_48_N_2_O_6_ 629.35990 [M + H^+^], found 629.3590.

**2‐(Cyclohexyloxy)‐4‐[2‐(6,7‐dimethoxy‐3,4‐dihydroisoquinolin‐2(1*H*)‐yl)ethoxy]−5,7‐dimethoxyquinoline** (**18**). General procedure (**H**), starting from **11**, using Cs_2_CO_3_ and cyclohexyl p‐toluenesulfonate, time = 5 h. After purification by flash column chromatography eluted with CHCl_3_/acetone 95/5, compound **18** was obtained as a solid in 32% yield. ^1^H NMR (600 MHz, CDCl_3_) δ: 1 1.36–1.45 (4H, m, cyclohexyl CH_2_), 1.46–1.54 (2H, m, cyclohexyl CH_2_), 1.74–1.76 (2H, m, cyclohexyl CH_2_), 1.93–1.96 (2H, m, cyclohexyl CH_2_), 2.81 (2H, t, *J* = 5.6 Hz, tetrahydroisoquinoline CH_2_), 2.91 (2H, t, *J* = 5.8 Hz, tetrahydroisoquinoline CH_2_), 3.08 (2H, t, *J* = 5.5 Hz, CH_2_N), 3.72 (2H, s, tetrahydroisoquinoline CH_2_), 3.74 (3H, s, OCH_3_), 3.78 (3H, s, OCH_3_), 3.86 (3H, s, OCH_3_), 3.87 (3H, s, OCH_3_), 4.24–4.26 (1H, m, cyclohexyl CH), 4.28 (2H, t, *J* = 5.0 Hz, OCH_2_), 9.59 (1H, d, *J* = 2.2 Hz, H6), 6.45 (1H, s, tetrahydroisoquinoline H), 6.54 (1H, s, tetrahydroisoquinoline H), 6.93 (2H, d, *J* = 8.7 Hz, H3’ and H5’), 6.97 (1H, s, H3), 6.99 (1H, s, H8), 7.93 (2H, d, *J* = 8.7 Hz, H2’ and H6’). ^13^C NMR (100 MHz, CDCl_3_) δ: 22.71, 24.61, 27.70, 28.69, 30.73, 50.77, 52.41, 54.51, 54.90, 54.96, 55.23, 55.63, 66.80, 74.41, 96.66, 97.12, 99.69, 106.30, 108.43, 110.40, 115.09, 124.92, 125.44, 127.68, 131.24, 146.27, 146.61, 152.24, 156.85, 157.72, 157.98, 159.95, and 162.72. HPLC acquisition time = 10 min with a gradient consisting of ACN and water containing 0.1% diethylamine, with a linear increase of ACN from 20% to 100%, tR = 4.58 min. HRMS: m/z calcd for C_36_H_42_N_2_O_6_ 599.3121 [M + H^+^], found 599.3122.

**2‐[4‐(Benzyloxy)phenyl[‐4‐2‐] (6,7‐dimethoxy‐3,4‐dihydroisoquinolin‐2(1*H*)‐yl)ethoxy]−5,7‐dimethoxyquinoline** (**19**). General procedure (**E**) starting from **81** and using 6,7‐dimethoxytetrahydroisoquinoline, time = 5 h. After purification by flash column chromatography (CHCl_3_/MeOH 99:1), compound **19** was obtained as a solid in 21% yield. ^1^H NMR (400 MHz, CDCl_3_) δ: 2.89–2.91 (2H, m, tetrahydroisoquinoline CH_2_), 2.97–2.99 (2H, m, tetrahydroisoquinoline CH_2_), 3.14–3.18 (2H, m, CH_2_N), 3.83 (2H, s, tetrahydroisoquinoline CH_2_), 3.85 (3H, s, OCH_3_), 3.98 (3H, s, OCH_3_), 3.96 (3H, s, OCH_3_), 3.96 (3H, s, OCH_3_), 4.46 (2H, t, *J* = 6.2 Hz, OCH_2_), 5.16 (2H, s, benzylic CH_2_), 6.49 (1H, s, Ar‐H), 6.54 (1H, s, tetrahydroisoquinoline H), 6.63 (1H, s, tetrahydroisoquinoline H), 7.06–7.11 (4H, m, Ar‐H), 7.34–7.50 (5H, m, Ar‐H), 8.05 (2H, d, *J* = 8.3 Hz, Ar‐H). ^13^C NMR (100 MHz, CDCl_3_) δ_C_: 28.83, 29.81, 36.62, 51.91, 55.65, 56.01, 56.36, 56.74, 67.93, 70.12, 97.77, 98.22, 100.72, 107.40, 109.43, 111.39, 115.11, 125.99, 126.50, 127.61, 128.14, 128.73, 128.83, 132.85, 136.87, 147.30, 147.64, 153.32, 157.93, 158.68, 159.93, 161.06, and 163.85 ppm. HPLC acquisition time = 10 min with a gradient consisting of ACN and water containing 0.1% diethylamine, with a linear increase of ACN from 10% to 100%, tR = 3.58 min. HRMS: m/z calcd for C_37_H_38_N_2_O_6_ 607.2808 [M + H^+^], found 607.2808.

**4‐[2‐(6,7‐Dimethoxy‐3,4‐dihydroisoquinolin‐2(1*H*)‐yl)ethoxy]−5,7‐dimethoxy‐2‐[4‐(2‐phenylethoxy)phenyl]quinoline** (**20**). General procedure (**D**) starting from compound **11** (0.150g, 0.29 mmol) and using 2‐chloroethyl benzol (0.114g, 0.87 mmol), time = 12 h. After purification by flash column chromatography eluted with CHCl_3_/MeOH 99:1, compound **20** was obtained as a solid in 26% yield. ^1^H NMR (400 MHz, CDCl_3_) δ: 4.02–2.92 (2H, m, tetrahydroisoquinoline CH_2_), 2.99–3.01 (2H, m, tetrahydroisoquinoline CH_2_), 3.15–3.18 (4H, m, CH_2_N and CH_2_), 3.79 (2H, s, tetrahydroisoquinoline CH_2_), 3.85 (3H, s, OCH_3_), 3.87 (3H, s, OCH_3_), 3.96 (3H, s, OCH_3_), 3.97 (3H, s, OCH_3_), 4.21 (2H, t, *J* = 7.2 Hz, OCH_2_), 4.47 (2H, t, *J* = 5.3 Hz, OCH_2_), 6.49 (1H, d, *J* = 2.1 Hz, H6), 6.55 (1H, s, tetrahydroisoquinoline H), 6.64 (1H, s, tetrahydroisoquinoline H), 7.03 (2H, d, *J* = 8.7 Hz, H3’ and H5’), 7.07 (1H, s, H3), 7.09 (1H, s, H8), 7.29 (1H, s, aromatic H), 7.33–7.39 (4H, m, aromatic H), 8.05 (2H, d, *J* = 8.7 Hz, H2’ and H6’). ^13^C NMR (100 MHz, CDCl_3_) δ: 28.72, 35.77, 51.80, 55.54, 55.92, 55.95, 55.99, 55.25, 56.25, 56.65, 67.84, 68.80, 97.67, 98.18, 100.69, 107.35, 109.45, 111.42, 114.70, 125.93, 126.57, 128.54, 128.72, 129.04, 138.11, 147.29, 147.63, 157.87, 158.59, 159.95, and160.99. HPLC acquisition time = 15 min. with a gradient consisting of ACN and water containing 0.1% diethylamine, with a linear increase of ACN from 20% to 100%, tR = 10.74 min. HRMS: m/z calcd for C_38_H_40_N_2_O_6_ 621.2964 [M + H^+^], found 621.2964.

**4‐[2‐(6,7‐Dimethoxy‐3,4‐dihydroisoquinolin‐2(1*H*)‐yl)ethoxy]−5,7‐dimethoxy‐2‐[4‐(3‐morpholin‐4‐ylpropoxy)phenyl]quinoline** (**21**). General procedure (**H**) using 3‐(chloropropyl)morpholine and K_2_CO_3_, time = 4 h. After flash column chromatography (CHCl_3_/MeOH 97:3) compound **21** was obtained as white solid in 22% yield. ^1^H NMR (400 MHz, CDCl_3_) δ: 1.90–1.97 (2H, m, CH_2_), 2.42–2.43 (4H, m, morpholine CH_2_), 2.49 (2H, t, *J* = 7.1 Hz, CH_2_N), 2.79–2.82 (2H, m, tetrahydroisoquinoline CH_2_), 2.91 (2H, t, *J* = 6.02 Hz, tetrahydroisoquinoline CH_2_), 3.09 (2H, t, *J* = 5.6 Hz, CH_2_N), 3.66 (4H, t, *J* = 5.0 Hz, morpholine CH_2_), 3.74 (2H, s, tetrahydroisoquinoline CH_2_), 3.76 (3H, s, OCH_3_), 3.78 (3H, s, OCH_3_), 3.86 (3H, s, OCH_3_), 3.88 (3H, s, OCH_3_), 4.03 (2H, t, *J* = 6.3 Hz, OCH_2_), 4.37 (2H, t, *J* = 5.6 Hz, OCH_2_), 6.40 (1H, d, *J* = 2.3 Hz, Ar‐H), 6.45 (1H, s, tetrahydroisoquinoline H), 6.54 (1H, s, tetrahydroisoquinoline H), 6.93 (1H, d, *J* = 8.8 Hz, Ar‐H), 6.97 (1H, s, Ar‐H), 6.99 (1H, d, *J* = 2.3 Hz, Ar‐H), 7.95 (2H, d, *J* = 8.8 Hz, Ar‐H). ^13^C NMR (101 MHz, CDCl_3_) δ_C_: 25.41, 27.68, 50.76, 52.74, 54.49, 54.50, 54.88, 54.91, 54.94, 55.22, 55.62, 65.19, 65.97, 66.79, 96.60, 97.13, 99.65, 106.31, 108.42, 110.39, 113.60, 124.90, 125.41, 127.66, 131.42, 146.24, 146.59, 152.22, 156.83, 157.56, 159.11, 159.95, and 162.74 ppm. HPLC acquisition time = 10 min with a gradient consisting of ACN and water containing 0.1% diethylamine, with a linear increase of ACN from 50% to 100%, tR = 5.19 min. HRMS: m/z calcd for C_37_H_45_N_3_O_7_ 644.3336 [M + H^+^], found 644.3331.

**4‐{4‐[2‐(6,7‐Dimethoxy‐3,4‐dihydroisoquinolin‐2(1*H*)‐yl)ethoxy]−5,7‐dimethoxyquinolin‐2‐yl}phenyl methanesulfonate** (**22**). General procedure (**H**) using methanesulfonyl chloride and K_2_CO_3_, time = 6 h. after purification by flash column chromatography (CHCl_3_/MeOH 99:1), compound **22** was obtained as a solid in 63% yield. ^1^H NMR (400 MHz, DMSO‐*d*_6_) δ: 2.76 (2H, t, *J* = 5.5 Hz, tetrahydroisoquinoline CH_2_), 2.88 (2H, t, *J* = 6.0 Hz, tetrahydroisoquinoline CH_2_), 3.02 (2H, t, *J* = 5.2 Hz, CH_2_N), 3.45 (3H, s, CH_3_), 3.70 (3H, s, OCH_3_), 3.71 (3H, s, OCH_3_), 3.72 (2H, s, tetrahydroisoquinoline CH_2_), 3.89 (3H, s, OCH_3_), 3.91 (3H, s, OCH_3_), 4.47 (2H, t, *J* = 5.3 Hz, OCH_2_), 6.59 (1H, d, *J* = 2.4 Hz, Ar‐H), 6.60 (1H, s, tetrahydroisoquinoline H), 6.68 (1H, s, tetrahydroisoquinoline H), 6.99 (1H, d, *J* = 2.3 Hz, Ar‐H), 7.41 (1H, s, Ar‐H), 7.49 (2H, d, *J* = 8.8 Hz, Ar‐H), 8.37 (2H, d, *J* = 8.8 Hz, Ar‐H). ^13^C NMR (100 MHz, CDCl_3_) δ_C_: 28.81, 29.78, 37.47, 51.90, 55.66, 56.01, 56.11, 56.36, 56.71, 68.15, 98.13, 98.78, 100.79, 107.77, 109.48, 111.48, 122.29, 125.99, 126.47, 129.26, 139.50, 147.36, 147.71, 150.09, 153.30, 157.63, 157.99, 161.32, and 164.22 ppm. HPLC acquisition time = 10 min with a gradient consisting of ACN and water containing 0.1% diethylamine, with a linear increase of ACN from 30% to 100%, tR = 3.99 min. HRMS: m/z calcd for C_31_H_34_N_2_O_8_S 595.2114 [M + H^+^], found 595.2109.

**2‐(4‐Butylphenyl)‐4‐[2‐(6,7‐dimethoxy‐3,4‐dihydroisoquinolin‐2(1*H*)‐yl)ethoxy]−5,7‐dimethoxyquinoline hydrochloride** (**23**). General procedure (**E**) starting from compound **61** and using 6,7‐dimethoxytetrahydroisoquinoline, time = 6 h. After purification by flash column chromatography (CHCl_3_/MeOH 97:3), the obtained solid was solubilized in Et_2_O and HCl was bubbled for 10 min until the formation of a yellow precipitate recovered by filtration obtaining compound **23** in 29% yield. ^1^H NMR (400 MHz, DMSO‐*d*_6_) δ: 0.99 (3H, t, *J* = 7.3 Hz, butyl CH_3_), 1.36–1.45 (2H, m, butyl CH_2_), 1.65–1.72 (2H, m, butyl CH_2_), 2.79 (2H, t, *J* = 7.5 Hz, butyl CH_2_), 2.99–3.00 (1H, m, tetrahydroisoquinoline H), 3.34–3.43 (1H, m, tetrahydroisoquinoline H), 3.78 (3H, s, OCH_3_), 3.80, 3.95–3.96 (2H, m, CH_2_N), 4.02 (3H, s, OCH_3_), 4.08 (3H, s, OCH_3_), 4.52–4.63 (2H, m, tetrahydroisoquinoline CH_2_), 5.17–5.18 (2H, m, OCH_2_), 6.76 (1H, s, tetrahydroisoquinoline H), 6.85 (1H, s, tetrahydroisoquinoline H), 6.92 (1H, s, Ar‐H), 7.57 (2H, d, *J* = 8.0 Hz, Ar‐H), 7.61 (1H, s, Ar‐H), 7.79 (1H, s, Ar‐H), 8.24 (2H, d, *J* = 8.1 Hz, Ar‐H). ^13^C NMR (400 MHz, DMSO‐*d*_6_) δ_C_: 14.23, 22.20, 25.20, 33.29, 35.13, 49.79, 52.62, 54.33, 56.00, 56.07, 56.64, 57.28, 66.44, 100.22, 100.78, 106.79, 109.79, 112.02, 120.54, 123.70, 129.53, 148.15, 148.80, 155.56, 158.95, and 164.51 ppm*.* HPLC acquisition time = 15 min. with a gradient consisting of ACN and water containing 0.1% diethylamine, with a linear increase of ACN from 50% to 100%, tR = 10.13 min. HRMS: m/z calcd for C_34_H_40_N_2_O_5_ 557.3008 [M + H^+^], found 557.3008.

**4‐[2‐(6,7‐Dimethoxy‐3,4‐dihydroisoquinolin‐2(1*H*)‐yl)ethoxy]−5,7‐dimethyl‐2‐(4‐propoxyphenyl)quinoline** (**24**). General procedure (**E**) starting from compound **82** and using 6,7‐dimethoxytetrahydroisoquinoline, time = 7 h. After purification by flash column chromatography (CHCl_3_/MeOH 98:2) compound **24** was obtained as brownish solid in 37% yield. ^1^H NMR (400 MHz, CDCl_3_) δ: (3H, t, *J* = 7.4 Hz, OCH_2_CH_2_ *CH*_3_), 1.73–1.82 (2H, m, OCH_2_ *CH*_2_CH_3_), 2.39 (3H, s, CH_3_), 2.79–2.83 (7H, m, CH_3_, tetrahydroisoquinoline CH_2_), 3.07 (2H, t, *J* = 5.9 Hz, CH_2_N), 3.67 (2H, s, tetrahydroisoquinoline CH_2_), 3.76 (3H, s, OCH_3_), 3.78 (3H, s, OCH_3_), 3.92 (2H, t, *J* = 7.4 Hz, O*CH*_2_CH_2_CH_3_), 4.35 (2H, t, *J* = 5.9 Hz, OCH_2_), 6.46 (1H, s, tetrahydroisoquinoline H), 6.54 (1H, s, tetrahydroisoquinoline H), 6.93 (2H, d, *J* = 8.7 Hz, Ar‐H), 6.96 (1H, s, Ar‐H), 7.02 (1H, s, Ar‐H), 7.64 (1H, s, Ar‐H), 7.97 (2H, d, *J* = 8.7 Hz, Ar‐H). ^13^C NMR (101 MHz, CDCl_3_) δ_C_: 10.53, 21.42, 22.60, 24.68, 28.71, 51.75, 55.92, 55.96, 56.10, 56.70, 66.88, 69.63, 98.10, 109.443, 111.41, 114.63, 117.49, 125.92, 126.18, 126.81, 128.62, 130.10, 132.43, 139.25, 147.31, 147.66, 151.31, 157.59, 160.25, and 164.26 ppm. HPLC acquisition time = 10 min. with a gradient consisting of ACN and water containing 0.1% diethylamine, with a linear increase of ACN from 20% to 100%, tR = 4.36 min. HRMS: m/z calcd for C_33_H_38_N_2_O_4_ 527.2910 [M + H^+^], found 527.2910.

**4‐[2‐(6,7‐Dimethoxy‐3,4‐dihydroisoquinolin‐2(1*H*)‐yl)ethoxy]−5,8‐dimethoxy‐2‐(4‐propoxyphenyl)quinoline** (**25**). General procedure (**E**), starting from compound **83** using 6,7‐dimethoxytetraidrohysoquinoline hydrochloride, time = 5 h. After purification by flash column chromatography eluted with CHCl_3_/acetone 95:5, compound **25** was obtained as a white solid in 21% yield. ^1^H NMR (600 MHz, CDCl_3_) δ: 1.08 (3H, t, *J* = 7.4 Hz, OCH_2_CH_2_ *CH*_3_), 1.84–1.89 (2H, m, OCH_2_ *CH*_2_CH_3_), 2.91–2.92 (2H, m, tetrahydroisoquinoline CH_2_), 2.99–3.01 (2H, m, tetrahydroisoquinoline CH_2_), 3.20 (2H, t, *J* = 5.6 Hz, CH_2_N), 3.84 (2H, s, tetrahydroisoquinoline CH_2_), 3.86 (3H, s, OCH_3_), 3.87 (3H, s, OCH_3_), 3.94 (3H, s, OCH_3_), 4.01 (2H, t, *J* = 6.7 Hz, O*CH*_2_CH_2_CH_3_), 4.07 (3H, s, OCH_3_), 4.49 (2H, t, *J* = 5.6 OCH_2_), 6.56 (1H, s, tetrahydroisoquinoline H), 6.64 (1H, s, tetrahydroisoquinoline H), 6.78 (1H, d, *J* = 8.7 Hz, H7), 6.98 (1H, d, *J* = 8.7 Hz, H6), 7.03 (1H, d, *J* = 8.9 Hz, H3’ and H5’), 7.28 (1H, s, H3), 8.13 (2H, d, *J* = 8.9 Hz, H2’ and H5’). ^13^C NMR (100 MHz, CDCl_3_) δ: 10.64, 22.69, 28.83, 51.92, 55.99, 56.02, 56.36, 56.49, 56.74, 56.90, 67.95, 69.67, 99.64, 105.72, 108.27, 109.47, 111.41, 1113.19, 114.66, 126.01, 126.51, 128.93, 132.23, 142.96, 147.30, 147.65, 149.75, 150.45, 157.29, 160.46, and 163.76. HPLC acquisition time = 10 min. with a gradient consisting of ACN and water containing 0.1% diethylamine, with a linear increase of ACN from 50% to 100%, tR = 3.70 min. HRMS: m/z calcd for C_33_H_38_N_2_O_6_ 559.2808 [M + H^+^], found 559.2807.

**2‐(4‐Butoxyphenyl)‐4‐[2‐(6,7‐dimethoxy‐3,4‐dihydroisoquinolin‐2(1*H*)‐yl)ethoxy]−5,8‐dimethoxyquinoline** (**26**). General procedure (**E**), starting from compound **84** using 6,7‐dimethoxytetraidrohysoquinoline hydrochloride, time = 4 h. After purification by flash column chromatography eluted with CHCl_3_/MeOH 99/1, compound **26** was obtained as a white solid in 50% yield. ^1^H NMR (400 MHz, CDCl_3_) δ: 1.07 (3H, t, *J* = 6.8 Hz, butoxy CH_3_), 1.39–1.49 (2H, m, butoxy CH_2_), 1.69–1.76 (2H, m, butoxy CH_2_), 2.81–2.82 (2H, m, tetrahydroisoquinoline CH_2_), 2.88–2.92 (2H, m, tetrahydroisoquinoline CH_2_), 3.09 (2H, t, *J* = 5.3 Hz, tetrahydroisoquinoline CH_2_), 3.74–3.78 (8H, m, CH_2_N, OCH_3_ and OCH_3_), 3.84 (3H, s, OCH_3_), 3.91–3.96 (5H, m, OCH_3_ and butoxy CH_2_), 4.39 (2H, t, *J* = 5.3 Hz, OCH_2_), 6.46 (1H, s, tetrahydroisoquinoline CH), 6.54 (1H, s, tetrahdroisoquinoline CH), 6.69 (1H, d, *J* = 8.6 Hz, H6), 6.88–6.93 (3H, m, H7 and H3’ and H5’), 7.18 (1H, s, H3), 8.03 (2H, d, *J* = 8.4 Hz). ^13^C NMR (101 MHz, CDCl_3_) δ: 13.90, 19.26, 28.73, 31.32, 51.80, 55.92, 55.95, 56.25, 56.44, 56.65, 56.85, 67.80, 67.86, 99.59, 105.74, 108.31, 109.48, 111.43, 113.16, 114.59, 125.96, 126.45, 128.84, 132.18, 142.92, 147.28, 147.62, 149.73, 150.41, 157.20, 160.40, and 163.68. HPLC acquisition time = 12 min. with a gradient consisting of ACN and water containing 0.1% diethylamine, with a linear increase of ACN from 50% to 100%, tR = 8.47 min. HRMS: m/z calcd for C_34_H_40_N_2_O_6_ 573.2964 [M + H^+^], found 573.2963.

**4‐[2‐(6,7‐Dimethoxy‐3,4‐dihydroisoquinolin‐2(1*H*)‐yl)ethoxy]−5,8‐diethoxy‐2‐(4‐propoxyphenyl)quinoline** (**28**). General procedure (**E**), starting from compound **85** using 6,7‐dimethoxytetraidrohysoquinoline hydrochloride, time = 12 h. After flash column chromatography eluted with CHCl_3_ 100%, compound **28** was obtained as a white solid in 35% yield. ^1^H NMR (400 MHz, CDCl_3_) δ: 1.06 (3H, t, *J* = 7.4 Hz, OCH_2_CH_2_ *CH*_3_), 1.49 (3H, t, *J* = 6.9 Hz, OCH_2_ *CH*_3_), 1.61 (3H, t, *J* = 6.9 Hz, OCH_2_ *CH*_3_), 1.80 (2H, m, OCH_2_ *CH*_2_CH_3_), 2.88–2.89 (2H, m, tetrahydroisoquinoline CH_2_), 2.93–2.94 (2H, m, tetrahydroisoquinoline CH_2_), 3.18 (2H, t, *J* = 6.1 Hz, CH_2_N), 3.77 (2H, s, tetrahydroisoquinoline CH_2_), 3.83 (3H, s, OCH_3_), 3.85 (3H, s, OCH_3_), 3.99 (2H, t, *J* = 6.6 Hz, O*CH*_2_CH_2_CH_3_), 4.09 (2H, q, *J* = 6.9 Hz, O*CH*_2_CH_3_), 4.27 (2H, q, *J* = 6.9 Hz, O*CH*_2_CH_3_), 4.47 (2H, t, *J* = 6.1 Hz, OCH_2_), 6.53 (1H, s, tetrahydroisoquinoline H), 6.61 (1H, s, tetrahydroisoquinoline H), 6.73 (1H, d, *J* = 8.6 Hz, H6), 6.97 (1H, s, H3), 7.00 (2H, d, *J* = 8.8 Hz, H3’ and H5’), 7.23–7.25 (1H, m, H7), 8.15 (2H, d, *J* = 7.8 Hz, H2’ and H6’). ^13^C NMR (101 MHz, CDCl_3_) δ: 10.53, 15.05, 15.25, 22.59, 28.66, 51.79, 55.91, 55.95, 56.14, 56.63, 65.28, 65.38, 67.10, 69.58, 98.99, 107.06, 109.44, 110.90, 111.39, 113.34, 114.54, 125.89, 128.67, 132.08, 143.24, 147.29, 147.65, 149.02, 149.84, 156.62, 160.39, and 163.80. HPLC acquisition time = 10 min. with a gradient consisting of ACN and water containing 0.1% diethylamine, with a linear increase of ACN from 50% to 100%, tR = 8.91 min. HRMS: m/z calcd for C_35_H_42_N_2_O_6_ 587.3121 [M + H^+^], found 587.3120.

**4‐[2‐(6,7‐Dimethoxy‐3,4‐dihydroisoquinolin‐2(1*H*)‐yl)ethoxy]−5,6,7‐trimethoxy‐2‐(4‐propoxyphenyl)quinoline hydrochloride** (**29**). General procedure (**E**), starting from compound **87** [17] using 6,7‐dimethoxytetraidrohysoquinoline hydrochloride, time = 12 h. After purification by flash column chromatography eluted with CHCl_3_/MeOH 99:1, compound **29** was obtained as an oil, which was then solubilized in Et_2_O and HCl was bubbled into the solution, obtaining **29** as a yellow solid in 60% yield. ^1^H NMR (400 MHz, DMSO‐*d*_6_) δ: 1.08 (3H, t, *J* = 7.4 Hz, OCH_2_CH_2_ *CH*_3_), 1.81–1.90 (2H, m, OCH_2_ *CH*_2_CH_3_), 3.01–3.06 (1H, m, tetrahydroisoquinoline CH_2_), 3.54–3.57 (1H, m, tetrahydroisoquinoline CH), 3.75 (3H, s, OCH_3_), 3.78 (3H, s, OCH_3_), 3.93–3.97 (12H, m, OCH_3_, OCH_3_, CH_2_N, tetrahydroisoquinoline CH), 4.08 (3H, s, OCH_3_), 4.16 (2H, t, *J* = 6.5 Hz, O*CH*_2_CH_2_CH_3_), 4.79–4.52 (1H, m, tetrahydroisoquinoline CH), 4.64–4.68 (1H, m, tetrahydroisoquinoline CH), 5.17 (1H, bs, OCH_2_), 6.77 (1H, s, tetrahydroisoquinoline H), 6.90 (1H, s, tetrahydroisoquinoline H), 7.31 (2H, d, *J* = 8.7 Hz, H3’ and H5’), 7.67 (1H, s, H8), 8.00 (1H, s, H3), 8.32 (2H, d, *J* = 8.8 Hz, H2’ and H6’), 11.85 (1H, s, HCl). ^13^C NMR (101 MHz, DMSO‐*d*_6_) δ:10.81, 22.39, 25.00, 49.92, 52.51, 53.99, 55.99, 56.01, 57.08, 61.59, 62.80, 66.23, 70.01, 100.55, 109.79, 110.16, 112.03, 115.85, 120.37, 123.63, 131.36, 143.35, 148.16, 148.84, and149.65. HPLC acquisition time = 12 min. with a gradient consisting of ACN and water containing 0.1% diethylamine, with a linear increase of ACN from 50% to 100%, tR = 7.73 min. HRMS: m/z calcd for C_34_H_40_N_2_O_7_ 589.2914 [M + H^+^], found 589.2910.

**5,7‐Dimethoxy‐4‐propoxy‐2‐(4‐propoxyphenyl)quinoline** (**31**). General procedure (**D**) starting from **30** [23] and using 1‐bromo‐3‐chloropropane, after purification by flash column chromatography eluted with Cyclohexane/EtOAc 90:10 as a solid in 33% yield. ^1^H NMR (400 MHz, CDCl_3_) δ: 1.01 (3H, t, *J* = 7.4 Hz, OCH_2_CH_2_ *CH*_3_), 1.75–1.80 (2H, m, OCH_2_ *CH*_2_CH_3_), 2.27–2.30 (2H, m, CH_2_), 3.86 (3H, s, OCH_3_), 3.90 (3H, s, OCH_3_), 3.96 (2H, t, *J* = 6.4 Hz, CH_2_Cl), 4.01 (2H, t, *J* = 6.5 Hz, O*CH*_2_CH_2_CH_3_), 4.38 (2H, t, *J* = 5.6 Hz, OCH_2_), 6.53 (1H, d, *J* = 2.3 Hz, H6), 6.94 (1H, *J* = 2.0 Hz, H8), 7.06 (2H, d, *J* = 8.9 Hz, H3’ and H5’), 7.29 (1H, s, H3), and 8.22 (2H, d, *J* = 8.8 Hz, H2’ and H6’).

***N*‐(3,5‐dimethoxyphenyl)thiophene‐2‐carboxamide** (**34**). General procedure (**A**), starting from **33** and using thiophene‐2‐carbonyl chloride. Compound **34** was obtained as a brown solid in 50% yield. ^1^H NMR (DMSO‐*d*_6_ 400 MHz) δ: 3.74 (6H, s, OCH_3_), 6.27 (1H, s, Ar‐H), 7.02 (2H, s, Ar‐H), 7.23 (1H, d, *J* = 3.3 Hz, Ar‐H), 786 (1H, s, Ar‐H), 8.02 (1H, s, Ar‐H), and 10.13 (1H, bs, NH).

***N*‐(3,5‐dimethoxyphenyl)‐2‐naphthamide** (**35**). General procedure (**A**) staring from compound **33** and using 2‐naphthoyl chloride. Compound **35** was obtained as a gray solid in 96% yield. ^1^H NMR (400 MHz, CDCl_3_) δ: 3.86 (6H, s, OCH_3_), 6.33 (1H, s, Ar‐H), 6.99 (2H, s, Ar‐H), 7.59–7.65 (2H, m, NH and Ar‐H), 7.93–7.99 (5H, m, Ar‐H), and 8.40 (1H, s, Ar‐H).

***N*‐(3,5‐dimethoxyphenyl)benzamide** (**36**). General procedure (**A**) starting from **33** and using benzoyl chloride. Compound **36** was obtained as a yellow solid in 70% yield. ^1^H NMR (CDCl_3_ 400 MHz) δ: 3.84 (6H, s, OCH_3_ x 2), 6.29 (1H, t, *J* = 2.2 Hz, Ar‐H), 6.91 (2H, d, *J* = 2.2 Hz, Ar‐H), 7.47–7.51 (2H, m, Ar‐H), 7.53–7.58 (1H, m, Ar‐H), 7.80 (1H, bs, NH), and 7.86 (2H, d, *J* = 7.1 Hz, Ar‐H).

***N*‐(3,5‐dimethoxyphenyl)‐2‐propoxybenzamide** (**37**). General procedure (**A**) starting from **33** (1.03 g, 6.71 mmol) and using 2‐propoxybenzoyl chloride (1.6 g, 8.05 mmol). Compound **37** was obtained as a pink solid in 95% yield. ^1^H NMR (400 MHz, CDCl_3_) δ: 1.17 (3H, t, *J* = 7.4 Hz, OCH_2_ *CH*_3_), 1.98–2.10 (2H, m, OCH_2_ *CH*_2_CH_3_), 3.82 (6H, s, OCH_3_), 4.18 (2H, t, *J* = 6.6 Hz, O*CH*_2_CH_2_CH_3_), 6.27 (1H, t, *J* = 2.2 Hz, H6’), 6.95 (2H, d, *J* = 2.2 Hz, H2’ and H4’), 7.01 (1H, d, *J* = 8.3 Hz, aromatic H), 7.12 (1H, t, *J* = 7.3 Hz, aromatic H), 7.48 (1H, dt, *J* = 1.8 and 8.8 Hz, aromatic H), 8.29 (1H, dd, *J* = 1.7 and 7.8 Hz, aromatic H), and 10.08 (1H, s, NH).

***N*‐(3,5‐dimethoxyphenyl)‐4‐methoxybenzamide** (**38**). General procedure (**A**) starting from **33** and using 4‐methoxybenzoyl chloride. Compound **38** was obtained as a yellow solid in 98% yield. ^1^H NMR (400 MHz, CDCl_3_) δ: 3.83 (6H, s, OCH_3_ x2), 3.90 (3H, s, COCH_3_), 6.29 (1H, t, *J* = 2.2 Hz, Ar‐H), 6.92 (2H, d, *J* = 2.2 Hz, Ar‐H), 6.99 (2H, d, *J* = 8.8 Hz, Ar‐H), 7.77 (1H, bs, NH) and, 7.85 (2H, d, *J* = 8.8 Hz, Ar‐H).

**4‐Butoxy‐*N*‐(3,5‐dimethoxyphenyl)benzamide** (**39**). General procedure (**A**) starting from **33**. Compound **39** was obtained as a brown solid in 79% yield. ^1^H NMR (400 MHz, CDCl_3_) δ: 1.01 (3H, t, *J* = 7.4 Hz, OCH_2_CH_2_CH_2_ *CH*_3_), 1.50–1.56 (2H, m, OCH_2_CH_2_ *CH*_2_CH_3_), 1.80–1.84 (2H, m, OCH_2_ *CH*_2_CH_2_CH_3_), 3.83 (6H, s, OCH_3_), 4.05 (2H, t, *J* = 6.5 Hz, O*CH*_2_CH_2_CH_2_CH_3_), 6.29 (1H, s, Ar‐H), 6.92 (2H, d, *J* = 2.1 Hz, Ar‐H), 6.98 (2H, d, *J* = 8.8 Hz, Ar‐H), 7.72 (1H, bs, NH), and 7.84 (2H, d, *J* = 8.8 Hz, Ar‐H).

**4‐Butyl‐*N*‐(3,5‐dimethoxyphenyl)benzamide** (**40**). General procedure (**A**) starting from compound **33** and using 4‐butylbenzoyl chloride. Compound **40** was obtained as a white solid in 69% yield. ^1^H NMR (400 MHz, DMSO‐*d*_6_) δ: 0.97 (3H, t, *J* = 7.3 Hz, butyl CH_3_), 1.33–1.42 (2H, m, butyl CH_2_), 1.61–1.68 (2H, m, butyl CH_2_), 2.72 (2H, t, *J* = 7.5 Hz, butyl CH_2_), 3.79 (6H, s, OCH_3_), 6.32 (1H, t, Ar‐H), 7.15 (2H, d, *J* = 2.2 Hz, Ar‐H), 7.40 (2H, d, *J* = 8.2 Hz, Ar‐H), 7.92 (2H, d, *J* = 8.2 Hz, Ar‐H), and 10.13 (1H, s, NH).

***N*‐(2‐acetyl‐3,5‐dimethoxyphenyl)thiophene‐2‐carboxamide** (**41**). General procedure (**B**) starting from compound **34**. Derivative **41** was obtained as an orange solid in 43% yield. ^1^H NMR (400 MHz, DMSO‐*d*_6_) δ: 2.54 (3H, s, CH_3_), 3.85 (3H, s, OCH_3_), 3.91 (3H, s, OCH_3_), 6.50 (1H, s, Ar‐H), 7.26 (1H, s, Ar‐H), 7.55 (1H, s, Ar‐H), 7.78 (1H, s, Ar‐H), 7.91 (1H, s, Ar‐H), and 12.01 (1H, s, Ar‐H and NH).

***N*‐(2‐acetyl‐3,5‐dimethoxyphenyl)‐2‐naphthamide** (**42**). General procedure (**B**) starting from compound **35**. After flash column chromatography (Cy/EtOAc 80:20), compound **42** was obtained as a solid in 20% yield. ^1^H NMR (400 MHz, CDCl_3_) δ: 2.69 (3H, s, CH_3_), 3.94 (3H, s, OCH_3_), 3.98 (3H, s, OCH_3_), 6.29 (1H, d, *J* = 2.2 Hz, Ar‐H), 7.57–7.63 (2H, m, Ar‐H), 7.92–8.06 (4H, m, Ar‐H), 8.34 (1H, d, *J* = 2.2 Hz, Ar‐H), 8.61 (1H, s, Ar‐H), and 13.10 (1H, bs, NH).

***N*‐(2‐acetyl‐3,5‐dimethoxyphenyl)benzamide** (**43**). General procedure (**B**) starting from **36**. Compound **43** was obtained as a white solid in 38% yield. ^1^H NMR (CDCl_3_ 400 MHz) δ: 2.74 (3H, s, CH_3_), 3.90 (3H, s, OCH_3_), 3.93 (3H, s, OCH_3_), 6.25 (1H, s, Ar‐H), 7.52–7.62 (3H, m, Ar‐H), 8.05 (2H, d, *J* = 7.1 Hz, Ar‐H), 8.27 (1H, s, Ar‐H), and 12.88 (1H, s, NH).

***N*‐(2‐acetyl‐3,5‐dimethoxyphenyl)‐2‐propoxybenzamide** (**44**). General procedure (**B**) starting from **37**, compound **44** was obtained as a green solid in 52% yield. ^1^H NMR (400 MHz, CDCl_3_) δ: 0.99 (3H, t, *J* = 7.4 Hz, OCH_2_CH_2_ *CH*_3_), 1.91–1.99 (2H, m, OCH_2_ *CH*_2_CH_3_), 2.49 (3H, s, CH_3_), 3.90 (3H, s, OCH_3_), 3.91 (3H, s, OCH_3_), 4.23 (2H, t, *J* = 6.9 Hz, O*CH*_2_CH_2_CH_3_), 6.26 (1H, d, *J* = 2.3 Hz, H4’), 6.99–7.06 (2H, m, aromatic H), 7.44 (1H, dt, *J* = 1.8 and 7.4 Hz, aromatic H), 7.97 (1H, d, *J* = 2.3 Hz, H6), 8.04 (1H, dd, *J* = 1.8 and 7.8 Hz, aromatic H), and 11.75 (1H, s, NH).

***N*‐(2‐acetyl‐3,5‐dimethoxyphenyl)‐4‐butoxybenzamide** (**46**). General procedure (**B**) starting from **39**. After automatic flash column chromatography (Cy/EtOAc 0 to 30%), compound **46** was obtained as a white solid in 33% yield. ^1^H NMR (400 MHz, CDCl_3_) δ: 1.01 (3H, t, *J* = 7.3 Hz, OCH_2_CH_2_CH_2_ *CH*_3_), 1.50–1.56 (2H, m, OCH_2_CH_2_ *CH*_2_CH_3_), 1.80–1.84 (2H, m, OCH_2_ *CH*_2_CH_2_CH_3_), 2.65 (3H, s, CH_3_), 3.91 (3H, s, OCH_3_), 3.95 (3H, s, OCH_3_), 4.05 (2H, t, *J* = 6.5 Hz, O*CH*_2_CH_2_CH_2_CH_3_), 6.25 (1H, d, *J* = 2.1 Hz, Ar‐H), 7.01 (2H, d, *J* = 8.8 Hz, Ar‐H), 8.02 (2H, d, *J* = 8.8 Hz, Ar‐H), 8.28 (2H, d, *J* = 2.3 Hz, Ar‐H), 12.83 (1H, bs, NH).

***N*‐(2‐acetyl‐3,5‐dimethoxyphenyl)‐4‐butylbenzamide** (**47**). General procedure (**B**) starting from compound **40**. Derivative **47** as a yellow solid in 70%. ^1^H NMR (400 MHz, DMSO‐*d*_6_) δ: 0.82 (3H, t, *J* = 7.3 Hz, butyl CH_3_), 1.18–1.28 (2H, m, butyl CH_2_), 1.46–1.54 (2H, m, butyl CH_2_), 2.48 (3H, s, CH_3_), 2.57 (2H, t, *J* = 7.5 Hz, butyl CH_2_), 3.71 (3H, s, OCH_3_), 3.74 (3H, s, OCH_3_), 6.39 (1H, d, *J* = 2.3 Hz, Ar‐H), 7.30 (2H, d, *J* = 8.2 Hz, Ar‐H), 7.67 (1H, d, *J* = 2.3 Hz, Ar‐H), 7.74 (2H, d, *J* = 7.8 Hz, Ar‐H), and 11.99 (1H, s, NH).

**5,7‐Dimethoxy‐2‐(2‐thienyl)quinolin‐4‐ol** (**48**). General procedure (**C**) starting from **41**, T = r.t. Compound **48** was obtained as a yellow solid in 61% yield. ^1^H NMR (400 MHz, DMSO‐*d*_6_) δ: 3.90 (6H, s, OCH_3_), 6.50 (1H, d, *J* = 2.1 Hz, Ar‐H), 6.78 (1H, s, Ar‐H), 6.90 (1H, d, *J* = 2.2 Hz, Ar‐H), 7.25 (1H, m, Ar‐H), 7.79 (1H, d, *J* = 4.5 Hz, Ar‐H), 7.91 (1H, d, *J* = 2.7 Hz, Ar‐H), and 12.00 (1H, s, OH).

**5,7‐Dimethoxy‐2‐(2‐naphthyl)quinolin‐4‐ol** (**49**). General procedure (**C**) starting from compound **42**, T = r.t. After purification by flash column chromatography (CHCl_3_/MeOH 95:5), compound **49** was obtained as a yellow solid in 31% yield. ^1^H NMR (400 MHz, DMSO‐*d*_6_) δ: 3.80 (3H, s, OCH_3_), 3.86 (3H, s, OCH_3_), 6.27 (1H, s, Ar‐H), 6.34 (1H, s, Ar‐H), 6.85 (1H, s, Ar‐H), 7.59–7.65 (2H, m, Ar‐H), 7.89–7.91 (1H, m, Ar‐H), 7.98–8.11 (3H, m, Ar‐H), 8.42 (1H, s, Ar‐H), and 11.25 (1H, bs, OH).

**5,7‐Dimethoxy‐2‐phenylquinolin‐4‐ol** (**50**). General procedure (**C**) starting from **43**, T = r.t. Compound **50** was obtained as a yellow solid in 68% yield. ^1^H NMR (DMSO‐_*d6*_, 400 MHz) δ:3.86 (3H, s, OCH_3_), 3.92 (3H, s, OCH_3_), 6.49 (1H, d, *J* = 1.7 Hz, Ar‐H), 6.52 (1H, bs, Ar‐H), 6.96 (1H, s, Ar‐H), 7.59–7.61 (3H, m, Ar‐H), 7.86–7.87 (2H, m, Ar‐H), and 12.03 (1H, s, OH).

**5,7‐Dimethoxy‐2‐(2‐propoxyphenyl)quinolin‐4‐ol** (**51**). General procedure (**C**) starting from **44**, T = r.t. Compound **51** was obtained as a brown solid in 26% yield. ^1^H NMR (400 MHz, DMSO‐*d*_6_) δ: 0.91 (3H, t, *J* = 7.4 Hz, OCH_2_CH_2_ *CH*_3_), 1.66–1.71 (2H, m, OCH_2_ *CH*_2_CH_3_), 3.83 (3H, s, CH_3_), 3.83 (3H, s, CH_3_), 4.01 (2H, t, *J* = 6.3 Hz, O*CH*_2_CH_2_CH_3_), 6.09 (1H, s, aromatic H), 6.40 (1H, d, *J* = 1.8 Hz, H6), 6.69 (1H, d, *J* = 1.8 Hz, H8), 7.10 (1H, t, *J* = 7.4 Hz, aromatic H), 7.20 (1H, d, *J* = 8.3 Hz, aromatic H), 7.48–7.52 (2H, m, aromatic H), and 11.5 (1H, bs, OH).

**2‐(4‐Butoxyphenyl)−5,7‐dimethoxyquinolin‐4‐ol** (**53**). General procedure (**C**) starting from **46**, T = 50°C. Compound **53** was obtained as a yellow solid in 74% yield. ^1^H NMR (400 MHz, DMSO‐*d*_6_) δ: 0.95 (3H, t, *J* = 7.3 Hz, OCH_2_CH_2_CH_2_ *CH*_3_), 1.43–1.49 (2H, m, OCH_2_CH_2_ *CH*_2_CH_3_), 1.70–1.77 (2H, m, OCH_2_ *CH*_2_CH_2_CH_3_), 3.85 (3H, s, OCH_3_), 3.88 (3H, s, OCH_3_), 4.85 (2H, t, *J* = 6.4 Hz, O*CH*_2_CH_2_CH_2_CH_3_), 6.48 (1H, s, Ar‐H), 6.50 (1H, s, Ar‐H), 6.96 (1H, s, Ar‐H), 7.14 (2H, d, *J* = 8.6 Hz, Ar‐H), and 7.83 (2H, d, *J* = 8.5 Hz, Ar‐H).

**2‐(4‐Butylphenyl)−5,7‐dimethoxyquinolin‐4‐ol** (**54**). General procedure (**C**) starting from compound **47**, T = 50°C. Derivative **54** was obtained as a yellow solid in 95% yield. ^1^H NMR (400 MHz, DMSO‐*d*_6_) δ: 0.91 (3H, t, *J* = 7.3 Hz, butyl CH_3_), 1.28–1.37 (2H, m, butyl CH_2_), 1.55–1.63 (2H, m, butyl CH_2_), 2.65 (2H, t, *J* = 7.4 Hz, butyl CH_2_), 3.79 (3H, s, OCH_3_), 3.83 (3H, s, OCH_3_), 6.17 (1H, s, Ar‐H), 6.34 (1H, s, Ar‐H), 6.84 (1H, s Ar‐H), 7.34 (2H, d, *J* = 7.8 Hz, Ar‐H), 7.73 (2H, d, *J* = 6.9 Hz, Ar‐H), and 11.20 (1H, bs, OH).

**4‐(2‐Chloroethoxy)−5,7‐dimethoxy‐2‐(2‐thienyl)quinoline** (**55**). General procedure (**D**) starting from **48** and using 1‐bromo‐2‐chloroethane, time = 48 h. Compound **55** was obtained as a white solid in 41% yield. ^1^H NMR (400 MHz, CDCl_3_) δ: 3.87 (3H, s, OCH_3_), 3.91 (3H, s, OCH_3_), 4.05–4.07 (2H, m, CH_2_Cl), 4.55–4.58 (2H, m, OCH_2_), 6.57 (1H, s, Ar‐H), 6.93 (1H, s, Ar‐H), 7.25 (1H, s, Ar‐H), 7.33 (1H, s, Ar‐H), 7.77 (1H, s, Ar‐H), and 8.07 (1H, s, Ar‐H).

**4‐(2‐Chloroethoxy)−5,7‐dimethoxy‐2‐(2‐naphthyl)quinoline** (**56**). General procedure (**D**) starting from compound **49** and using 1‐bromo‐2‐chloroethane, time = 5 h. Compound **56** was obtained as a yellow solid in 76% yield. ^1^H NMR (400 MHz, CDCl_3_) δ: 3.98 (3H, s, OCH_3_), 3.99 (3H, s, OCH_3_), 4.04 (2H, t, *J* = 5.7 Hz, CH_2_Cl), 4.54–4.55 (2H, m, OCH_2_), 6.53 (1H, s, Ar‐H), 7.17–7.18 (2H, m, Ar‐H), 7.54–7.55 (2H, m, Ar‐H), 7.91–7.93 (1H, m, Ar‐H), 7.99–8.01 (2H, m, Ar‐H), 8.26–8.28 (1H, m, Ar‐H), and 8.55–8.57 (1H, s, Ar‐H).

**4‐(2‐Chloroethoxy)−5,7‐dimethoxy‐2‐phenylquinoline** (**57**). General procedure (**D**) starting from **50** and using 1‐bromo‐2‐chloroethane, time = 24 h. Compound **57** was obtained as yellow solid in 65% yield. ^1^H NMR (DMSO‐_*d6*_, 400 MHz) δ: 3.94 (3H, s, OCH_3_), 3.95 (3H, s, OCH_3_), 3.98 (2H, t, *J* = 6.0 Hz, OCH_2_ *CH*_2_Cl), 4.47 (2H, t, *J* = 5.9 Hz, O*CH*_2_CH_2_Cl), 6.50 (1H, d, *J* = 2.3 Hz, Ar‐H), 7.01 (1H, s, Ar‐H), 7.09 (1H, bs, Ar‐H), 7.43 – 7.53 (3H, m, Ar‐H),and 8.06 (2H, d, *J* = 7.2 Hz, Ar‐H).

**4‐(2‐Chloroethoxy)−5,7‐dimethoxy‐2‐(2‐propoxyphenyl)quinoline** (**58**). General procedure (**D**) starting from **51** and using 1‐bromo‐2‐chloroethane, the target compound **58** was obtained as a pink solid in 47% yield. ^1^H NMR (400 MHz, CDCl_3_) δ: 0.99 (3H, t, *J* = 7.4 Hz, OCH_2_CH_2_ *CH*_3_), 1.77–1.78 (2H, m, OCH_2_ *CH*_2_CH_3_), 3.93–4.00 (10H, m, OCH_3_, OCH_3_, O*CH*_2_CH_2_CH_3_ and CH_2_Cl), 4.40 (2H, t, *J* = 6.1 Hz, OCH_2_), 6.50 (1H, d, *J* = 2.3 Hz, H6), 7.00 (1H, d, *J* = 8.2 Hz, aromatic H), 7.23 (1H, s, aromatic H), 7.37 (1H, m, aromatic H), and 7.88 (1H, dd, *J* = 1.8 and 7.6 Hz, aromatic H).

**4‐(2‐Chloroethoxy)−5,7‐dimethoxy‐2‐(4‐methoxyphenyl)quinoline** (**59**). General procedure (**D**) starting from compound **52** and using 1‐bromo‐2‐chloroethane, time = 4 h. Compound **59** was obtained as a white solid in 80% yield. ^1^H NMR (400 MHz, CDCl_3_) δ: 3.90 (3H, s, OCH_3_), 3.95 (3H, s, OCH_3_), 3.98 (3H, s, OCH_3_), 3.99 (2H, t, *J* = 5.9 Hz, CH_2_Cl), 4.47 (2H, t, *J* = 5.9 Hz, OCH_2_), 6.49 (1H, d, *J* = 2.1 Hz, Ar‐H), 6.98 (1H, s, Ar‐H), 7.04 (2H, d, *J* = 8.8 Hz, Ar‐H), 7.07 (1H, s, *J* = 2.0 Hz, Ar‐H), and 8.06 (2H, d, *J* = 8.8 Hz, Ar‐H).

**2‐(4‐Butoxyphenyl)‐4‐(2‐chloroethoxy)−5,7‐dimethoxyquinoline** (**60**). General procedure (**D**) starting from compound **53** and using 1‐bromo‐chloroethane, time = 12 h. Compound **60** was obtained as a pink solid in 68% yield. ^1^H NMR (400 MHz, DMSO‐*d*_6_) δ: 0.96 (3H, t, *J* = 7.3 Hz, OCH_2_CH_2_CH_2_ *CH*_3_), 1.44–1.50 (2H, m, OCH_2_CH_2_ *CH*_2_CH_3_), 1.70–1.75 (2H, m, OCH_2_ *CH*_2_CH_2_CH_3_), 3.85 (3H, s, OCH_3_), 3.89 (3H, s, OCH_3_), 4.03–4.07 (4H, m, O*CH*_2_CH_2_CH_2_CH_3_ and CH_2_Cl) 4.53 (2H, t, *J* = 4.8 Hz, OCH_2_), 6.52 (1H, s, Ar‐H), 6.94 (1H, s, Ar‐H), 7.05 (2H, d, *J* = 8.6 Hz, Ar‐H), 7.27 (1H, s, Ar‐H), and 8.13 (2H, d, *J* = 8.6 Hz, Ar‐H).

**2‐(4‐Butylphenyl)‐4‐(2‐chloroethoxy)−5,7‐dimethoxyquinoline** (**61**). General procedure (**D**) starting from compound **54** and using 1‐bromo‐2chloroethane, time = 3 h. Compound **61** was obtained as a yellow solid in 25% yield. ^1^H NMR (400 MHz, CDCl_3_) δ: 0.94 (3H, t, *J* = 7.3 Hz, butyl CH_3_), 1.33–1.42 (2H, m, butyl CH_2_), 1.60–1.68 (2H, m, butyl CH_2_), 2.68 (2H, t, *J* = 7.6 Hz, butyl CH_2_), 3.93 (6H, s, OCH_3_), 3.96 (2H, t, *J* = 5.9 Hz, CH_2_Cl), 4.46 (2H, t, *J* = 5.9 Hz, OCH_2_), 6.48 (1H, d, *J* = 2.1 Hz, Ar‐H), 6.99 (1H, s, Ar‐H), 7.31 (2H, d, *J* = 8.0 Hz, Ar‐H), and 7.97 (2H, d, *J* = 8.0 Hz, Ar‐H).

**4‐(2‐Chloroethoxy)−5,7‐dimethoxyquinoline** (**64**). To a solution of PPh_3_ (0.188 g, 0.72 mmol) in dry THF (2 mL), DIAD (0.054 mL, 0.27 mmol) was added at 0°C under a nitrogen atmosphere. They reacted at r.t until the formation of a white precipitate, then **63** (0.05 g, 0.24 mmol) and 2‐chloroethanol (0.022 mL, 0.27 mmol) were added. The reaction mixture was stirred at rt for 48 h. Then, it was poured into ice/water and the obtained precipitate was filtered and purified by flash column chromatography eluted with CHCl_3_/MeOH 99:1, to obtain compound **64** as a solid in 38% yield. ^1^H NMR (400 MHz, CDCl_3_) δ: 3.95 (6H, s, OCH_3_), 3.97–3.99 (2H, m, CH_2_Cl), 4.40 (2H, t, *J* = 5.8 Hz, OCH_2_), 6.52 (1H, s, Ar‐H), 6.60 (1H, d, *J* = 5.1 Hz, Ar‐H), 7.02 (1H, s, Ar‐H), and 8.60 (1H, d, *J* = 5.0 Hz, Ar‐H).

**Ethyl 3‐[4‐(benzyloxy)phenyl]‐3‐oxopropanoate** (**68**)**.** Under N_2_ atmosphere, NaH 60%(1.08 g, 27.00 mmol) was suspended in dry THF (15 mL). Then, compound **65** (3.00g, 13.00 mmol) was added and after 10 min diethyl carbonate (4.82 mL, 39.00 mmol) was also added dropwise. The reaction was stirred at 70°C for 24 h. The mixture was poured into ice/water and extracted with EtOAc (x3). The organic layer was washed with brine, dried over Na_2_SO_4_ and evaporated to dryness under reduced pressure to obtain intermediate **68** as a yellow oil. (3.65g, 94% yield). ^1^H NMR (400 MHz, CDCl_3_) δ = 1.28 (3H, t, *J* = 7.1 Hz, OCH_2_ *CH*_3_), 3.96 (2H, s, CH_2_), 4.23 (2H, q, *J* = 7.1 Hz, O*CH*_2_CH_3_), 5.15 (2H, d, *J* = 10.6 Hz, benzylic CH_2_), 7.03 (2H, d, *J* = 8.8 Hz, Ar‐H), 7.37–7.46 (5H, m, Ar‐H), and 7.95 (2H, d, *J* = 8.8 Hz, Ar‐H).

**Ethyl 3‐(4‐butoxyphenyl)‐3‐oxopropanoate** (**70**). To a suspension of NaH (1.0 g, 25 mmol) in dry THF (12 mL), a solution of **67** (2.4 g, 12.5 mmol) in dry THF (3 mL) was added at 0°C under a nitrogen atmosphere. They reacted at r.t. for 15 min, then diethylcarbonate (4.5 mL, 37.5 mmol) was added dropwise at 0°C. The reaction mixture was stirred at 70°C 12 h. Then, EtOAc was slowly added, and the mixture was poured into ice/water. It was extracted with EtOAc, the organic layers were washed with brine, dried over Na_2_SO_4_ and evaporated under reduced pressure obtaining **70** as a brown oil in 95% yield. ^1^H NMR (400 MHz, CDCl_3_) δ: 0.85 (3H, t, *J* = 7.4 Hz, butoxy CH_3_), 1.12 (3H, t, *J* = 7.1 Hz, OCH_2_ *CH*_3_), 1.26–1.39 (2H, m, butoxy CH_2_), 1.60–1.67 (2H, m, butoxy CH_2_), 3.78 (2H, s, CH_2_), 3.87 (2H, t, *J* = 6.5 Hz, butoxy CH_2_), 4.04–4.11 (2H, m, O*CH*_2_CH_3_), 6.77 (2H, d, *J* = 8.9 Hz, H3 and H5), and 7.76 (2H, d, *J* = 8.9 Hz, H2 and H6).

**Ethyl (3*E*)‐3‐[4‐(benzyloxy)phenyl]‐3‐[(3,5‐dimethoxyphenyl)imino]propanoate** (**71**). General procedure (**F**), starting from **68** (4.7g, 12 mmol) 3,5‐dimethoxy aniline (9.4 g, 60 mmol). After purification by flash column chromatography eluted with Cy/EtOAc 90:10, compound **71** was obtained as a yellow oil in 47% yield. ^1^H NMR (400 MHz, CDCl_3_) δ: 1.33 (3H, t, *J* = 7.1 Hz, OCH_2_ *CH*_3_), 3.58 (6H, s, OCH_3_), 4.21 (2H, q, *J* = 7.1 Hz, O*CH*_2_CH_3_), 4.99 (1H, s, CH), 5.09 (2H, s, benzylic CH_2_), 5.86 (2H, d, *J* = 2.2 Hz, Ar‐H), 6.07 (1H, t, *J* = 2.2 Hz, Ar‐H), 6.93 (2H, d, *J* = 8.9 Hz, Ar‐H), 7.29–7.45 (7H, m, Ar‐H), and 10.20 (1H, bs, OH).

**Ethyl (3*E*)‐3‐[(3,5‐dimethylphenyl)imino]‐3‐(4‐propoxyphenyl)propanoate** (**72**). General procedure (**F**), starting from **69** [23] (4.0 g, 16 mmol) and using 3,5‐dimethylaniline (12.25 g, 80 mmol). After purification with automatic flash column chromatography eluted with Cy/EtOAc from 0 to 30%, compound **72** was obtained as a brown oil in 44% yield. ^1^H NMR (400 MHz, CDCl_3_) δ: 0.98 (3H, t, *J* = 7.4 Hz, OCH_2_CH_2_ *CH*_3_), 1.23 (3H, t, *J* = 7.1 Hz, OCH_2_ *CH*_3_), 1.69–1.76 (2H, m, OCH_2_ *CH*_2_CH_3_), 2.03 (6H, s, CH_3_), 3.83 (2H, t, *J* = 6.6 Hz, O*CH*_2_CH_2_CH_3_), 4.10 (2H, q, *J* = 7.1 Hz, O*CH*_2_CH_3_), 4.86 (1H, s, CH), 6.23 (2H, s, Ar‐H), 6.47 (1H, s, Ar‐H), 6.71 (2H, d, *J* = 8.7 Hz, Ar‐H), 7.20 (2H, d, *J* = 8.8 Hz, Ar‐H), and 10.12 (1H, bs, OH).

**Ethyl (3*E*)‐3‐(4‐butoxyphenyl)‐3‐[(2,5‐dimethoxyphenyl)imino]propanoate** (**74**). General procedure (**F**), starting from **70** (3.12 g, 11.3 mmol) and using 2,5‐dimethoxyaniline (6.70 g, 45 mmol). After purification by flash chromatography column eluted with Cy/EtOAc 80/20, compound **74** was obtained as an oil in 58% yield. ^1^H NMR (400 MHz, CDCl_3_) δ: 0.90 (3H, t, *J* = 6.6 Hz, butoxy CH_3_), 1.22 (3H, t, *J* = 7.1 Hz, OCH_2_ *CH*_3_), 1.38–1.45 (2H, m, butoxy CH_2_), 1.65–1.73 (2H, m, butoxy CH_2_), 3.31 (3H, s, OCH_3_), 3.79 (3H, s, OCH_3_), 3.86–3.90 (2H, m, O*CH*_2_CH_3_), 4.13 (2H, q, *J* = 7.1 Hz, butoxy CH_2_),4.91 (1H, s, CH), 5.79 (1H, s, H6’), 6.30 (1H, d, *J* = 8.8 Hz, H4’), 6.70 (1H, d, *J* = 8.8 Hz, H5’), 6.76 (2H, d, *J* = 8.6 Hz, H3 and H5), 7.23 (2H, d, *J* = 8.5 Hz, H2 and H6), and 10.15 (1H, bs, OH).

**Ethyl (3Z)‐3‐[(2,5‐diethoxyphenyl)imino]‐3‐(4‐propoxyphenyl)propanoate** (**75**)**.** General procedure (**F**), starting from **69** [23], and using 2,5‐diethoxy aniline. After purification by flash column chromatography, eluted with cyclohexane/ EtOAc 90/10 compound **75** was obtained as a yellow oil in 59% yield. ^1^H NMR (400 MHz, CDCl_3_) δ: 0.98 (3H, t, *J* = 6.8 Hz, OCH_2_CH_2_ *CH*_3_), 1.02 (3H, t, *J* = 7.4 Hz, OCH_2_ *CH*_3_), 1.34 (3H, t, *J* = 7.1 Hz, OCH_2_ *CH*_3_), 1.47 (3H, t, *J* = 6.9 Hz, OCH_2_ *CH*_3_), 1.75–1.83 (2H, m, OCH_2_ *CH*_2_CH_3_), 3.52 (2H, q, *J* = 6.9 Hz, O*CH*_2_CH_3_), 3.91 (2H, t, *J* = 6.5 Hz, O*CH*_2_CH_2_CH_3_), 4.05 (2H, q, *J* = 6.9 Hz, O*CH*_2_CH_3_), 4.18 (2H, q, *J* = 7.1 Hz, O*CH*_2_CH_3_), 4.97 (1H, s, CH), 5.85 (1H, d, *J* = 2.9 Hz, H2), 6.36 (1H, dd, *J* = 2.9 and 8.8 Hz, H4), 6.73 (1H, d, *J* = 8.8 Hz, H5), 6.83 (2H, d, *J* = 8.8 Hz, H3’ and H5’), 7.30 (2H, d, *J* = 8.8 Hz, H3’ and H5’), and 10.20 (1H, s, OH).

**2‐[4‐(Benzyloxy)phenyl]−5,7‐dimethoxyquinolin‐4‐ol** (**76**). General procedure (**G**) starting from compound **71**, time = 4 h. Derivative **76** was obtained after treatment with refluxed Et_2_O/EtOH, as a brown solid in 45% yield. ^1^H NMR (400 MHz, DMSO‐*d*_6_) δ: 3.35 (3H, s, OCH_3_), 3.83 (6H, s, OCH_3_), 5.21 (2H, s, Bn CH_2_), 6.06 (1H, s, Ar‐H), 6.31 (1H, s, Ar‐H), 6.79 (1H, s, Ar‐H), 7.18 (2H, d, *J* = 8.5 Hz, Ar‐H), 7.33–7.49 (5H, m, Ar‐H), 7.75 (2H, d, *J* = 7.7 Hz, Ar‐H), and 11.07 (1H, bs, OH).

**5,7‐Dimethyl‐2‐(4‐propoxyphenyl)quinolin‐4‐ol** (**77**). General procedure (**G**) starting from compound **72**. Derivative **77** was obtained as a brown solid in 80% yield. ^1^H NMR (400 MHz, CDCl_3_) δ: 0.99 (3H, t, *J* = 7.3 Hz, OCH_2_CH_2_ *CH*_3_), 1.72–1.78 (2H, m, OCH_2_ *CH*_2_CH_3_), 2.35 (3H, s, CH_3_), 2.78 (3H, s, CH_3_), 4.02 (2H, t, *J* = 6.4 Hz, O*CH*_2_CH_2_CH_3_), 6.82 (1H, s, Ar‐H), 6.99–7.02 (1H, m, Ar‐H), 7.11 (2H, d, *J* = 8.4 Hz, Ar‐H), 7.37 (1H, s, Ar‐H), 7.74 (2H, d, *J* = 8.4 Hz, Ar‐H), and 11.17 (1H, s, OH).

**2‐(4‐Butoxyphenyl)−5,8‐dimethoxyquinolin‐4‐ol** (**79**)**.** General procedure (**G**) starting from compound **74** (2.65 g, 6.63 mmol). After purification by automatic flash column chromatography eluted with CHCl_3_/MeOH from 1 to 5%, compound **79** was obtained as a yellow solid in 37% yield. ^1^H NMR (400 MHz, DMSO‐*d*_6_) δ: 1.00 (3H, t, *J* = 7.3 Hz, butoxy CH_3_), 1.48–1.54 (2H, m, butoxy CH_2_), 1.75–1.82 (2H, m, butoxy CH_2_), 3.81 (3H, s, OCH_3_), 3.98 (3H, s, OCH_3_), 4.10 (2H, t, *J* = 6.4 Hz, butoxy CH_2_), 6.22 (1H, s, H3), 6.74 (1H, d, *J* = 8.8 Hz, H6), 7.10–7.13 (2H, m, H3’ and H5’), 7.74 (2H, d, *J* = 8.5 Hz, H2’ and H6’), 8.22 (1H, d, *J* = 8.3 Hz, H7), and 10.02 (1H, bs, OH).

**5,8‐Diethoxy‐2‐(4‐propoxyphenyl)quinolin‐4‐ol** (**80**)**.** General procedure (**G**) starting from compound **75**, after flash column chromatography eluted with CHCl_3_/MeOH 98:2, intermediate **80** was obtained as a solid in 34% yield. ^1^H NMR (400 MHz, DMSO‐*d*_6_) δ: 1.01 (3H, t, *J* = 7.4 Hz, OCH_2_CH_2_ *CH*_3_), 1.44 (6H, t, *J* = 6.9 Hz, OCH_2_ *CH*_3_), 1.73–1.82 (2H, m, OCH_2_ *CH*_2_CH_3_), 4.00–4.04 (2H, m, O*CH*_2_CH_3_ and O*CH*_2_CH_2_CH_3_), 4.19 (2H, q, *J* = 6.8 Hz, O*CH*_2_CH_3_), 6.15 (1H, s, H3), 6.69 (1H, d, *J* = 7.8 Hz, H6), 7.09–7.13 (2H, m, aromatic H), 7.68 (2H, d, *J* = 6.7 Hz, aromatic H), 8.17–8.18 81H, m, aromatic H), and 9.83 (1H, s, OH).

**2‐[4‐(Benzyloxy)phenyl]‐4‐(2‐chloroethoxy)−5,7‐dimethoxyquinoline** (**81**). General procedure (**D**) starting from compound **76** and using 1‐bromo‐2‐chloroethane, time = 4 h. ^1^H NMR (400 MHz, CDCl_3_) δ: 3.86 (3H, s, OCH_3_), 3.89 (3H, s, OCH_3_), 4.06 (2H, t, *J* = 5.3 Hz, CH_2_Cl), 4.55 (2H, t, *J* = 5.1 Hz, OCH_2_), 5.21 (2H, s, benzylic CH_2_), 6.53 (1H, d, *J* = 2.0 Hz, Ar‐H), 6.95 (1H, d, *J* = 2.2 Hz, Ar‐H), 7.15 (2H, d, *J* = 8.9 Hz, Ar‐H), 7.28 (1H, s, Ar‐H), 7.34–7.44 (3H, m, Ar‐H), 7.50 (2H, d, *J* = 7.5 Hz, Ar‐H), and 8.23 (2H, d, *J* = 8.9 Hz, Ar‐H).

**4‐(2‐Chloroethoxy)−5,7‐dimethyl‐2‐(4‐propoxyphenyl)quinoline** (**82**). General procedure (**D**) starting from compound **77** and using 1‐bromo‐2‐chloroethane, time = 5 h. Compound **82** was obtained as a brown solid in 70% yield. ^1^H NMR (400 MHz, CDCl_3_) δ: 0.99 (3H, t, *J* = 7.4 Hz, OCH_2_CH_2_ *CH*_3_), 1.73–1.82 (2H, m, OCH_2_ *CH*_2_CH_3_), 2.40 (3H, s, CH_3_), 2.81 (3H, s, CH_3_), 3.91–3.94 (4H, m, O*CH*_2_CH_2_CH_3_ and CH_2_Cl), 4.46 (2H, t, *J* = 5.5 Hz, OCH_2_), 6.87–6.98 (4H, m, Ar‐H), 7.64 (1H, s, Ar‐H), and 8.06 (2H, d, *J* = 6.9 Hz, Ar‐H).

**2‐(4‐Butoxyphenyl)‐4‐(2‐chloroethoxy)−5,8‐dimethoxyquinoline** (**84**). General procedure (**D**) starting from **79** and using 1‐bromo‐2‐chloroethane, time = 3 h. Compound **84** was obtained as a white solid in 38% yield. ^1^H NMR (400 MHz, CDCl_3_) δ: 0.93 (3H, t, *J* = 7.2 Hz, butoxy CH_3_), 1.43–1.48 (2H, m, butoxy CH_2_), 1.72–1.75 (2H, m, butoxy CH_3_), 3.86 (3H, s, OCH_3_), 3.92–3.97 (7H, m, OCH_3_, butoxy CH_2_ and CH_2_), 4.44–4.46 (2H, m, OCH_2_), 6.71 (1H, d, *J* = 8.0 Hz, H6), 6.89–6.95 (3H, m, H3’, H5’ and H7), 7.09 (1H, s, H3), and 8.03 (2H, d, *J* = 8.1 Hz, H2’ and H6’).

**4‐(2‐Chloroethoxy)−5,8‐diethoxy‐2‐(4‐propoxyphenyl)quinoline** (**85**)**.** General procedure (**D**) starting from **80** and using 1‐bromo‐2‐chloroethane, time = 5 h. Compound **85** was obtained as a solid in 98% yield. ^1^H NMR (400 MHz, DMSO‐*d*_6_) δ: 1.05 (3H, t, *J* = 7.4 Hz, OCH_2_CH_2_ *CH*_3_), 1.42–1.46 (6H, m, OCH_2_ *CH*_3_), 1.75–1.80 (2H, m, OCH_2_ *CH*_2_CH_3_), 4.01–4.05 (4H, m, O*CH*_2_CH_3_ and O*CH*_2_CH_2_CH_3_), 4.08 (2H, t, *J* = 4.8 Hz, CH_2_Cl), 4.20 (2H, q, *J* = 6.9 Hz, O*CH*_2_CH_3_), 4.58 (2H, t, *J* = 4.6 Hz, OCH_2_), 6.81 (1H, d, *J* = 8.6 Hz, H6), 7.08 (3H, d, *J* = 8.5 Hz, H7 and H3’ and H5’), 9.93 (1H, s, H3), and 8.26 (2H, d, *J* = 8.7 Hz, H2’ and H6’).

**4‐(2‐Chloroethoxy)−5,6,7‐trimethoxy‐2‐(4‐propoxyphenyl)quinoline** (**87**)**.** General procedure (**D**), starting from **86** and using 1‐bromo‐2‐chloroethane, time = 3 h. Compound **87** was obtained as a pink solid in 47% yield. ^1^H NMR (400 MHz, CDCl_3_) δ: 1.07 (3H, t, *J* = 7.4 Hz, OCH_2_CH_2_ *CH*_3_), 1.81–1.90 (2H, m, OCH_2_ *CH*_2_CH_3_), 3.82–4.04 (13H, m, OCH_3_, CH_2_Cl and O*CH*_2_CH_2_CH_3_), 4.51 (2H, t, *J* = 5.6 Hz, OCH_2_), 6.99 (1H, s, H3), 7.02 (2H, d, *J* = 8.8 Hz, H3’ and H5’), 7.30 (1H, s, H8), and 8.02 (2H, d, *J* = 8.8 Hz, H2’ and H6’).

### 4.2 Biological Procedures

#### 4.2.1 Cells and Viruses

The SARS‐CoV‐2 isolate used in this study was the BetaCov/Belgium/GHB‐03021/2020 (EPI ISL407976|2020‐02−03), which was isolated from a Belgian patient returning from Wuhan in February 2020. The isolate was passaged 7 times on Vero E6 cells, which introduced two series of amino acid deletions in the spike protein [35]. The infectious content of the virus stock was determined by titration on Vero E6 cells. Vero E6 cells were maintained in Dulbecco's modified Eagle's medium (DMEM; Gibco) supplemented with heat‐inactivated 10% v/v fetal calf serum (FCS; Biowest) and 500 µg/mL Geneticin (Gibco) and kept under 5% CO_2_ at 37°C. All SARS‐CoV‐2‐related experimental work was performed in the certified, high‐containment biosafety level‐3 facilities of the Rega Institute at the KU Leuven (Belgium).

In the BSL2 facility, human embryonic lung fibroblasts (**HEL** 299; ATCC CCL‐137) were cultured in Dulbecco's modified Eagle's medium (DMEM; Gibco catalog no. 41 965–039) supplemented with 8% fetal bovine serum (FBS). The **HCoV‐229E** (ATCC VR‐740) and **HCoV‐OC43** (ATCC VR‐1558) stocks were obtained by inoculating a confluent monolayer of HEL 299 or HRT‐18G cells, respectively. The supernatant was harvested after 3 days of incubation for HCoV‐229E, or 7 days of incubation for HCoV‐OC43, at 35°C under 5% CO_2_. After one freeze–thaw cycle and removal of cellular debris by centrifugation, aliquots were stored at −80°C.

#### 4.2.2 SARS‐CoV‐2 Screening

The SARS‐CoV‐2 antiviral assay is derived from the previously established SARS‐CoV assay [25]. In this assay, the fluorescence of Vero E6‐GFP cells (African monkey kidney cell line expressing green fluorescent protein; provided by M. van Loock, Janssen Pharmaceutica declines after infection with SARS‐CoV‐2 due to the cytopathogenic effect of the virus. In the presence of an antiviral compound, the cytopathogenicity is inhibited and the fluorescent signal is rescued. Stock solutions of the various compounds in DMSO (10 mM) were prepared. On day −1, the test compounds were serially diluted in assay medium (DMEM supplemented with 2% v/v FCS). The plates were incubated (37°C, 5% CO_2_ and 95% relative humidity) overnight. On day 0, the diluted compounds were then mixed with SARS‐CoV‐2 at 20 TCID50/well and Vero E6‐eGFP cells corresponding to a final density of 25,000 cells/well in 96‐well blackview plates (Greiner Bio‐One, Vilvoorde, Belgium).

The plates were incubated in a humidified incubator at 37°C and 5% CO_2_. At 4 days p.i., the wells were examined for eGFP expression using an argon laser‐scanning microscope. The microscope settings were excitation at 488 nm and emission at 510 nm and the fluorescence images of the wells were converted into signal values. The results were expressed as EC_50_ values defined as the concentration of compound achieving 50% rescue of the virus‐reduced eGFP signals as compared to the untreated virus‐infected control cells. Toxicity of compounds in the absence of virus was evaluated in a standard MTS‐assay as described previously [36].

#### 4.2.3 HCoV Screening

For the antiviral evaluation of the 2‐PhQs against HCoV‐OC43 and HCoV‐229E, HEL 299 cells were seeded into 384‐well dishes at 5,000 cells per well and incubated overnight at 37°C. Then, serial dilutions of the compounds were added to the cells prior to infection with HCoV‐229E at 30 CCID_50_ or with HCoV‐OC43 at 50 CCID_50_ per well. After 7 days incubation at 35°C, the virus‐induced cytopathogenic effect was measured colorimetrically by the formazan‐based 3‐(4,5‐dimethylthiazol‐2‐yl)‐5‐(3‐carboxymethoxyphenyl)‐2‐(4‐sulfophenyl)‐2H‐tetrazolium (MTS) cell viability assay (CellTiter 96 AQueous One Solution Cell Proliferation Assay from Promega, Madison, WI), and the antiviral activity was expressed as the 50% effective concentration (EC_50_). In parallel, the 50% cytotoxic concentration (CC_50_) was derived from mock‐infected cells.

#### 4.2.4 Determination of SARS‐CoV‐2 nsp13 Unwinding‐Associated Activity

The SARS‐CoV‐2 nsp13 unwinding‐associated activity was measured in black 384‐well plates (PerkinElmer), in a 40 μL reaction volume containing 20 mM Tris–HCl, pH 7.2, 50 mM NaCl, 2 μM Hel Capture oligo (5′‐ TGG TGC TCG AAC AGT GAC ‐3′) from Biomers, 5 mM MgCl_2_, 5% DMSO or inhibitor and 1 nM of purified nsp13. The reaction mixture containing the enzyme was preincubated for 10 min with the inhibitor at room temperature (RT). The reaction was started by adding 1 mM ATP and 750 nM annealed DNA substrate (5′‐ AGT CTT CTC CTG GTG CTC GAA CAG TGA C‐Cy3‐3′, 5′‐ BHQ‐2‐GTC ACT GTT CGA GCA CCA CCT CTT CTG A‐3′) from Biomers. After 15 min of incubation at 37°C, products were measured with Victor Nivo (Perkin) at 530/580 nm. Experiments were performed in triplicate. Compound SSYA10‐001 [26] was used as a positive control.

#### 4.2.5 Determination of SARS‐CoV‐2 nsp13 ATPase‐Associated Activity

The SARS‐CoV‐2 nsp13 ATPase helicase‐associated activity was measured in a transparent 96 well plate (PerkinElmer), in a 25 μL reaction volume containing 20 mM Tris–HCl, pH 7.2, 50 mM NaCl, 2 mM MgCl_2_, 5% DMSO or inhibitor, and 25 nM of purified nsp13. The reaction was started by adding 400 µM ATP. After 30 min of incubation at 37°C, 50 μL of Biomol Green Reagent (Prod. No. BML‐AK111, Enzo Lifescience) was added and the reaction was incubated for 10 min at RT, protected from the light. Products were measured with Victor Nivo (Perkin) at 650 nm.

#### 4.2.6 Determination of Kinetics of nsp13 ATPase Inhibition

The kinetics of inhibition of nsp13 ATPase activity were performed according to Corona et al.14 in a transparent 96 well plate (PerkinElmer), in 25 μL reaction volume containing 20 mM Tris–HCl, pH 7.2, 50 mM NaCl, 2 mM MgCl2, 5% DMSO or inhibitor and 25 nM of purified nsp13, titrating the ATP with final concentrations of 0.25, 0.12, 0.6, 0.3, and 0.1 mM. On the same plate, a calibration curve of inorganic phosphate (Phosphate Standard, Prod. No. BML‐KI102, Enzo Lifescience) was made, ranging from 800 to 0 µM. After 30 min of incubation at 37°C, 50 μL of Biomol Green Reagent (Prod. No. BML‐AK111, Enzo Lifescience) was added and the reaction was incubated for 10 min at RT, protected from the light. Products were measured with Victor Nivo (Perkin) at 650 nm. The pmols of inorganic phosphate produced were interpolated from the calibration curve. Results were plotted in a Lineweaver‐Burk graph as the reciprocal of pmol produced (*y* axes) versus the reciprocal of ATP concentration (*x* axes).

##### 4.2.6.1 Data Analysis

Data analysis of assay development results was performed using GraphPad Prism Version 9.1.2. Test compound results were normalized relative to respective controls. Dose response curves were fitted to a nonlinear regression of (log10) dose versus normalized response‐ variable slope. Assay quality was assessed using the Z′‐factor calculation with Z′ > 0.5 as a threshold for acceptance.

#### 4.2.7 Cytotoxicity

The cytotoxic profile of compounds 14 and 15 was evaluated on human bronchial epithelial cells (BEAS‐2B, ATCC CRL‐3588) and rat cardiomyoblasts (H9c2, ATCC CRL‐1446). BEAS‐2B cells were cultured in Airway Epithelial Cell Basal Medium (ATCC PCS‐300−030) supplemented with Bronchial Epithelial Cell Growth Kit (ATCC PCS‐300−040) and 100 unit/mL penicillin and 100 mg/mL streptomycin; 1% (Merck). H9c2 cells were maintained in DMEM (Merck) supplemented with 10% FBS, 100 unit/mL penicillin, and 100 mg/mL streptomycin; 1% (Merck). Both cell lines were maintained at 37°C in a humidified atmosphere of 5% CO2.

The toxicological evaluation of compounds **14** and **15** was conducted to investigate the reduction in cell viability and increase in ROS production after treatment with the selected compounds. For the evaluation of cell viability, cells at 90% confluence were used in the experiments. Cells were seeded onto a 96‐well clear‐bottom plate (10^4^ cells/well). After 24 h, the culture medium was removed and replaced with fresh medium containing either the vehicle (DMSO 0.33%) or the selected compounds (5; 10; 50; 100 μM) for 24 h. At the end of the incubation period, an aqueous solution of the soluble tetrazolium salt WST‐1 (Roche) was incubated for 1 h in an incubator at 37°C and 5% CO_2_. WST‐1 is a dehydrogenase enzyme substrate present in viable cells that converts this dye into a dark yellow product. After 1 h, cell viability was quantified using the microplate reader Victor Nivo (Revvity) at *λ *= 495 nm.

The evaluation of ROS production was evaluated on a 96‐well black plate (3 × 10^4^ cells/well) precoated with gelatin 1% (Merck). After 24 h, the culture medium was removed and replaced with a new medium containing either the vehicle (DMSO 0.33%) compound **14** and **15** (5; 10; 50; 100 μM) for 24 h. At the end of the treatment, the fluorescent probe dihydroethidium (DHE, 10 μM; Merck) was used to evaluate ROS production. It was solubilized in DMSO, diluted in HBSS (Merck), and incubated in the dark at 37°C for 30 min. During the incubation period, DHE freely permeates cell membranes and reacts with ROS species, particularly superoxide anions, forming a red fluorescent product (ethidium). Fluorescence was then evaluated using the microplate reader Victor Nivo (Revvity) at *λ*_ex_ = 500 nm and *λ*_em_ = 580 nm.

Cell viability (%) and ROS production (%) were calculated for each treatment, and the absorbance or fluorescence values were compared with those obtained in the vehicle‐treated cells (shown as 100%). The selected statistical analysis was one‐way ANOVA followed by Bonferroni's Multiple Comparison posthoc test. Values were considered statistically different for *p* < 0.05. Data was analyzed using the GraphPad Prism 8.0 software.

#### 4.2.8 Computational Studies

All computational studies were performed using Schrödinger Suite 2024–2. The X‐ray structure of SARS‐CoV‐2 helicase in complex with the ATP analog AMP‐PNP, deposited in the Protein Data Bank (PDB) under the code 7NN0 [16], was used as the starting point. The receptor structure was prepared following the same protocol as described in a previous study [22]. The synthesized library was built and prepared using the LigPrep tool [37]; hydrogens were added, salts were removed, and ionization states were calculated with Epik at pH 7.4. Docking studies were carried out with Glide using the SP (standard precision) algorithm [38], generating 10 poses per ligand for each identified binding site. Poses were ranked based on GlideScore, and the top‐scoring conformations were further analyzed for hydrogen bonding, *π*–*π*, and cation–*π* interactions with key catalytic and auxiliary residues. Interaction fingerprints were then compared across ligands to identify structural features correlating with enzymatic inhibition and antiviral activity*.*

#### 4.2.9 Pharmacokinetic Properties Determination: Determination of Solubility

Solubility of compound **15** was determined using the instrument Nepheloskan Ascent (Labsystems). The experiments were conducted at room temperature in 96‐multiwell in a final volume of 300 µL. Compound was tested in triplicate at different concentrations in PBS (pH 7.4) with different percentages of DMSO. The measurements were performed at 4 different times (*T*_0_, 30, 60, 120 min) from the preparation of the samples. Data obtained were compared to controls (PBS with DMSO) and the ratio of sample/control was determined.

The compounds are considered soluble if the ratio is ≤3.

#### 4.2.10 In Vitro Membrane Permeability and Metabolism

Donor solutions (250 µM for reference compounds, 100 µM for **15**) were prepared from DMSO stock solutions diluted with 1× PBS, pH 7.4, 50% DMSO final concentration. The artificial lipid membrane solutions for PAMPA were prepared by dissolving phosphatidylcholine in dodecane at 1% (w/v). The 96‐well microfilter plates (MultiScreen‐IP, 0.45 µm, catalog no. MAIPN4550) and the 96‐well microtiter plate (MultiScreen‐acceptor, catalog no. MSSACCEPTOR) were used as donor and acceptor compartments. Filters of donor plates were coated with 5 µL of freshly prepared artificial membrane solutions. After applying the lipids, acceptor plate wells were charged with 300 µL of 1 × PBS, pH 7.4, 50% DMSO solution. Donor plates were charged with 150 µL of donor solutions and placed onto the acceptor plates. The resulting sandwich was incubated at room temperature for 2 h under stirring on Heidolph TITRAMAX 100 (600 rpm). Then, the sandwich plates were separated, and concentrations of the compounds in the acceptor wells were determined by UV analyses.

All compounds were assayed in triplicate, and the apparent (*P*_app_, Log*P*_app_) permeability values were reported as mean ± standard deviation. The apparent permeability value, *P*_app_, was calculated based on the Faller modification of the Sugano Equation (1) [31]:

(1) $$P_{\mathrm{app}}=-\left[\frac{\left(V_{D}V_{R}\right)}{\left(V_{D}+V_{R}\right)}*At\right]*\ln\;(1-r)$$

*V*_R_ is the volume of the acceptor compartment (0.300 cm^3^), *V*_D_ is the donor volume (0.150 cm^3^), *A* is the accessible filter area (0.266 cm^2^), *t* is the incubation time in seconds, and *r* is the ratio of acceptor and equilibrium solution concentrations.

##### 4.2.10.1 Liver Microsomal Stability

###### 4.2.10.1.1 Instrumentation and LC‐MS/MS Conditions

Analyses were performed on a Waters ACQUITY UPLC I‐Class system coupled online to a Waters Xevo TQ‐XS triple‐quadrupole mass spectrometer (Waters Technologies Corp., Milford, MA, USA) equipped with an ESI source operating in positive ion mode.

The chromatographic separation was performed on a Kinetex Evo C8 column (50 × 3 mm, 2.6 μm, 100 Å; Phenomenex, Bologna, Italy) using H_2_O (A) and ACN (B) as mobile phases, both acidified with 0.1% v/v HCOOH. The gradient program was as follows: 0.01–3.00 min, 5%–95% B; 3.01–4.00 min, isocratic at 95% B; followed by 2.50 min for column re‐equilibration. The flow rate of mobile phases and column oven was set at 0.7 mL min^−1^ and 40°C, respectively.

The multiple reaction monitoring (MRM) transition was optimized at m/z 559.3 > 220.2. The following mass spectrometer parameters were applied: source temperature, 150°C; desolvation temperature, 650°C; transfer capillary voltage, 4.0 kV; cone, 30 V; flow rate of cone gas (nitrogen) 150 L/h; desolvation gas flow rate, 1100 L/h; collision gas, argon; collision energy, 26 Ev. Data was obtained by MassLynx V4.2 SCN 1045 software and further calibrated and quantified by TargetLynx XS V4.2 SCN 1045.

For calibration, a stock solution was prepared in DMSO and serially diluted in methanol containing 0.1% TFA to obtain concentrations from 0.0025 to 1 µM. Tolbutamide (1 µM) was used as the internal standard (IS). Calibration curves, built from six concentration levels (each in triplicate), showed good linearity (*R* ^2^ ≥ 0.99) with the regression equation *y* = 0.20385*x*–0.00552.

##### 4.2.10.2 Microsomal Stability

The liver microsomal stability assay was conducted as previously described [39]. Briefly, HLMs and MLMs (Thermo Fisher Scientific, Bremen, Germany) were used, and the reaction was initiated by the addition of *β*‐nicotinamide adenine dinucleotide phosphate (NADPH). The incubation was carried out at 37°C for 20, 40, and 60 min in a Thermomixer comfort (Eppendorf, Hamburg, Germany). The reaction was quenched with ice‐cold methanol containing IS, and the samples were centrifuged. The resulting supernatants were then injected into the LC‐MS system. The 0 min control was obtained by adding methanol immediately after incubation. Testosterone served as a positive control, while the negative control consisted of incubation without a cofactor for up to 60 min. All experiments were performed in triplicate. Results were expressed as microsomal half‐life (*t*_1/2_), in vitro microsomal intrinsic clearance (CL_int, micr_), and in vivo intrinsic hepatic clearance (CL_int_). The *t*_1/2_ was calculated as 0.693/*b*, where *b* is the slope of the linear regression of ln(fraction remaining) versus time. The in vitro microsomal intrinsic clearance was calculated as CL_int, micr _= (1000 × 0.693/*t*_1/2_)/0.5, and then scaled to CL_int_ using the physiology‐based scaling factor [40].

##### 4.2.10.3 Glutathione (GSH) Trapping Assay

The trapping assay was performed under the same conditions as the liver metabolic stability assay, except for the inclusion of NADPH and GSH solutions in a 1:1 volume ratio.

After 60 min of incubation with liver microsomes at 37°C under shaking at 450 rpm, the reaction was quenched by adding 100 μL of H_2_O and 600 μL of ice‐cold acetonitrile:methanol (9:1, v/v) containing IS. After quenching the reaction, the samples were centrifuged and the supernatants were evaporated to approximately 200 μL under a gentle nitrogen stream at 35°C (Concentrator plus, Eppendorf, Hamburg, Germany) prior to LC‐MS analysis [41]. Positive control samples included diclofenac and acetaminophen.

##### 4.2.10.4 Metabolite Identification

LC‐MS/MS analysis was performed on a Vanquis UHPLC system connected online to an Orbitrap Exploris 120 mass spectrometer (Thermo Fisher Scientific, Bremen, Germany) equipped with a heated ESI probe (HESI II).

The chromatographic separation was performed on a Kinetex 2.6 μm Evo C18 column (100 × 2.1 mm, Phenomenex) using H_2_O (A) and ACN (B), both acidified with 0.1% formic acid, with the following gradient: 0.01–10.00 min, 5%–95% B; isocratic at 95% B for 1 min; 11.01–11.50 min, 95%–5% B; followed by 1.5 min for re‐equilibration. The flow rate and column temperature were set at 0.4 mL/min and 40°C, respectively.

Full MS parameters: Orbitrap resolution 60,000; scan range (m/z) 100–1500; RF lens 70%; AGC target 200%; maximum injection time 200 ms. Data‐dependent MS/MS: Orbitrap resolution 15,000; isolation window 2 m/z; normalized HCD collision energy 30%. Ion source parameters: sheath gas 60; auxiliary gas 15; sweep gas 2; ion transfer tube and vaporizer temperature 300°C; spray voltage +3.4 kV/−3.0 kV.

The evaluation of the cytotoxic profile of compounds **14** and **15** was carried out on human bronchial epithelial cells (BEAS‐2B, ATCC CRL‐3588) and rat cardiomyoblasts (H9c2, ATCC CRL‐1446). BEAS‐2B were cultured in Airway Epithelial Cell Basal Medium (ATCC PCS‐300−030) supplemented with Bronchial Epithelial Cell Growth Kit (ATCC PCS‐300−040) and 100 unit/mL penicillin and 100 mg/mL streptomycin; 1% (Merck) while H9c2 were maintained in DMEM (Merck) supplemented with 10% FBS, 100 unit/mL penicillin and 100 mg/mL streptomycin; 1% (Merck). Both cell lines were maintained at 37°C in a humidified atmosphere of 5% CO_2_.

## Supporting Information

Additional supporting information can be found online in the Supporting Information section. **Supporting Fig. S1:** ^1^H NMR (400 MHz, CDCl_3_) spectrum of compound **3**. **Supporting Fig. S2:** ^13^C NMR (101 MHz, CDCl_3_) spectrum of compound **3**. **Supporting Fig. S3:** ^1^H NMR (400 MHz, CDCl_3_) spectrum of compound **4**. **Supporting Fig. S4:** ^13^C NMR (101 MHz, CDCl_3_) spectrum of compound **4**. **Supporting Fig. S5:** ^1^H NMR (400 MHz, CDCl_3_) spectrum of compound **5**. **Supporting Fig. S6:** ^13^C NMR (101 MHz, CDCl_3_) spectrum of compound **5**. **Supporting Fig. S7:** ^1^H NMR (400 MHz, CDCl_3_) spectrum of compound **6**. **Supporting Fig. S8:** ^13^C NMR (101 MHz, CDCl_3_) spectrum of compound **6**. **Supporting Fig. S9:** ^1^H NMR (400 MHz, CDCl_3_) spectrum of compound **7**. **Supporting Fig. S10:** ^13^C NMR (101 MHz, CDCl_3_) spectrum of compound **7**. **Supporting Fig. S11:** ^1^H NMR (400 MHz, CDCl_3_) spectrum of compound **8**. **Supporting Fig. S12:** ^13^C NMR (101 MHz, CDCl_3_) spectrum of compound **8**. **Supporting Fig. S13:** ^1^H NMR (400 MHz, CDCl_3_) spectrum of compound **9**. **Supporting Fig. S14:** ^13^C NMR (101 MHz, CDCl_3_) spectrum of compound **9**. **Supporting Fig. S15:** ^1^H NMR (400 MHz, CDCl_3_) spectrum of compound **10**. **Supporting Fig. S16:** ^13^C NMR (101 MHz, CDCl_3_) spectrum of compound **10**. **Supporting Fig. S17:** ^1^H NMR (400 MHz, CDCl_3_) spectrum of compound **11**. **Supporting Fig. S18:** ^13^C NMR (101 MHz, CDCl_3_) spectrum of compound **11**. **Supporting Fig. S19:** ^1^H NMR (400 MHz, CDCl_3_) spectrum of compound **12**. **Supporting Fig. S20:** ^13^C NMR (101 MHz, CDCl_3_) spectrum of compound **12**. **Supporting Fig. S21:** ^1^H NMR (400 MHz, CDCl_3_) spectrum of compound **13**. **Supporting Fig. S22:** ^13^C NMR (101 MHz, CDCl_3_) spectrum of compound **13**. **Supporting Fig. S23:** ^1^H NMR (400 MHz, CDCl_3_) spectrum of compound **14**. **Supporting Fig. S24:** ^13^C NMR (101 MHz, CDCl_3_) spectrum of compound **14**. **Supporting Fig. S25:** ^1^H NMR (400 MHz, CDCl_3_) spectrum of compound **15**. **Supporting Fig. S26:** ^13^C NMR (101 MHz, CDCl_3_) spectrum of compound **15**. **Supporting Fig. S27:** ^1^H NMR (400 MHz, CDCl_3_) spectrum of compound **16**. **Supporting Fig. S28:** ^13^C NMR (101 MHz, CDCl_3_) spectrum of compound **16**. **Supporting Fig. S29:** ^1^H NMR (400 MHz, CDCl_3_) spectrum of compound **17**. **Supporting Fig. S30:** ^13^C NMR (101 MHz, CDCl_3_) spectrum of compound **17**. **Supporting Fig. S31:** ^1^H NMR (400 MHz, CDCl_3_) spectrum of compound **18**. **Supporting Fig. S32:** ^13^C NMR (101 MHz, CDCl_3_) spectrum of compound **18**. **Supporting Fig. S33:** ^1^H NMR (400 MHz, CDCl_3_) spectrum of compound **19**. **Supporting Fig. S34:** ^13^C NMR (101 MHz, CDCl_3_) spectrum of compound **19**. **Supporting Fig. S35:** ^1^H NMR (400 MHz, CDCl_3_) spectrum of compound **20**. **Supporting Fig. S36:** ^13^C NMR (101 MHz, CDCl_3_) spectrum of compound **20**. **Supporting Fig. S37:** ^1^H NMR (400 MHz, CDCl_3_) spectrum of compound **21**. **Supporting Fig. S38:** ^13^C NMR (101 MHz, CDCl_3_) spectrum of compound **21**. **Supporting Fig. S39:** ^1^H NMR (400 MHz, DMSO‐*d*_6_) spectrum of compound **22**. **Supporting Fig. S40:** ^13^C NMR (101 MHz, DMSO‐*d*_6_) spectrum of compound **22**. **Supporting Fig. S41:** ^1^H NMR (400 MHz, DMSO‐*d*_6_) spectrum of compound **23**. **Supporting Fig. S42:** ^13^C NMR (101 MHz, DMSO‐*d*_6_) spectrum of compound **23**. **Supporting Fig. S43:** ^1^H NMR (400 MHz, CDCl_3_) spectrum of compound **24**. **Supporting Fig. S44:** ^13^C NMR (101 MHz, CDCl_3_) spectrum of compound **24**. **Supporting Fig. S45:** ^1^ H NMR (101 MHz, CDCl_3_) spectrum of compound **25**. **Supporting Fig. S46:** ^13^C NMR (101 MHz, CDCl_3_) spectrum of compound **25**. **Supporting Fig. S47:** ^1^H NMR (400 MHz, CDCl_3_) spectrum of compound **26**. **Supporting Fig. S48:** ^13^C NMR (101 MHz, CDCl_3_) spectrum of compound **26**. **Supporting Fig. S49:** ^1^ H NMR (400 MHz, CDCl_3_) spectrum of compound **28**. **Supporting Fig. S50:** ^13^C NMR (101 MHz, CDCl_3_) spectrum of compound **28**. **Supporting Fig. S51:** ^1^ H NMR (400 MHz, DMSO‐*d*_6_) spectrum of compound **29**. **Supporting Fig. S52:** ^13^C NMR (101 MHz, DMSO‐*d*_6_) spectrum of compound **29**. **Supporting Fig. S53:** HPLC of compound **4**. **Supporting Fig. S54:** HPLC of compound **14**. **Supporting Fig. S55:** HPLC of compound **15**. **Supporting Fig. S56:** HPLC of compound **26**. **Supporting Fig. S57:** HPLC of compound **29**. **Supporting Fig. S58:** Evaluation of cell viability and ROS production of BEAS‐2B cells after 24h incubation with vehicle (DMSO 0.33%), 14 (A, B) or 15 (C, D) (5; 10; 50; 100 μM). Data are expressed as mean ± SEM. * indicates significant statistical difference vs Vehicle (* p < 0.05, ** p < 0.01**** p < 0.0001). **Supporting Fig. S59:** Evaluation of cell viability and ROS production of H9c2 cells after 24h incubation with vehicle (DMSO 0.33%) **14** (A, B) or **15** (C, D) (5; 10; 50;100 μM). Data are expressed as mean ± SEM. * indicates significant statistical difference vs Vehicle (* p < 0.05, *** p < 0.001; **** p < 0.0001). **Supporting Fig. S60:** Solubility of **15** in PBS buffer + 1% DMSO. **Supporting Fig. S61:** Time‐dependent disappearance of compound **15** following incubation with human (**A**, HLMs) and mouse (**B**, MLMs) liver microsomes. Extracted ion chromatograms (XICs) of the corresponding metabolites obtained after 60 min of incubation (**C**: HLMs; **D**: MLMs). **Supporting Fig. S62:** LC‐MS profiles of the identified metabolites M1 (**A**) and M2 (**B**) at different incubation time points with liver microsomes. MS/MS spectra illustrating the fragmentation pathways of metabolites M1 (**C**) and M2 (**D**) derived from compound **15**. **Supporting Fig. S63:** XICs of metabolites M3 (**A**) and M4 (**B**), and corresponding MS^2^ spectra (**C**: M3; **D**: M4) following liver biotransformation of compound **15**. **Supporting Table S1:** Apparent permeability value of **15**.

## Funding

This study was supported by NextGenerationEU‐MUR PNRR (INF‐ACT Cascade Open Call 2023;COC‐1‐ 2023‐CNR), PRIN 2022 (20228NPP2Y), and Herculesstichting (ZW13−02).

## Conflicts of Interest

The authors declare no conflicts of interest.

## Supporting information

Supplementary Material

## Data Availability

The data that support the findings of this study are available in the supplementary material of this article.
