# Supplementary material for: 2‐Phenylquinolines Exhibit Anti‐Severe Acute Respiratory Syndrome Coronavirus‐2 Activity Through the Nonstructural Protein 13 Helicase Inhibition
Source: ChemMedChem. 2026 Apr 10;21(7):e202501063. doi: 10.1002/cmdc.202501063 (PMC13068633; doi:10.1002/cmdc.202501063)
Supplement: Supplementary file 1 — Supplementary Material [file CMDC-21-e202501063-s001.pdf]

2-Phenylquinolines exhibit anti-SARS-CoV-2 activity through the Nsp13 helicase inhibition

Authors

Giada Cernicchi,[1+] Maria Giulia Nizi,[1+] Roberta Emmolo[2], Leentje Persoons,[3] Manon Laporte,[4] Dirk Jochmans,[4] Isabella Romeo,[5] Giacomo Pepe,[6] Ciro Milite,[6] Pietro Campiglia,[6] Jacopo Spezzini,[7] Alma Martelli,[7] Simone Brogi,[7] Francesca Esposito,[2] Tommaso Felicetti,[1] Serena Massari,[1] Giuseppe Manfroni,[1] Stefano Sabatini,[1] Gianluca Sbardella,[6] Stefano Alcaro,[5] Johan Neyts,[4] Angela Corona,[2] Enzo Tramontano,[2] Steven De Jonghe,[8]* and Oriana Tabarrini[1]*

Affiliations

[1] Department of Pharmaceutical Sciences, University of Perugia, Via del Liceo, 1, 06123, Perugia, Italy
[2] Department of Life and Environmental Sciences, University of Cagliari, Cittadella Universitaria di Monserrato, Cagliari, Italy
[3] KU Leuven, Department of Microbiology, Immunology and Transplantation, Rega Institute for Medical Research, Molecular Genetics and Therapeutics in Virology and Oncology Research Group, Herestraat 49, box 1048, 3000 Leuven, Belgium.
[4] KU Leuven, Department of Microbiology, Immunology and Transplantation, Rega Institute for Medical Research, Virology, Antiviral Drug & Vaccine Research Group, Herestraat 49, box 1043, 3000 Leuven, Belgium.
[5] Department of Health Sciences, University of "Magna Græcia" of Catanzaro, Campus "S. Venuta", Viale Europa, 88100, Catanzaro, Italy
[6] Department of Pharmacy, University of Salerno, Via Giovanni Paolo II, 132, 84084 Fisciano, Italy
[7] Department of Pharmacy, University of Pisa, via Bonanno, 6, Pisa 56126, Italy
[8] KU Leuven, Department of Microbiology, Immunology and Transplantation, Rega Institute for Medical Research, Molecular, Structural and Translational Virology Research Group, Herestraat 49, box 1049, 3000 Leuven, Belgium.

[+] These authors contributed

* Corresponding authors email OT: oriana.tabarrini@unipg.it; email SDJ: steven.dejonghe@kuleuven.be

Supporting information

1. **Chemistry**
   - 1.1. ^{1}H NMR and ^{13}C NMR spectra of target compounds — p. 1-26
   - 1.2. HPLC analysis of exemplary target compounds — p. 27-29
2. **Biology**
   - 2.1. Cytotoxicity profiling — p. 29-30
   - 2.2. Preliminary In Vitro PK Evaluation of compound **15** — p.30-33

1. **Chemistry**

1.1. ^{1}H NMR and ^{13}C NMR spectra of target compounds

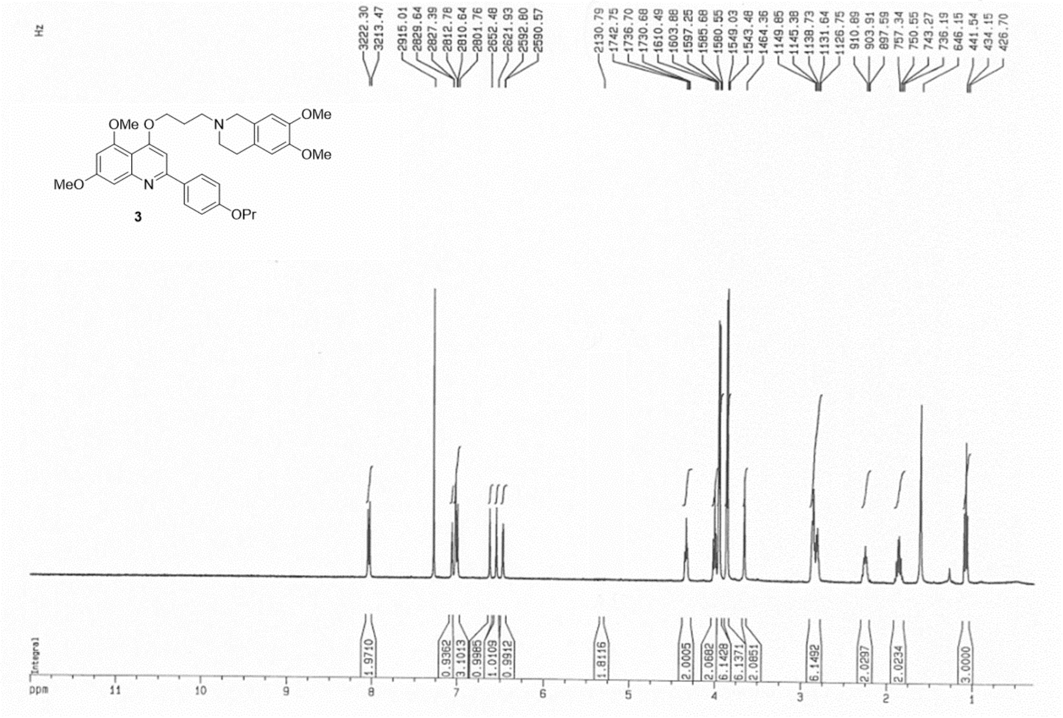

Figure S1. ^{1}H NMR (400 MHz, $CDCl_3$) spectrum of compound **3**.

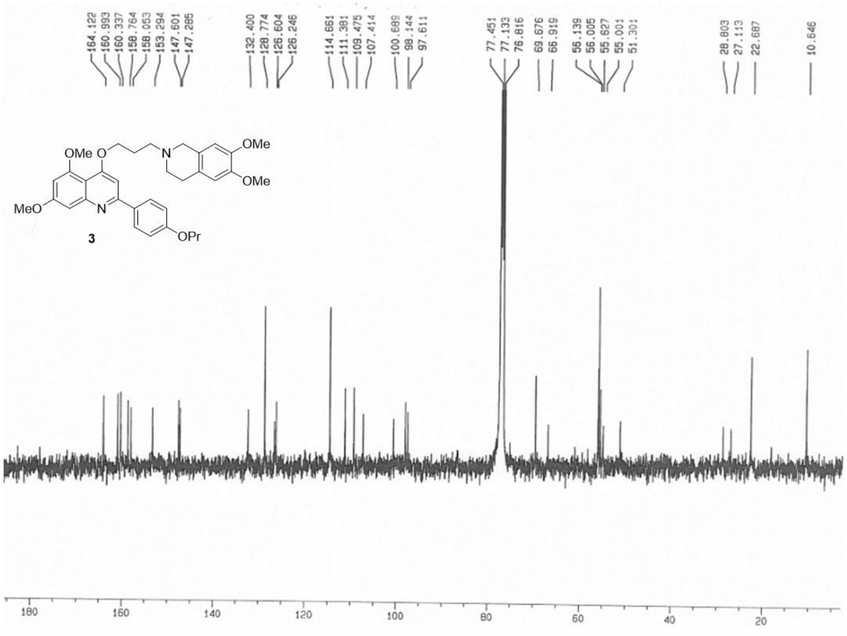

Figure S2. ^{13}C NMR (101 MHz, $CDCl_3$) spectrum of compound **3**.

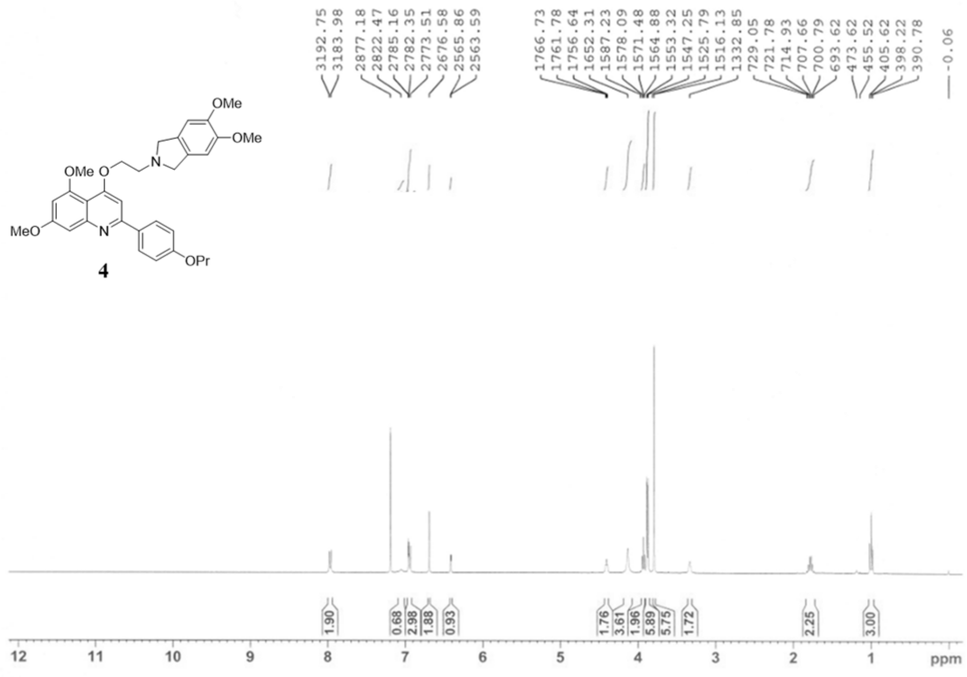

Figure S3. ^{1}H NMR (400 MHz, $CDCl_3$) spectrum of compound **4**.

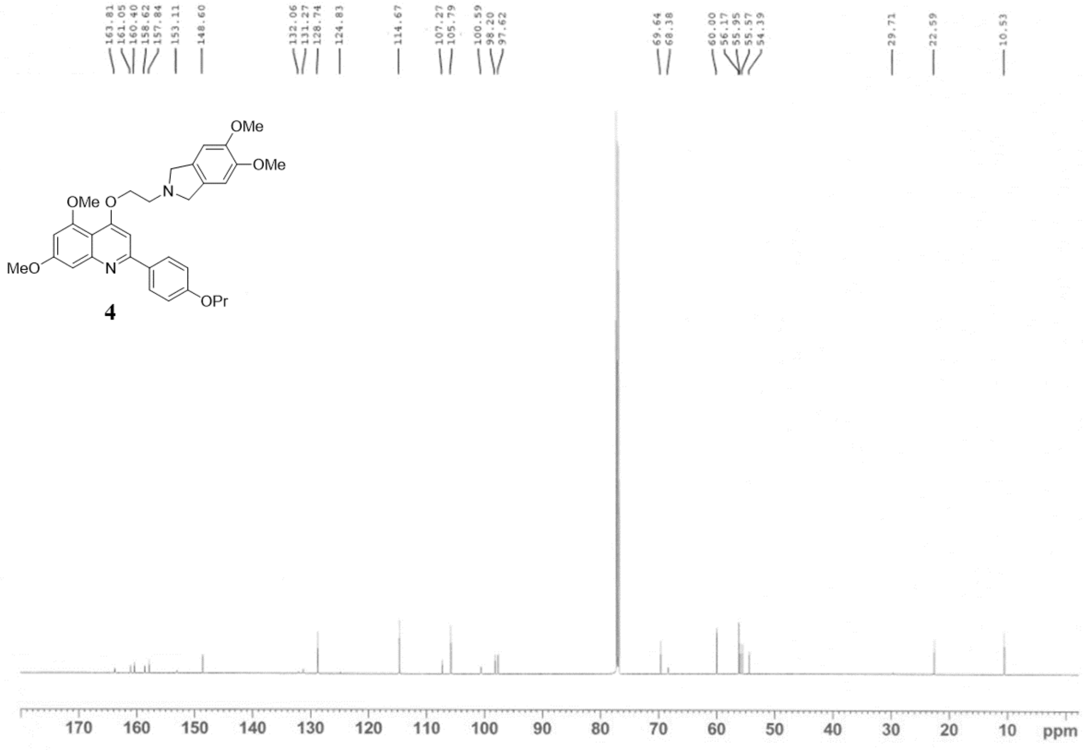

Figure S4. ^{13}C NMR (101 MHz, $CDCl_3$) spectrum of compound **4**.

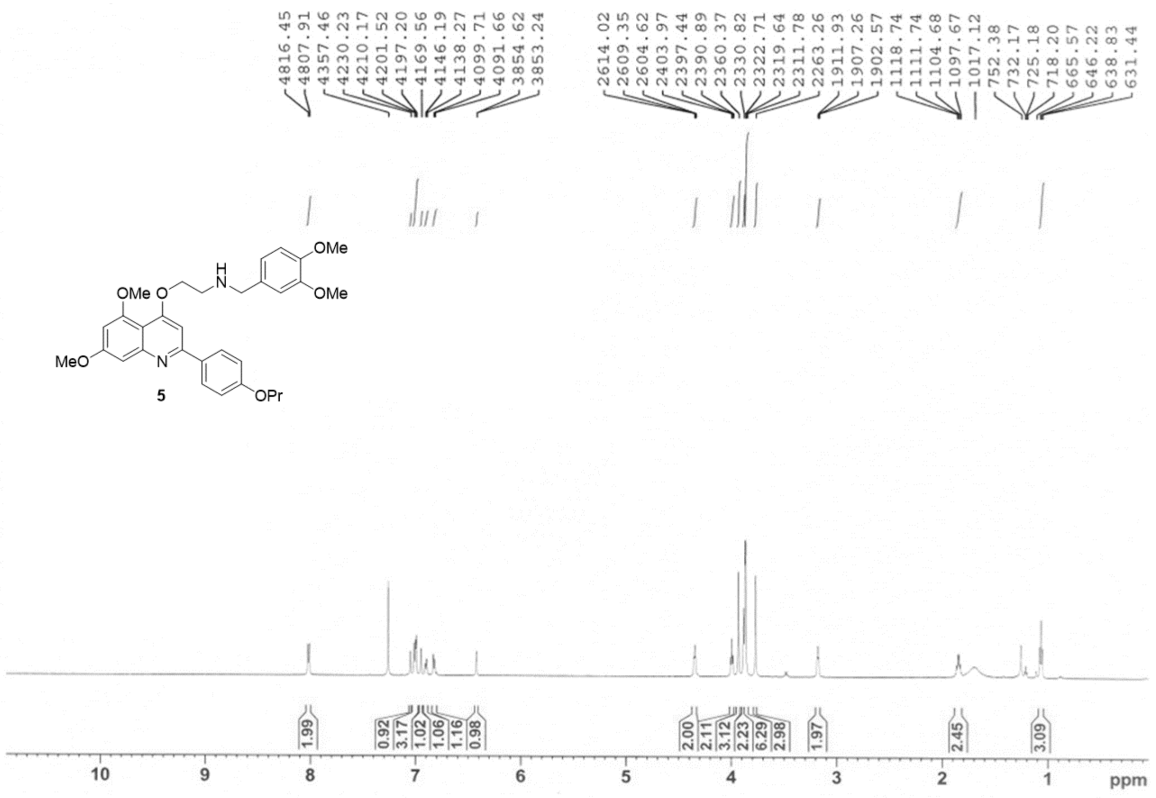

Figure S5. ^{1}H NMR (400 MHz, $CDCl_3$) spectrum of compound **5**.

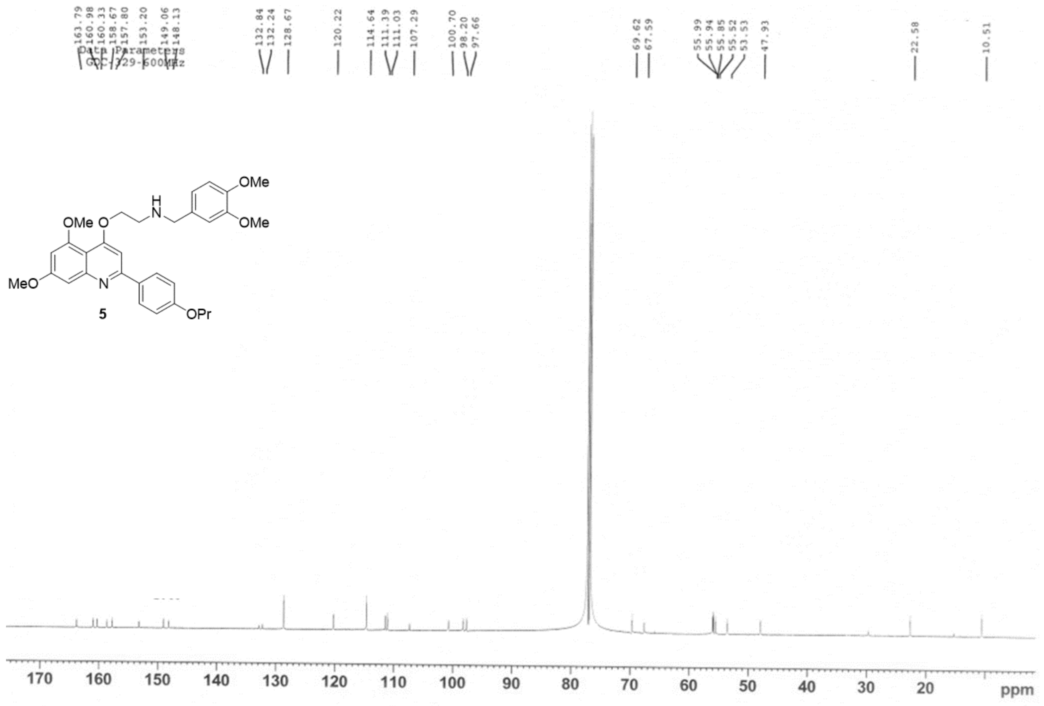

Figure S6. ^{13}C NMR (101 MHz, $CDCl_3$) spectrum of compound **5**.

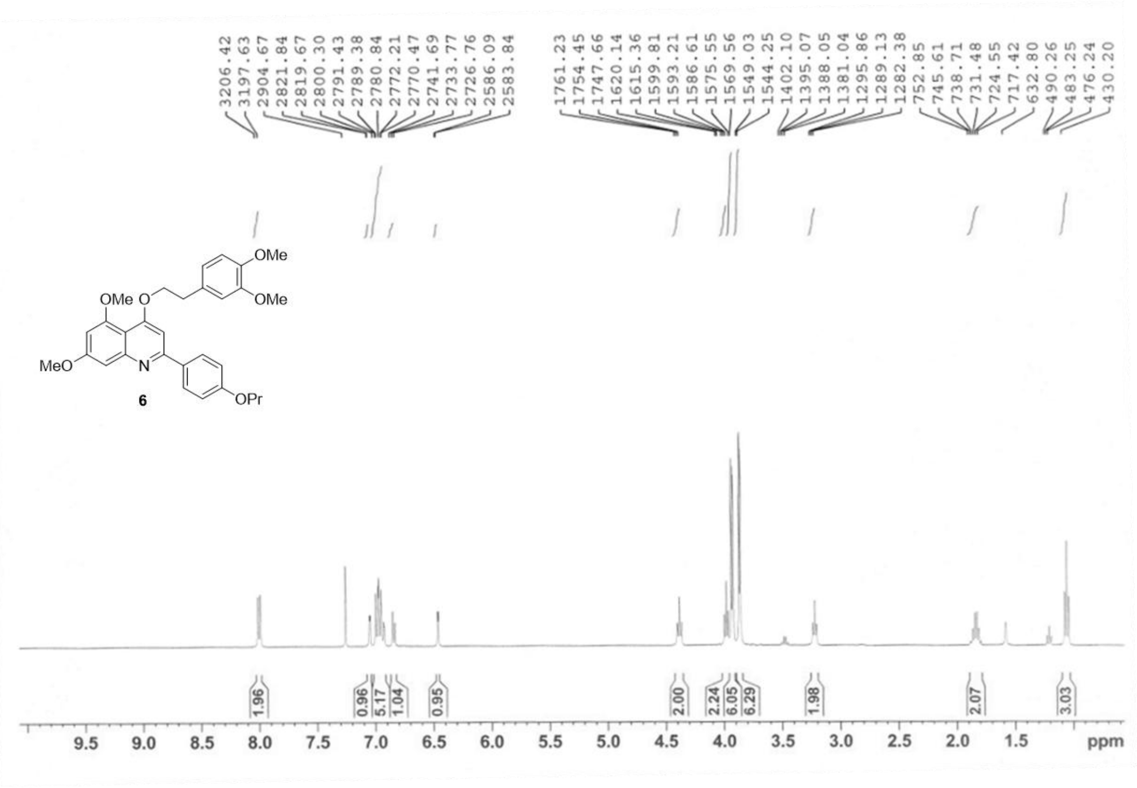

Figure S7. ^{1}H NMR (400 MHz, $CDCl_3$) spectrum of compound **6**.

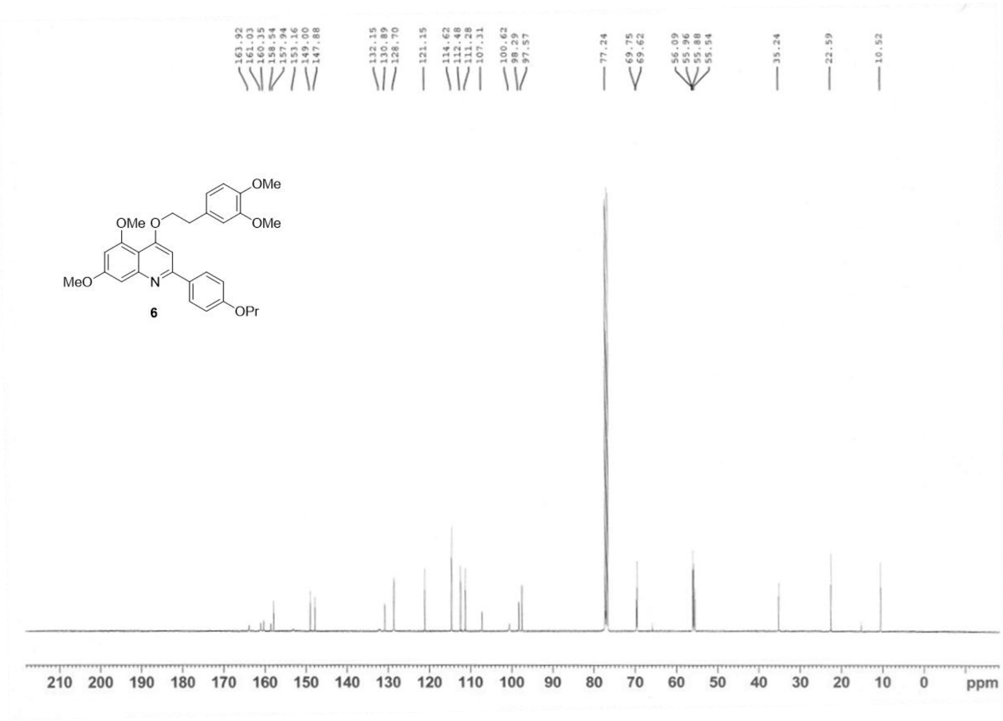

Figure S8. ^{13}C NMR (101 MHz, $CDCl_3$) spectrum of compound **6**.

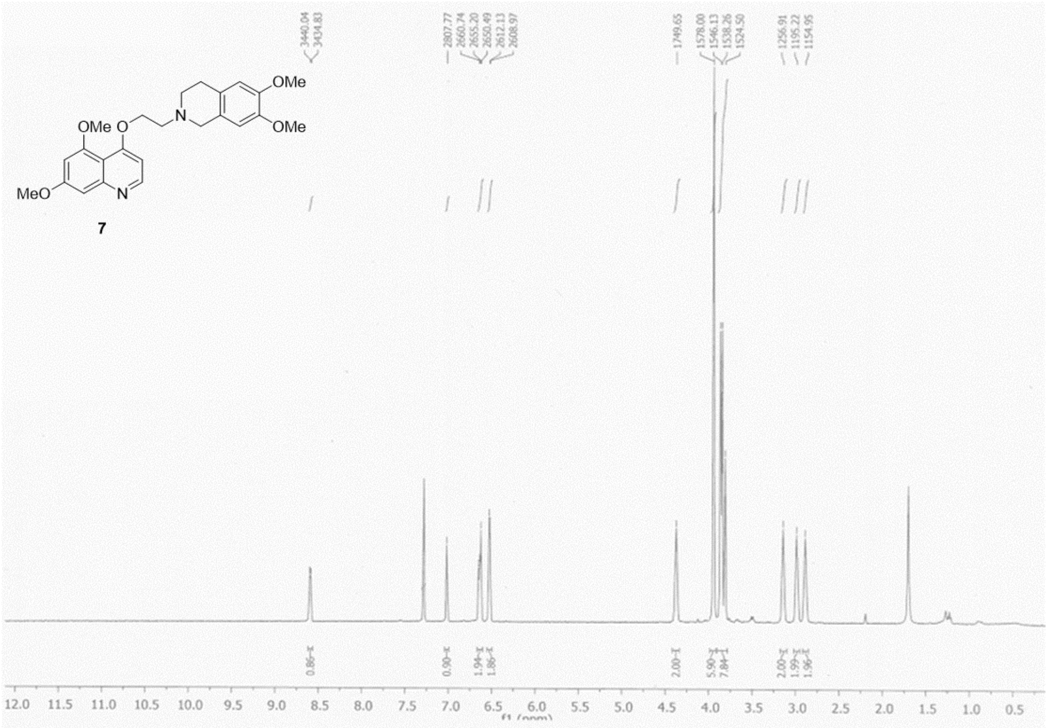

Figure S9. ^{1}H NMR (400 MHz, $CDCl_3$) spectrum of compound **7**.

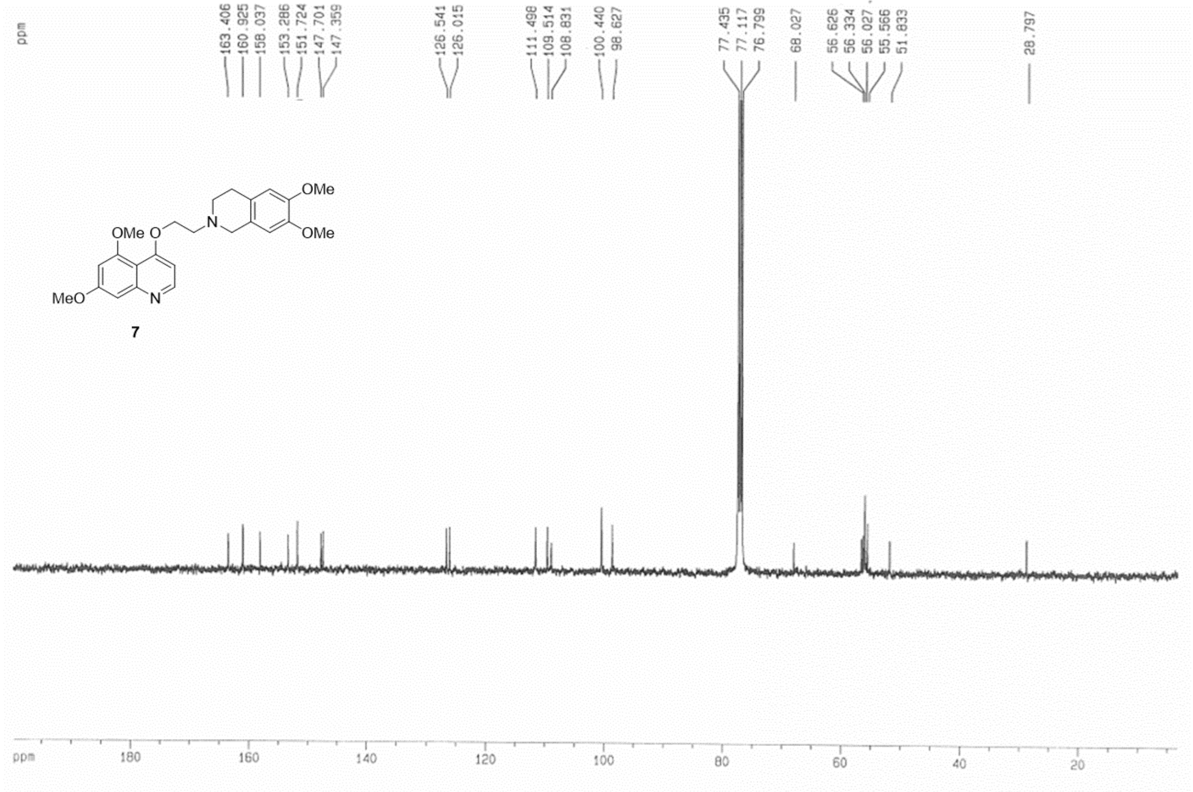

Figure S10. ^{13}C NMR (101 MHz, $CDCl_3$) spectrum of compound **7**.

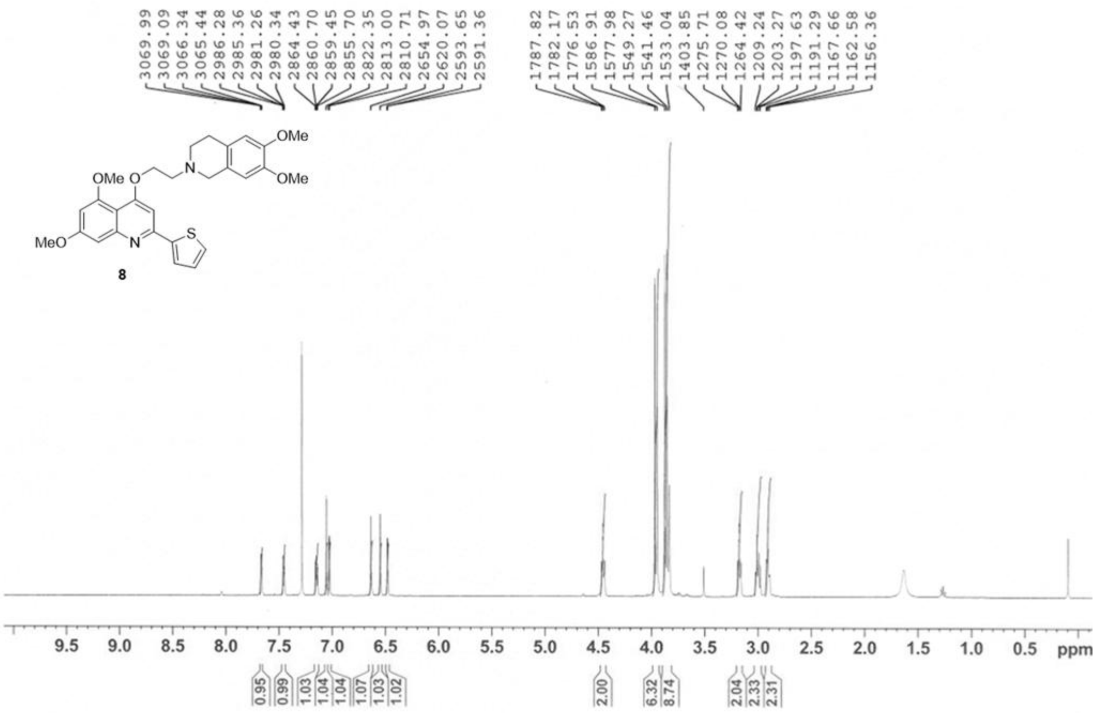

Figure S11. ^{1}H NMR (400 MHz, $CDCl_3$) spectrum of compound **8**.

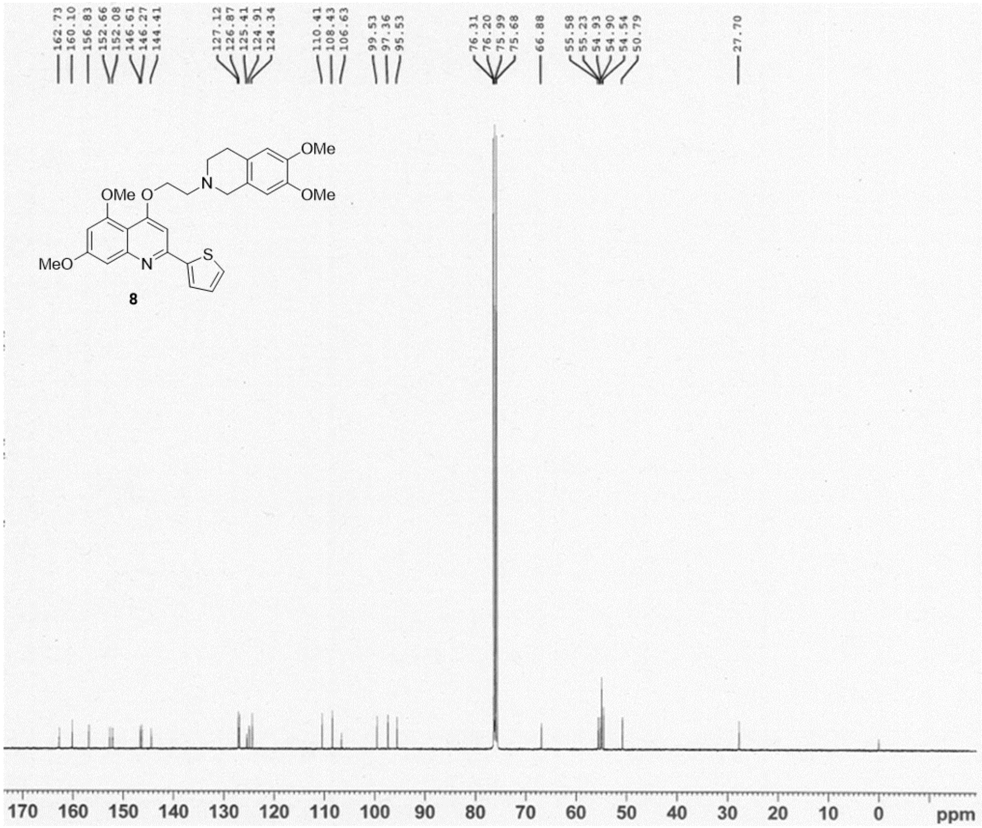

Figure S12. ^{13}C NMR (101 MHz, $CDCl_3$) spectrum of compound **8**.

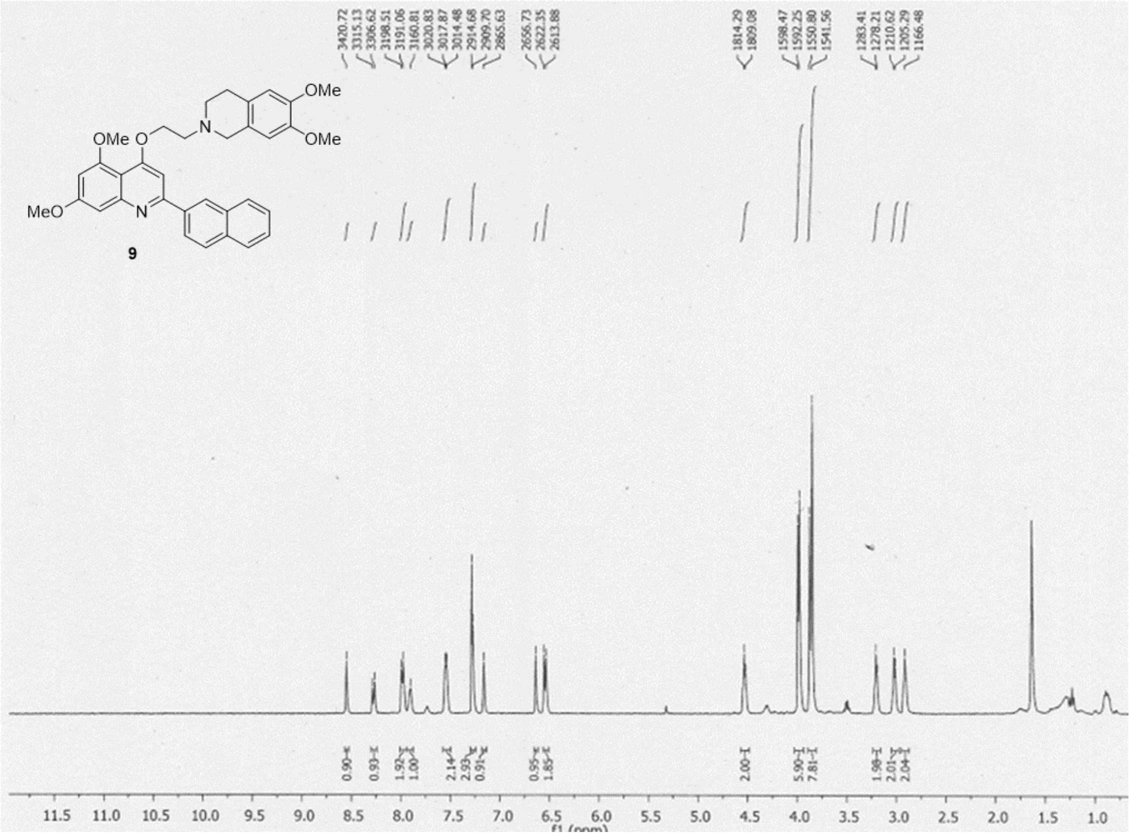

Figure S13. ^{1}H NMR (400 MHz, $CDCl_3$) spectrum of compound **9**.

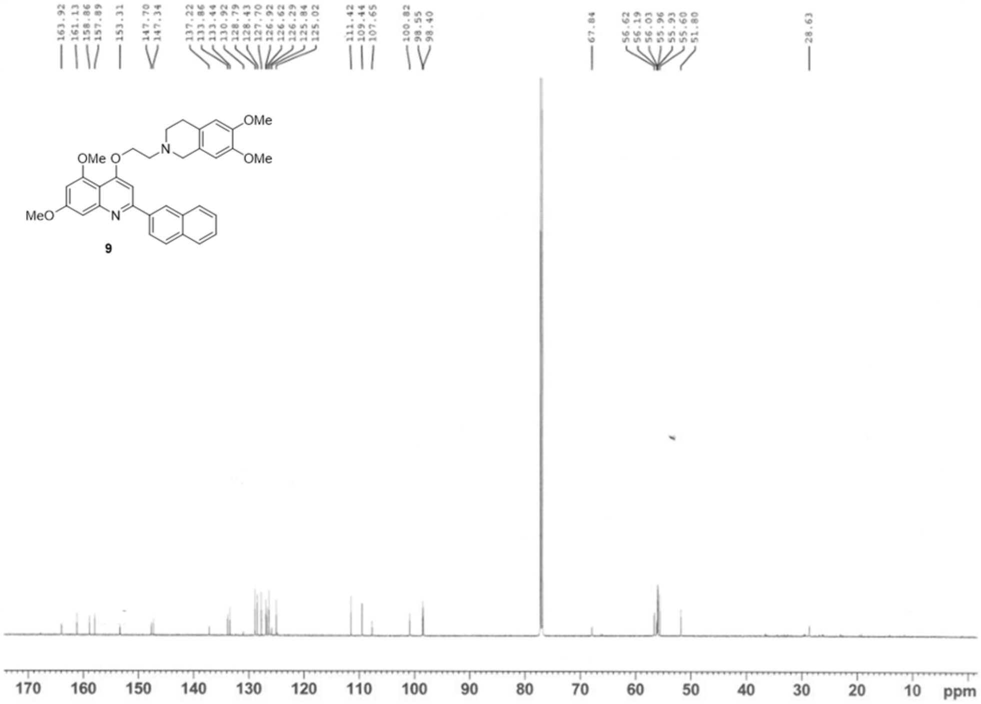

Figure S14. ^{13}C NMR (101 MHz, $CDCl_3$) spectrum of compound **9**.

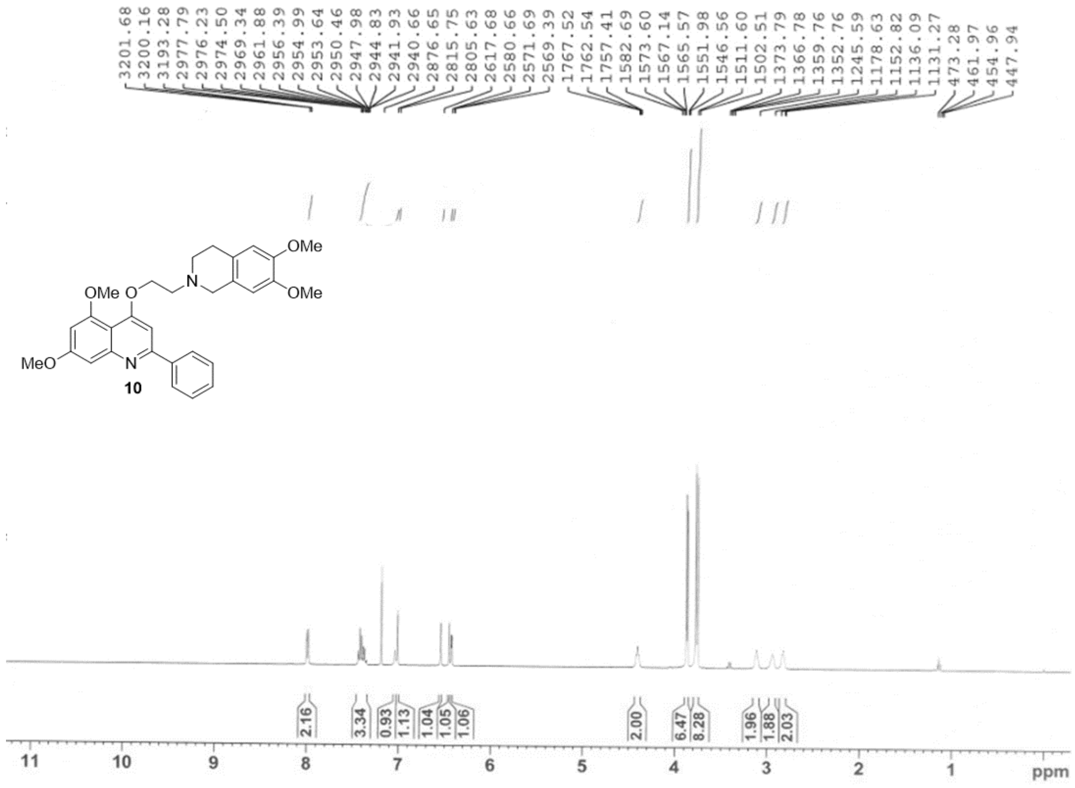

Figure S15. ^{1}H NMR (400 MHz, $CDCl_3$) spectrum of compound **10**.

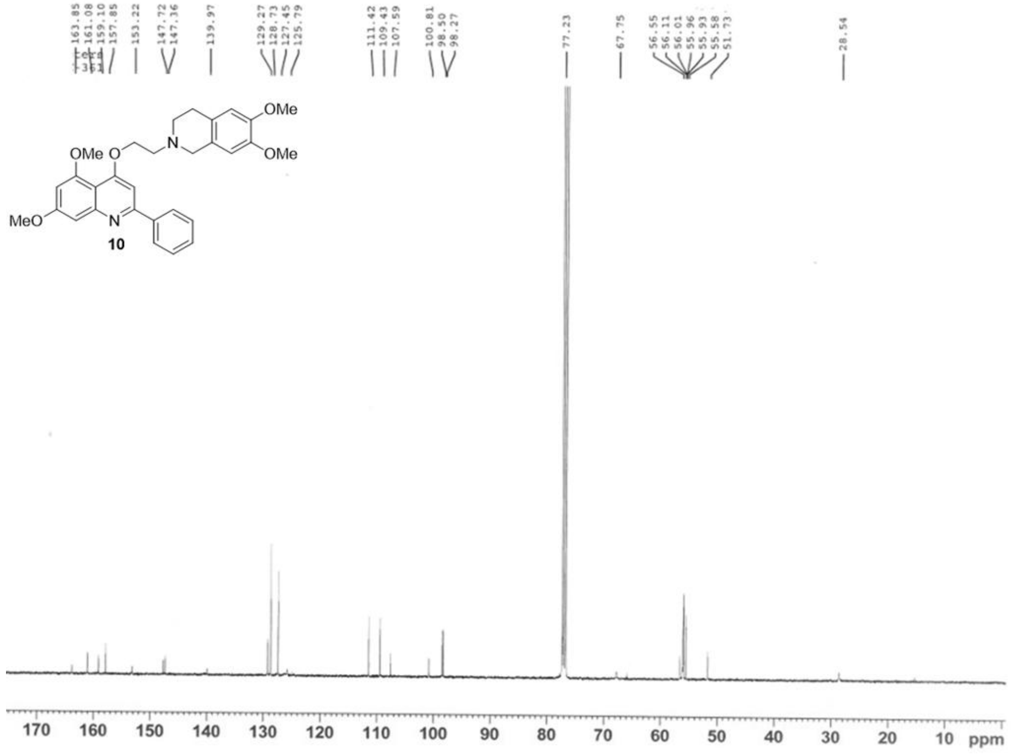

Figure S16. ^{13}C NMR (101 MHz, $CDCl_3$) spectrum of compound **10**.

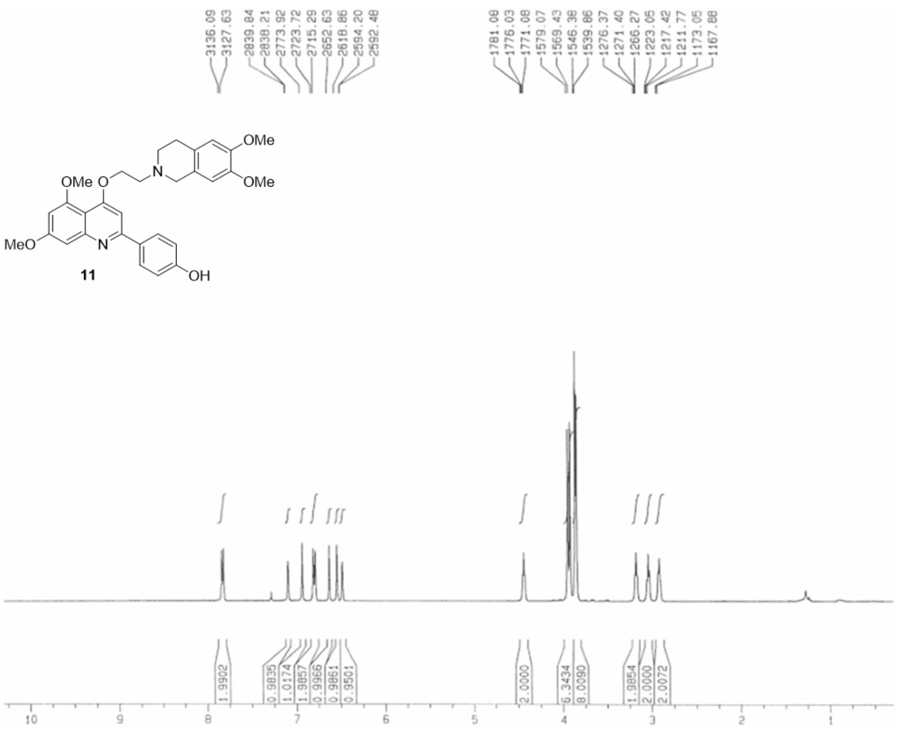

Figure S17. ^{1}H NMR (400 MHz, $CDCl_3$) spectrum of compound **11**.

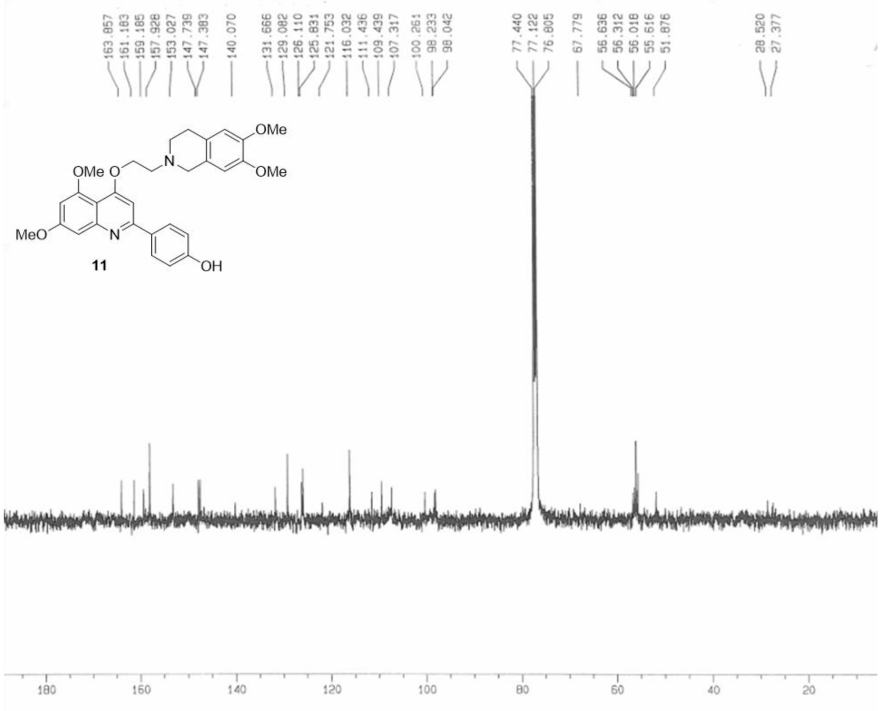

Figure S18. ^{13}C NMR (101 MHz, $CDCl_3$) spectrum of compound **11**.

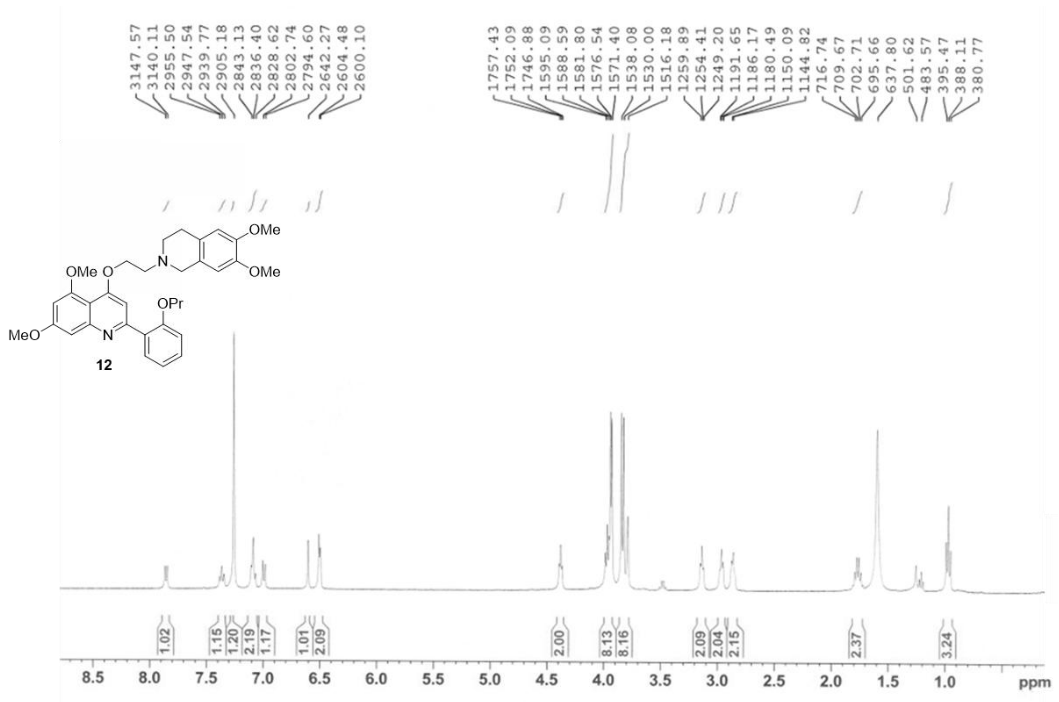

Figure S19. ^{1}H NMR (400 MHz, $CDCl_3$) spectrum of compound **12**.

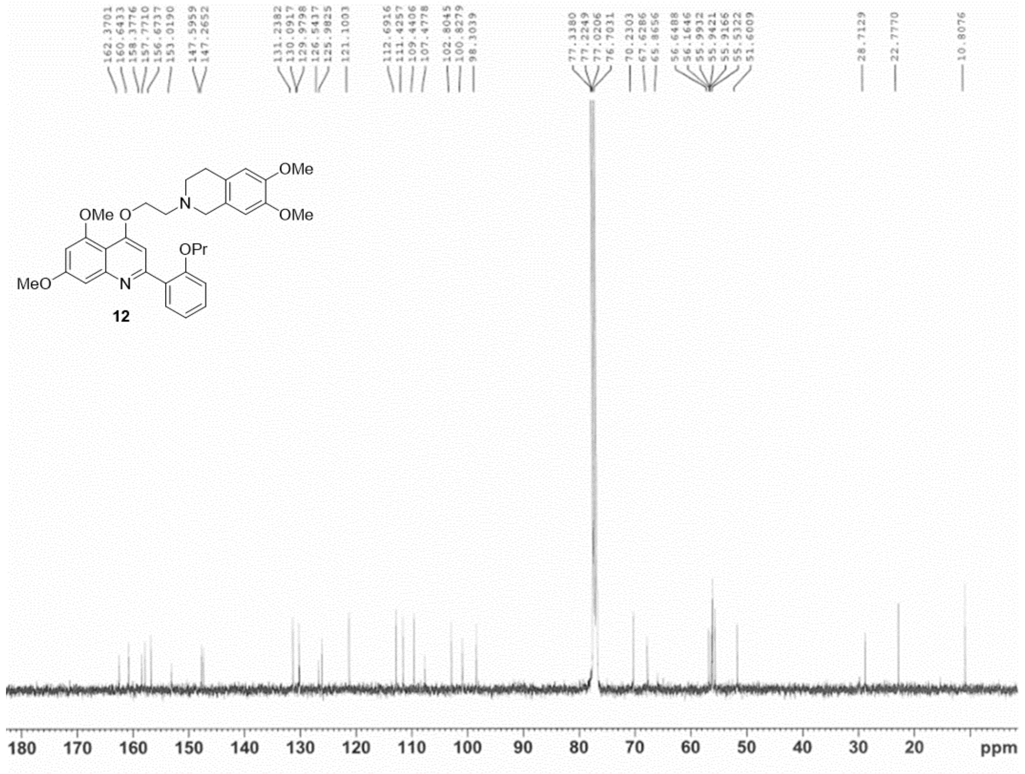

Figure S20. ^{13}C NMR (101 MHz, $CDCl_3$) spectrum of compound **12**.

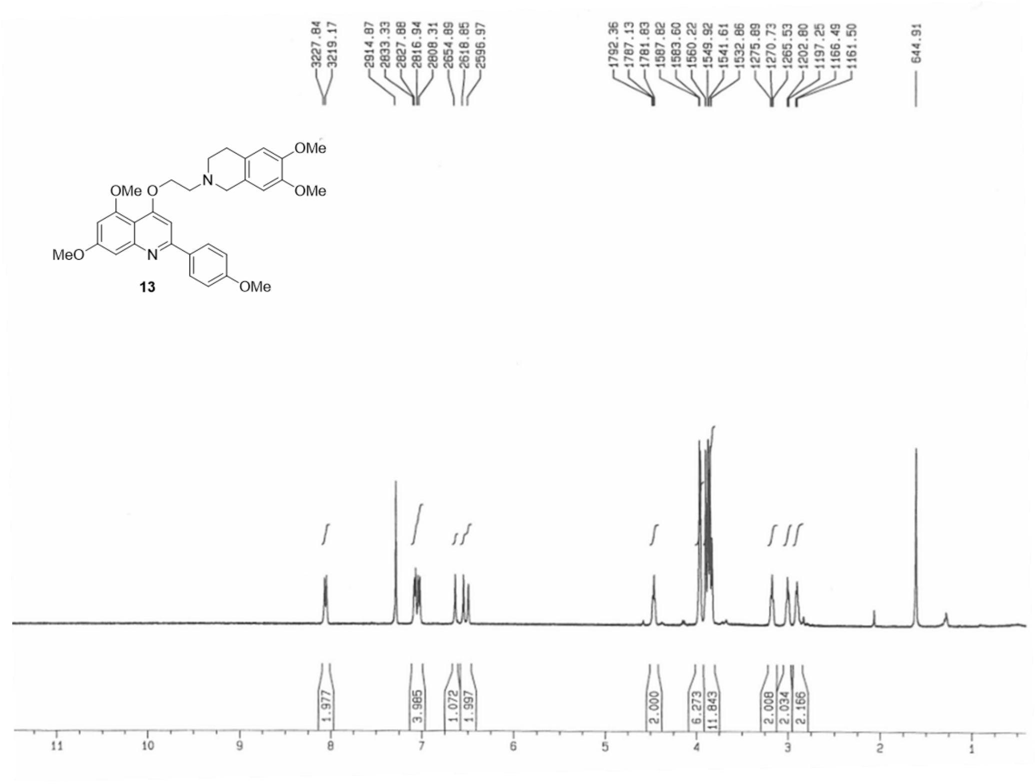

Figure S21. ^{1}H NMR (400 MHz, $CDCl_3$) spectrum of compound **13**.

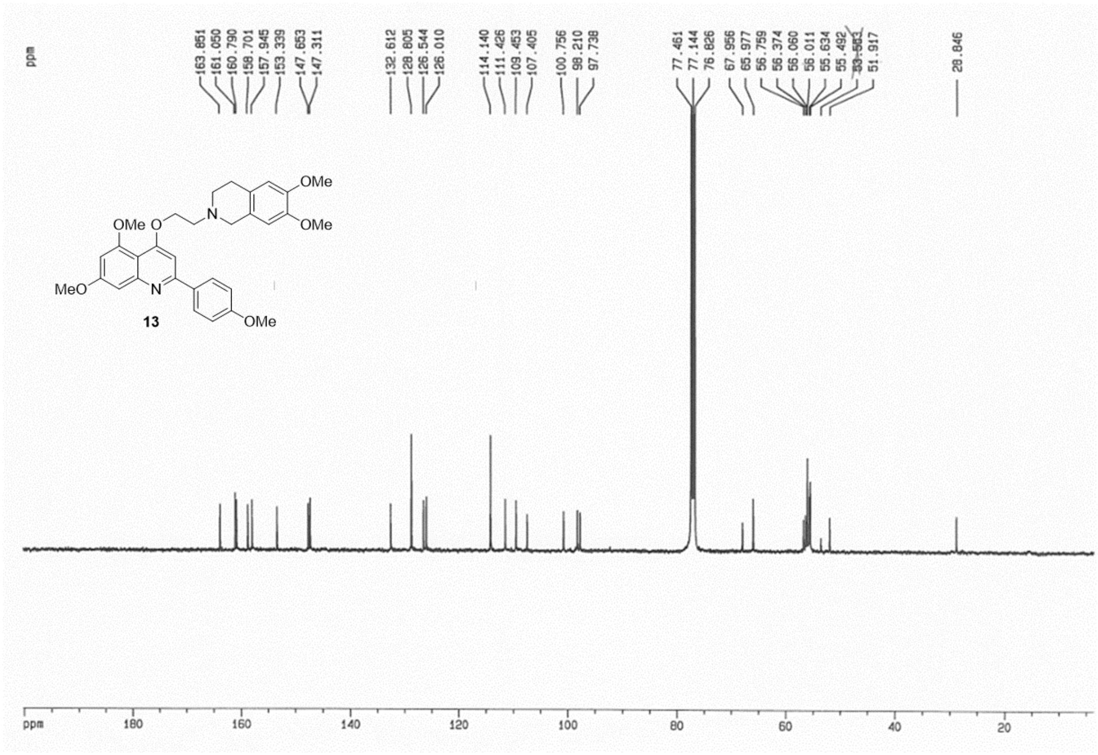

Figure S22. ^{13}C NMR (101 MHz, $CDCl_3$) spectrum of compound **13**.

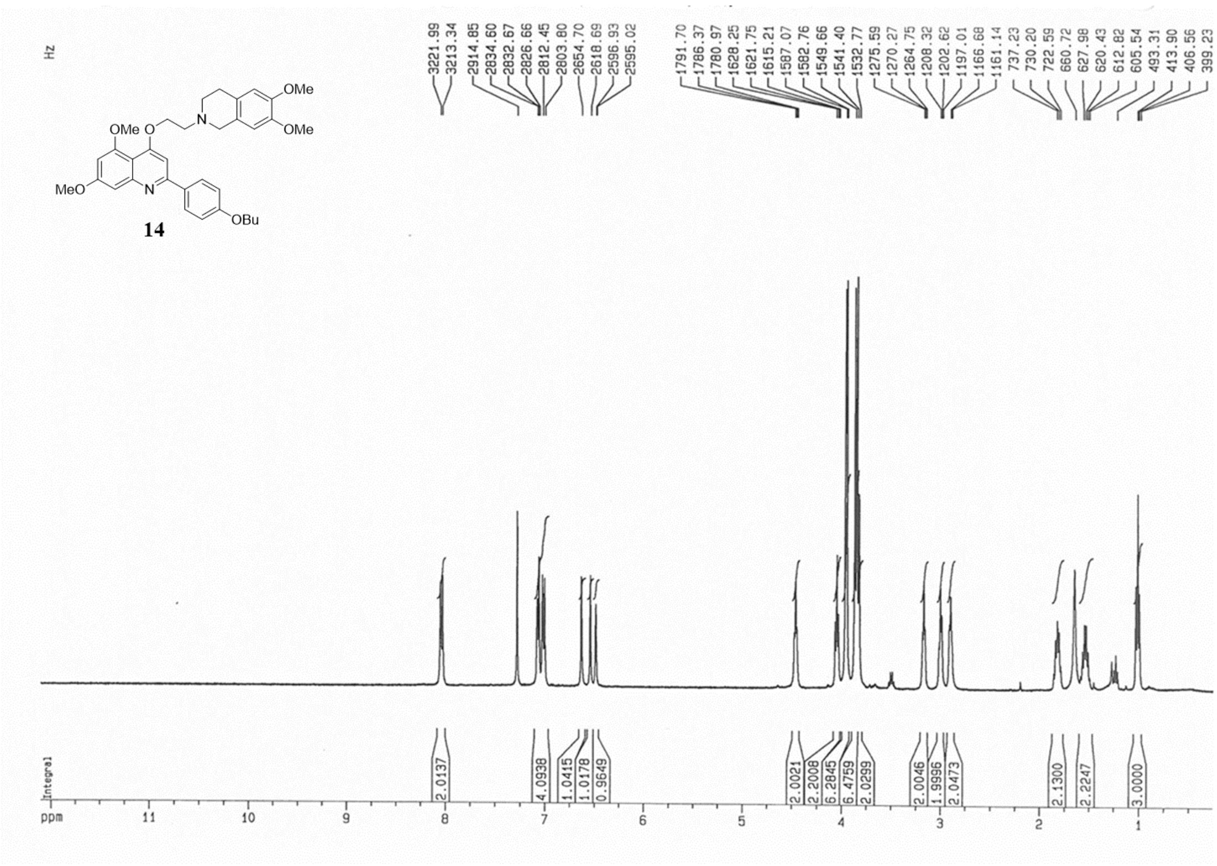

Figure S23. ^{1}H NMR (400 MHz, $CDCl_3$) spectrum of compound **14**.

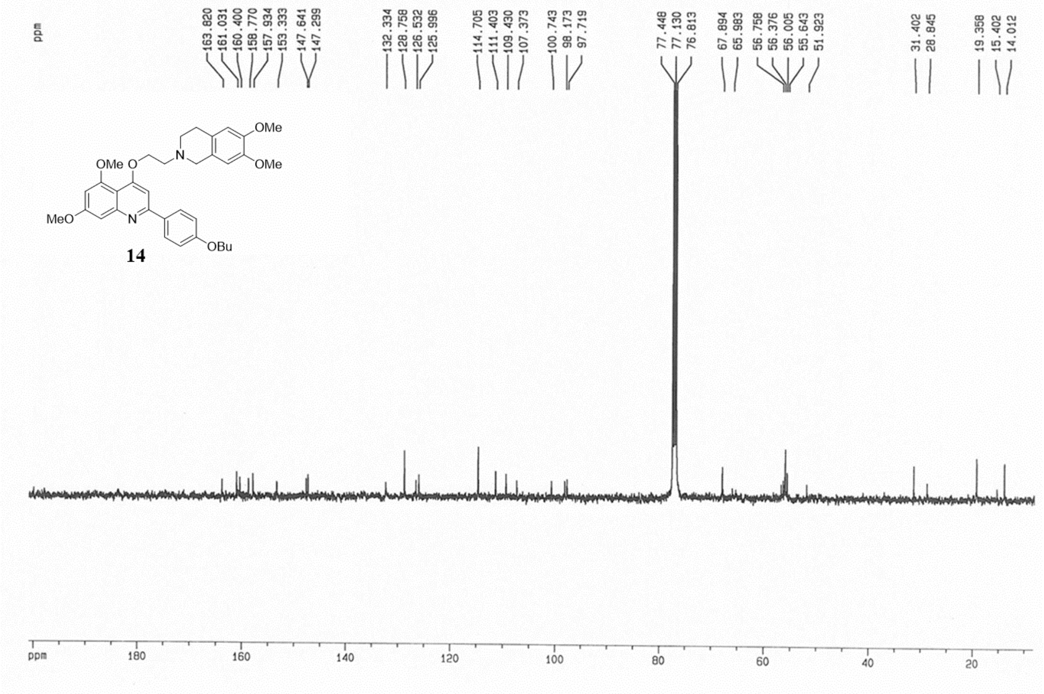

Figure S24. ^{13}C NMR (101 MHz, $CDCl_3$) spectrum of compound **14**.

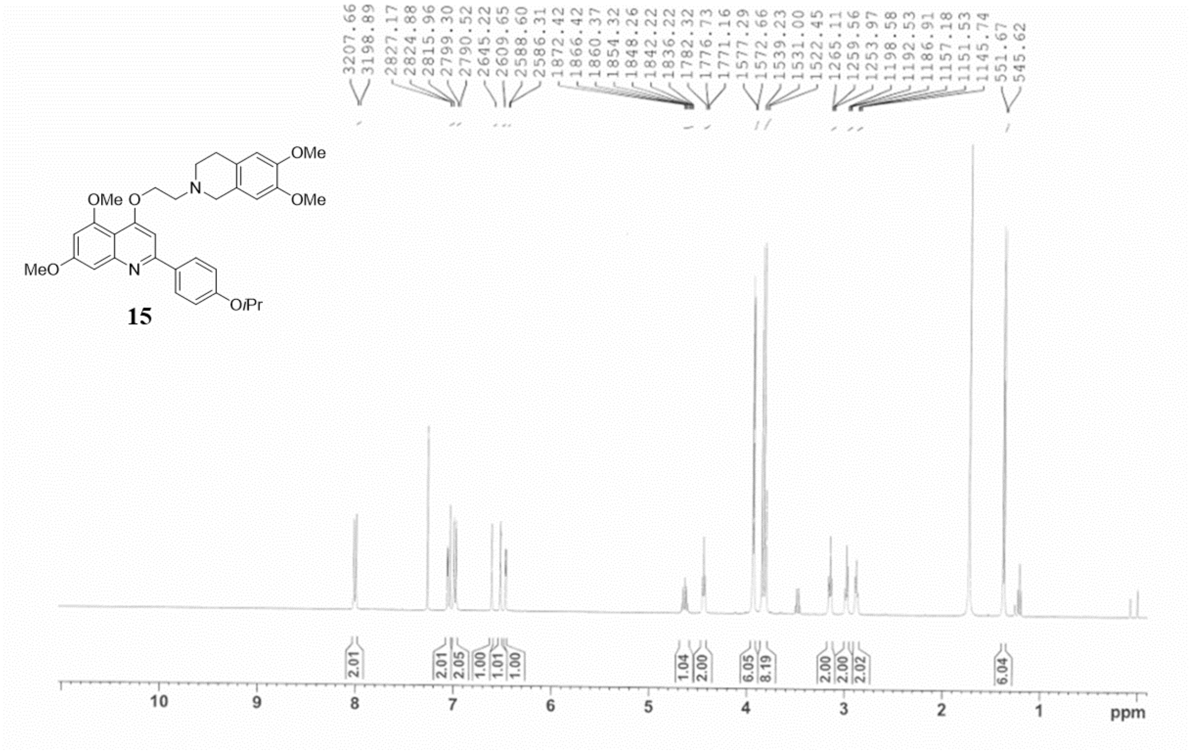

Figure S25. ^{1}H NMR (400 MHz, $CDCl_3$) spectrum of compound **15**.

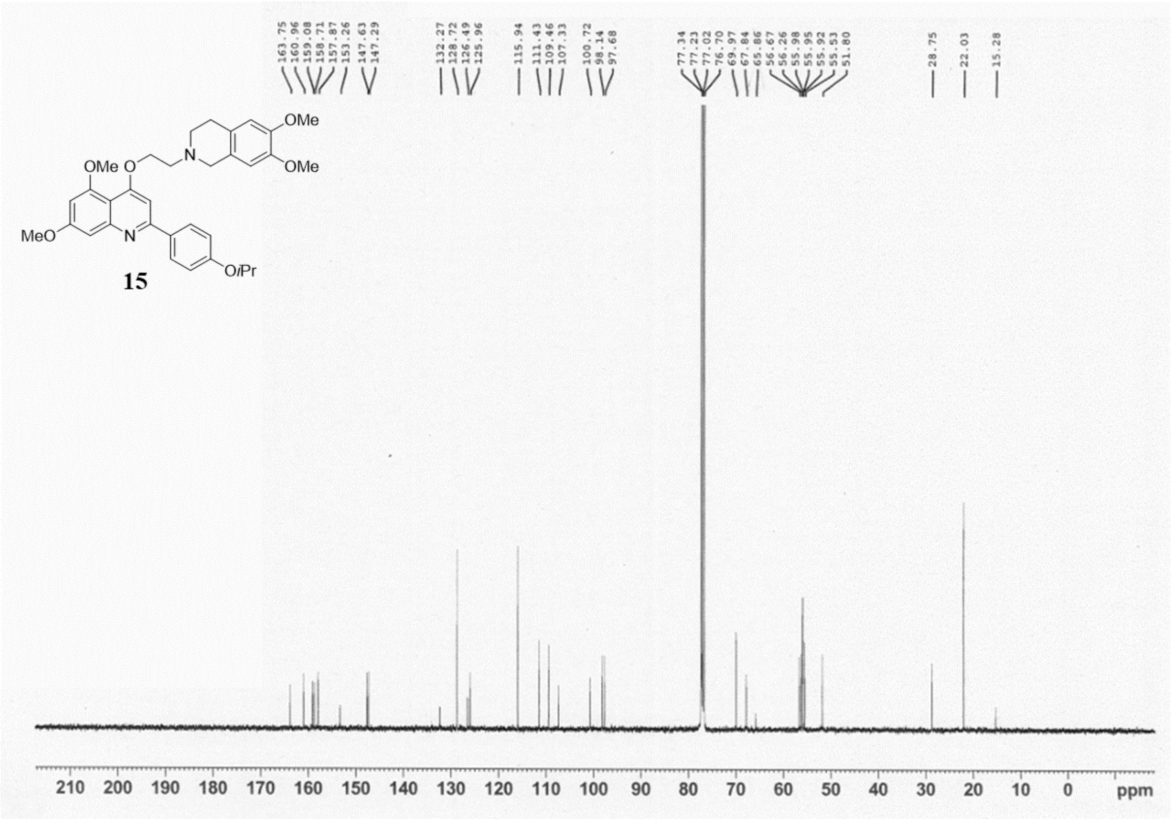

Figure S26. ^{13}C NMR (101 MHz, $CDCl_3$) spectrum of compound **15**.

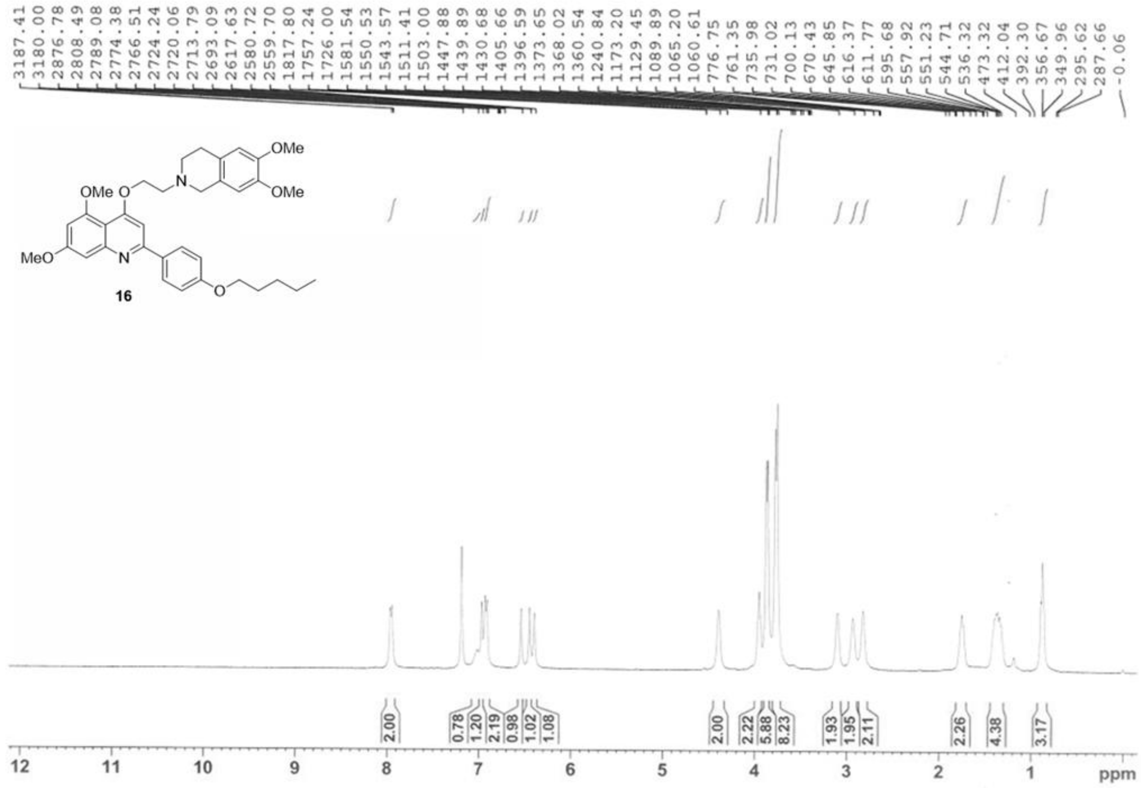

Figure S27. ^{1}H NMR (400 MHz, $CDCl_3$) spectrum of compound **16**.

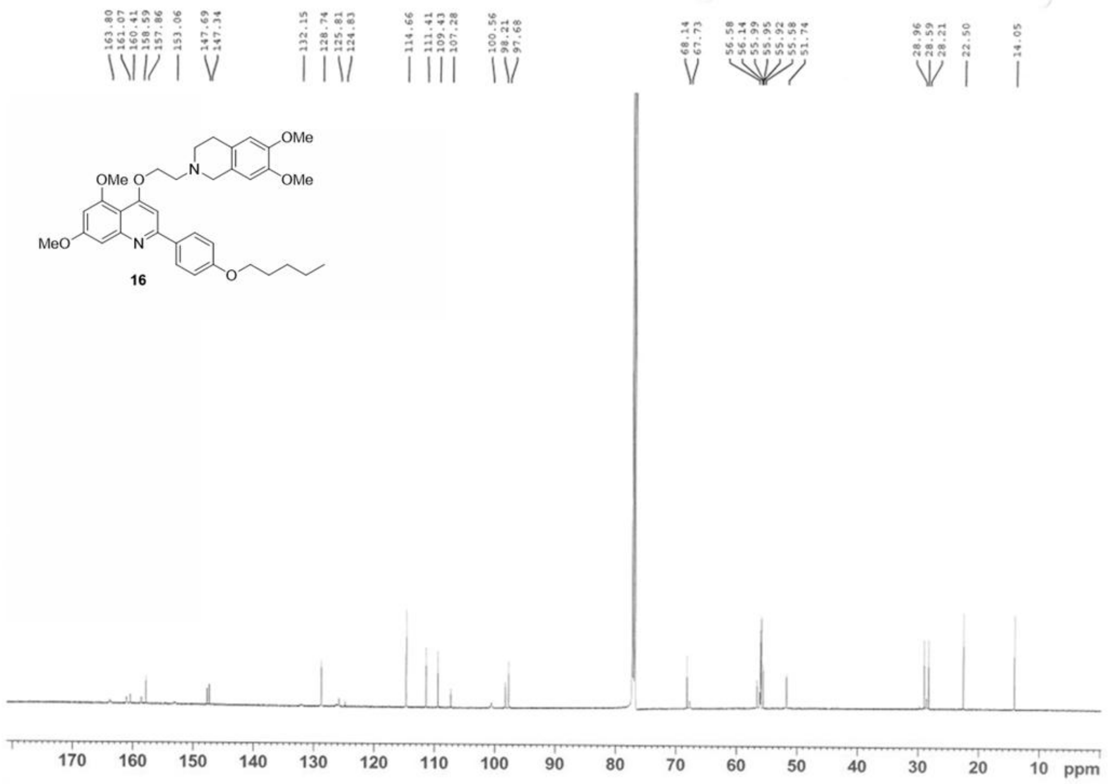

Figure S28. ^{13}C NMR (101 MHz, $CDCl_3$) spectrum of compound **16**.

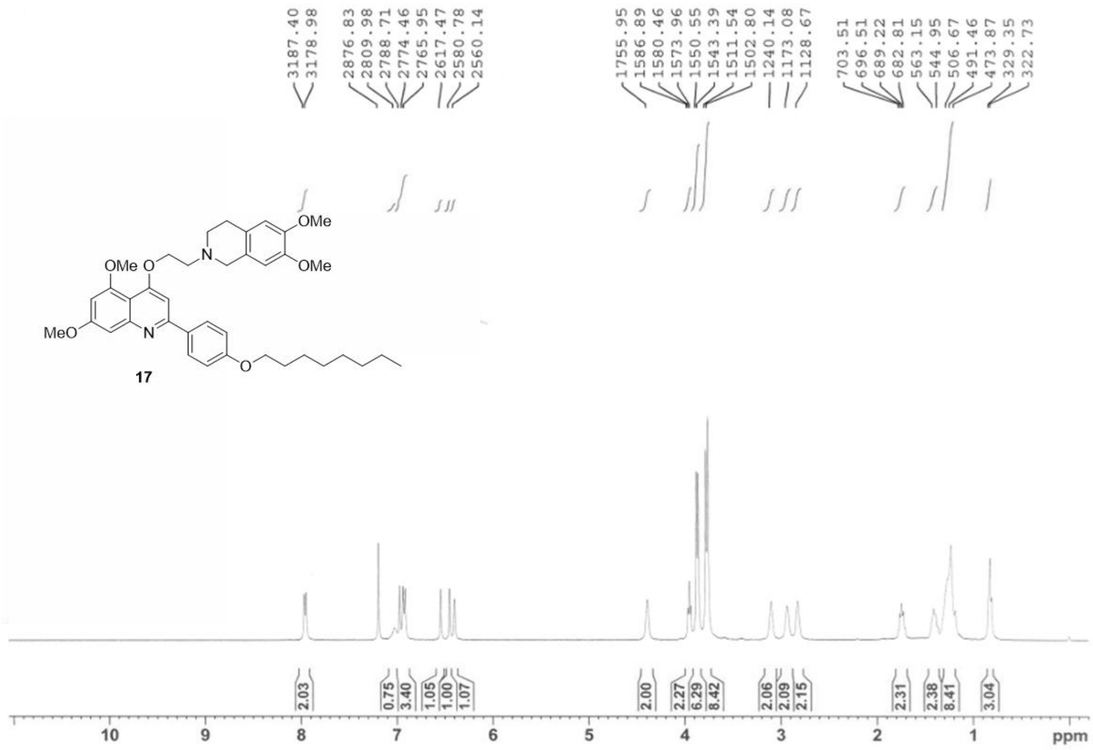

Figure S29. ^{1}H NMR (400 MHz, $CDCl_3$) spectrum of compound **17**.

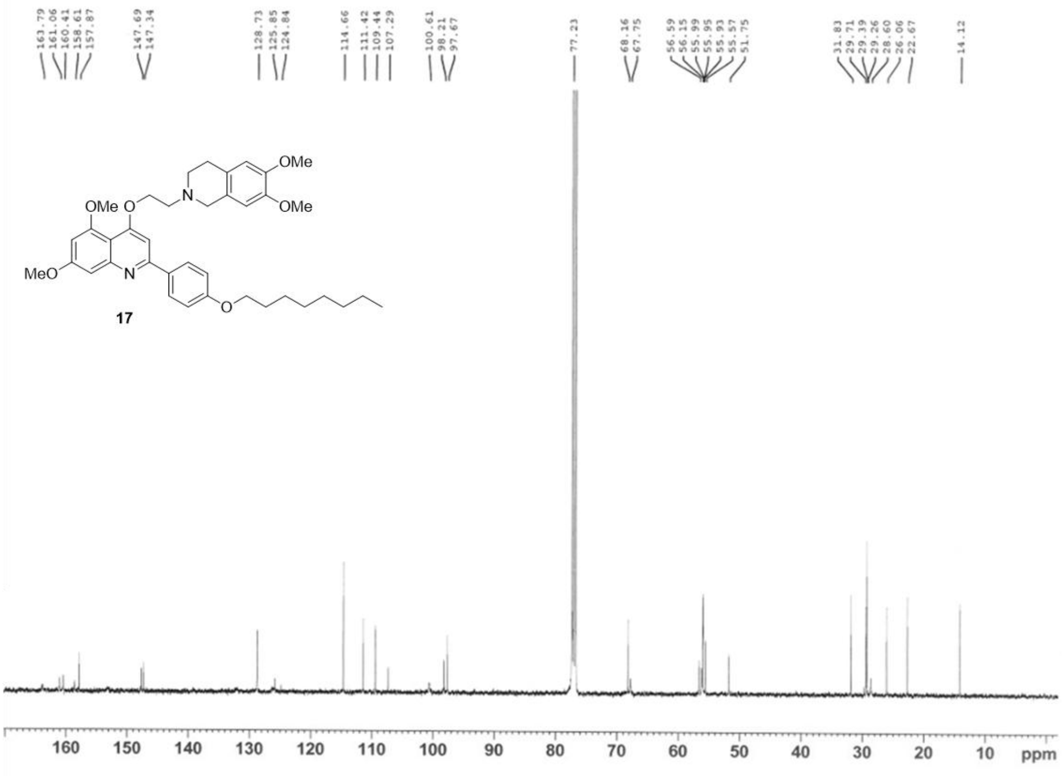

Figure S30. ^{13}C NMR (101 MHz, $CDCl_3$) spectrum of compound **17**.

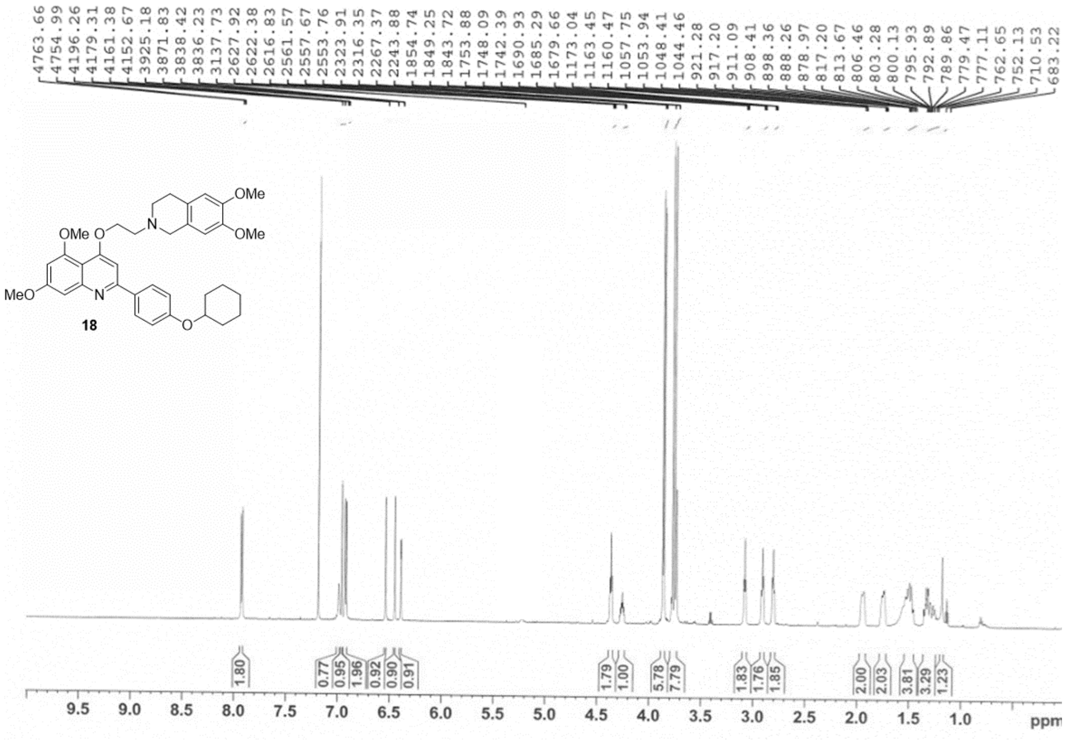

Figure S31. ^{1}H NMR (400 MHz, $CDCl_3$) spectrum of compound **18**.

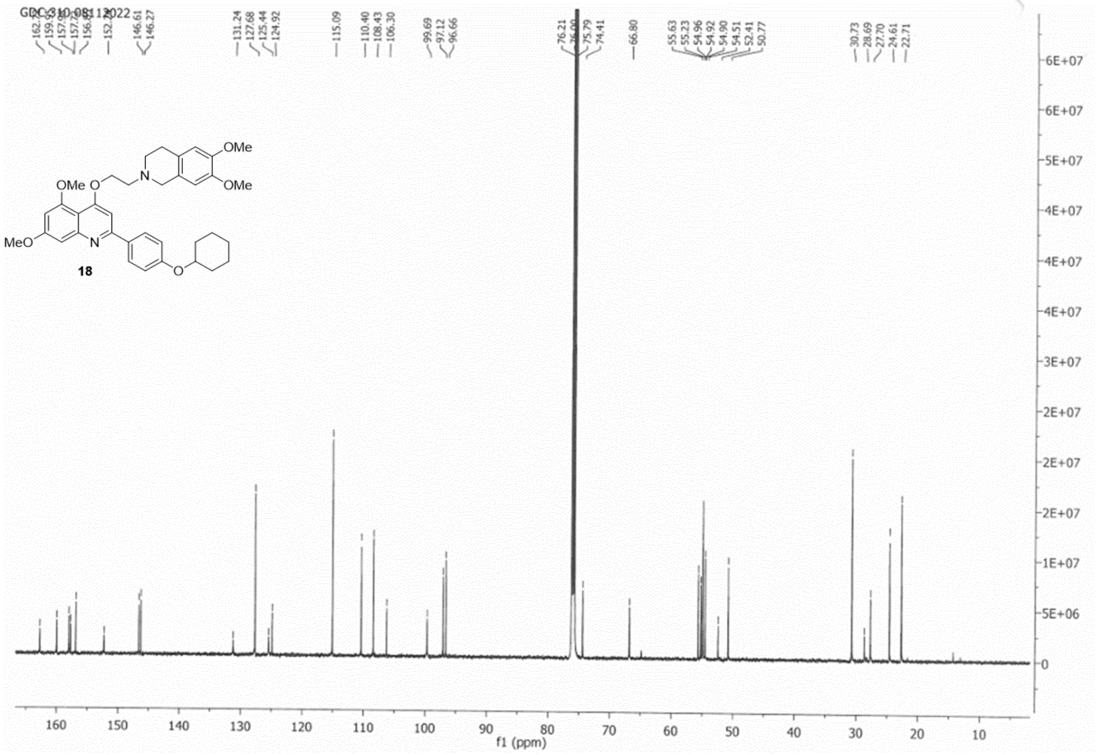

Figure S32. ^{13}C NMR (101 MHz, $CDCl_3$) spectrum of compound **18**.

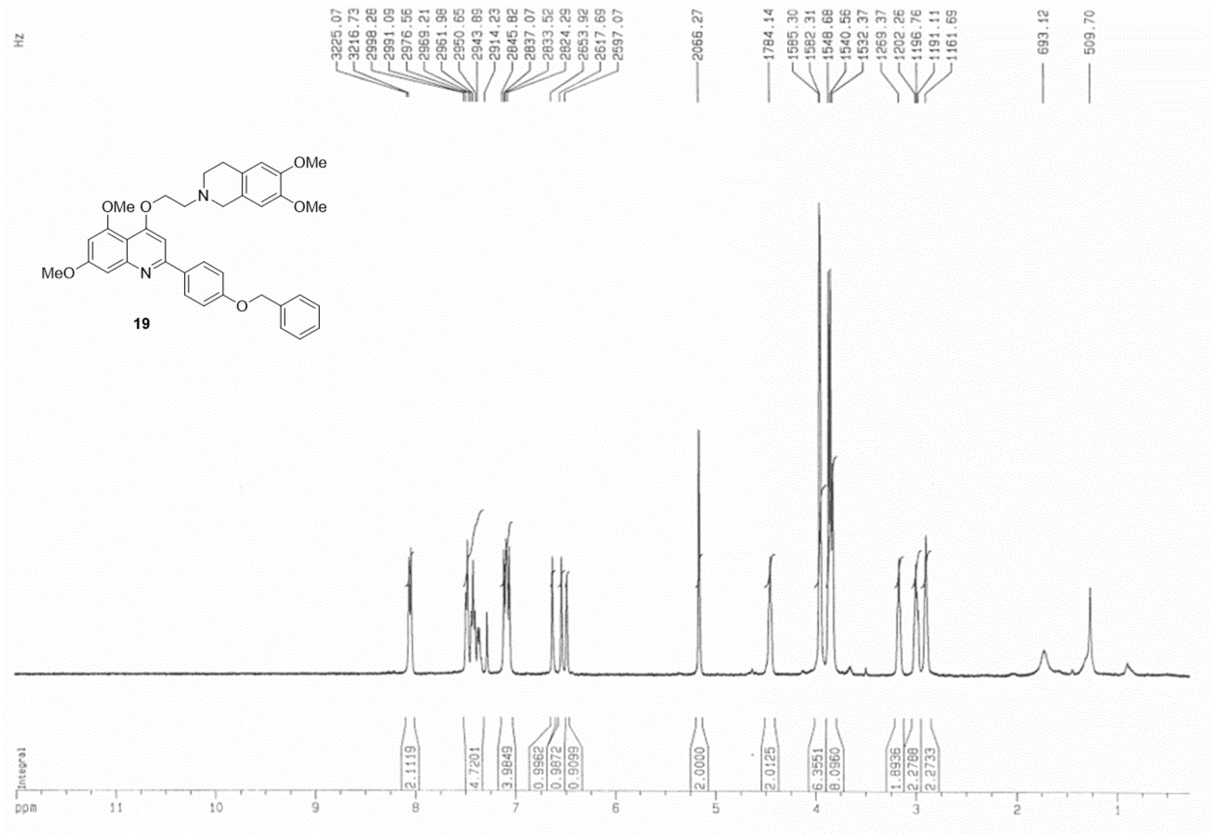

Figure S33. ^{1}H NMR (400 MHz, $CDCl_3$) spectrum of compound **19**.

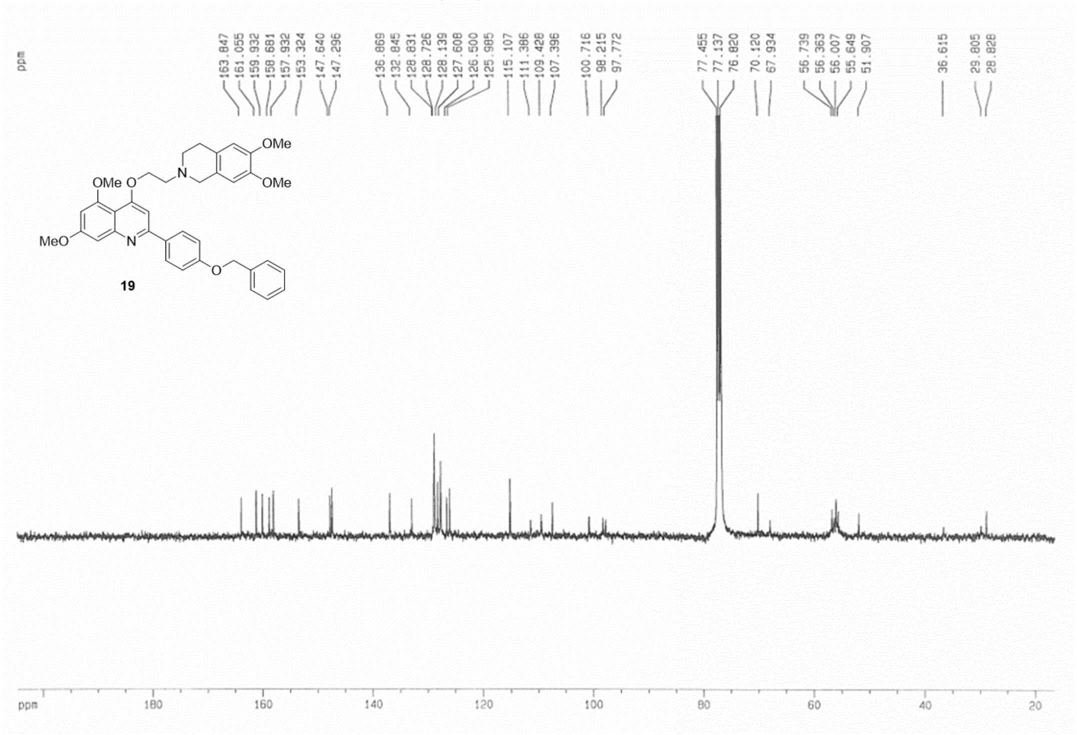

Figure S34. ^{13}C NMR (101 MHz, $CDCl_3$) spectrum of compound **19**.

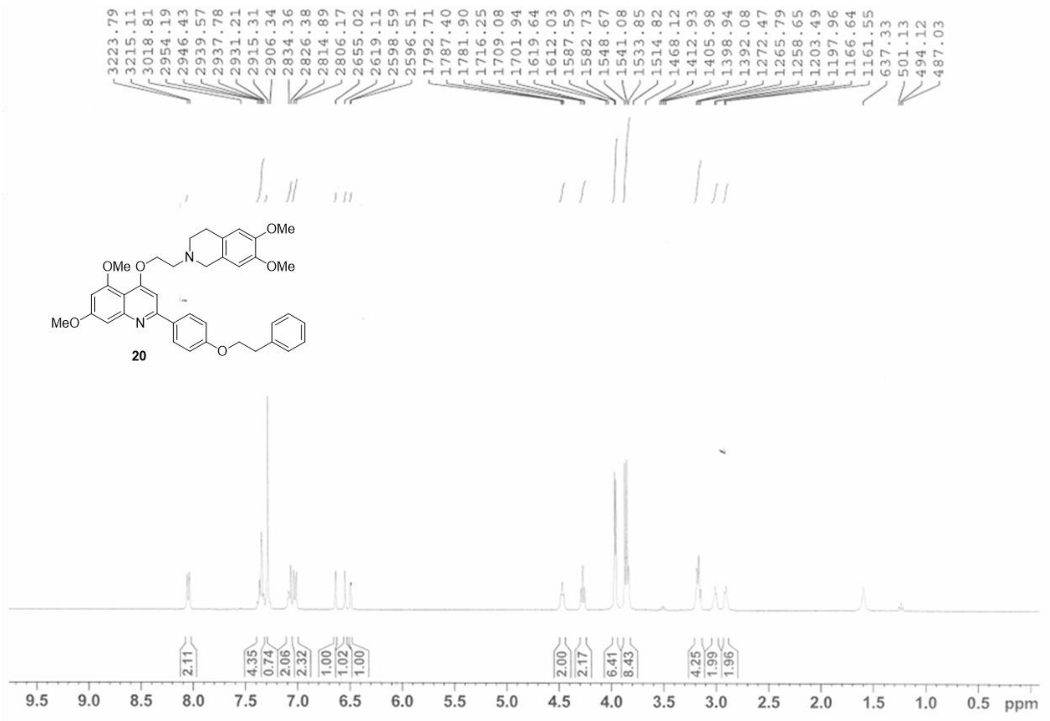

Figure S35. ^{1}H NMR (400 MHz, $CDCl_3$) spectrum of compound **20**.

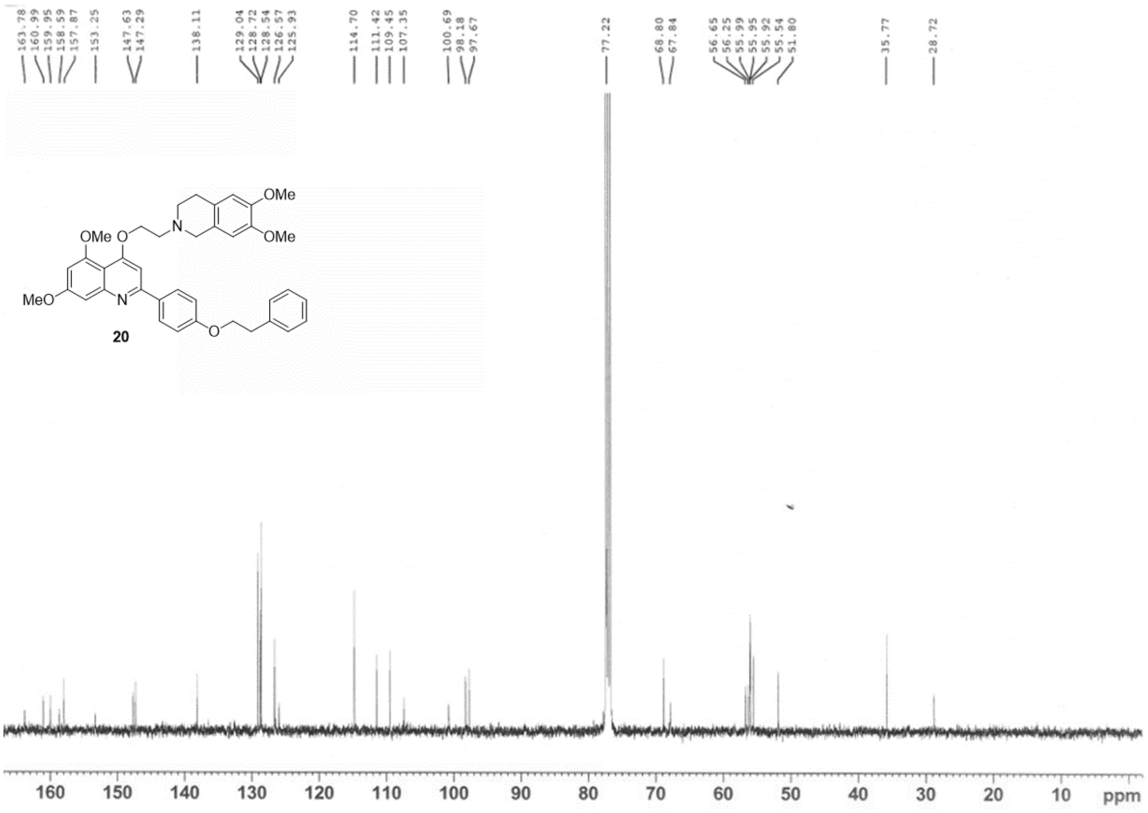

Figure S36. ^{13}C NMR (101 MHz, $CDCl_3$) spectrum of compound **20**.

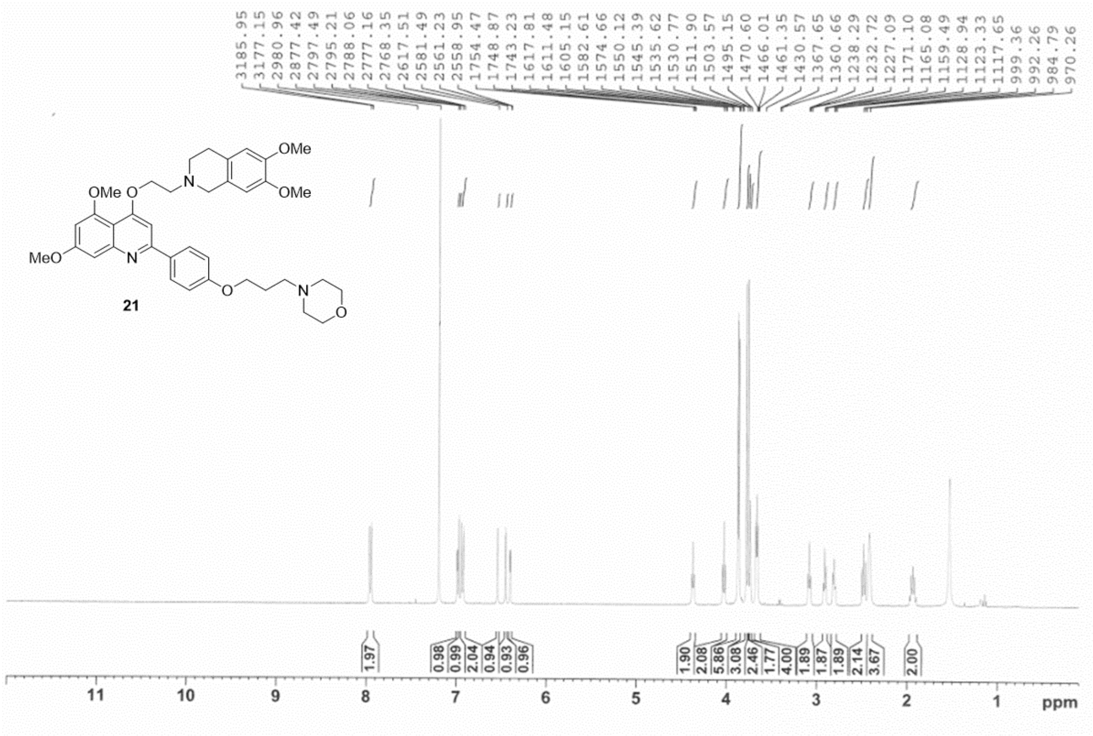

Figure S37. ^{1}H NMR (400 MHz, $CDCl_3$) spectrum of compound **21**.

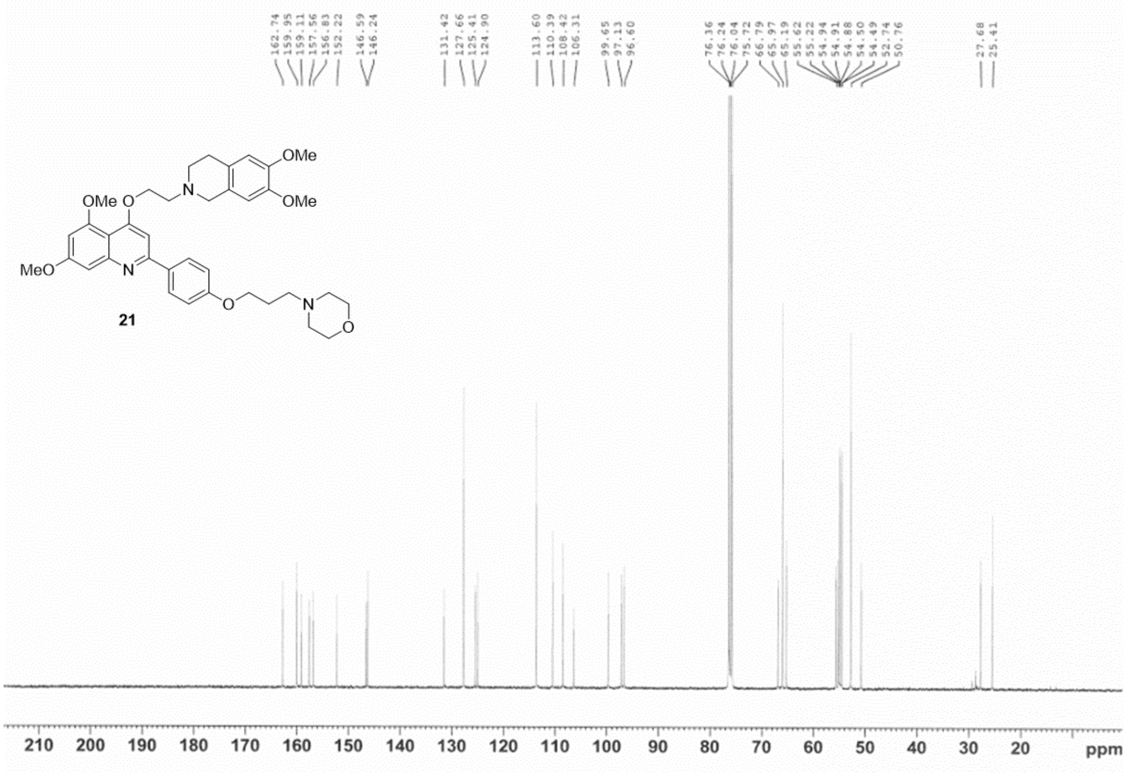

Figure S38. ^{13}C NMR (101 MHz, $CDCl_3$) spectrum of compound **21**.

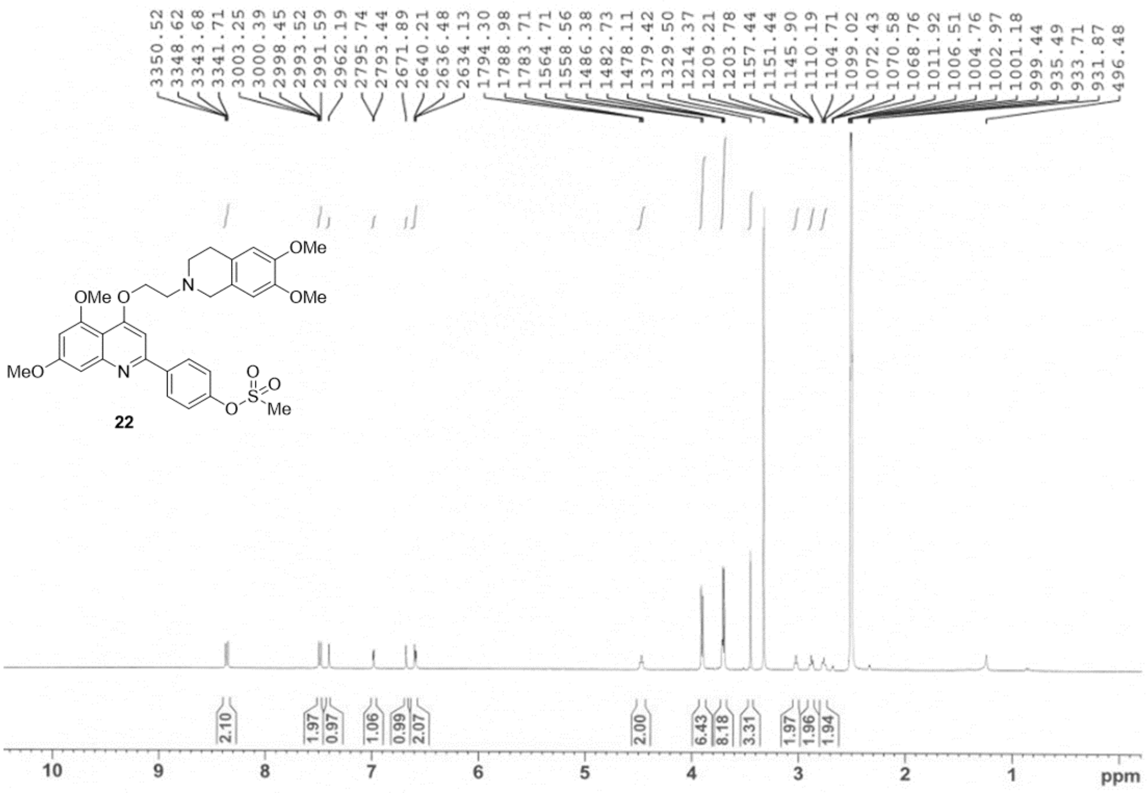

Figure S39. ^{1}H NMR (400 MHz, DMSO-d_6) spectrum of compound **22**.

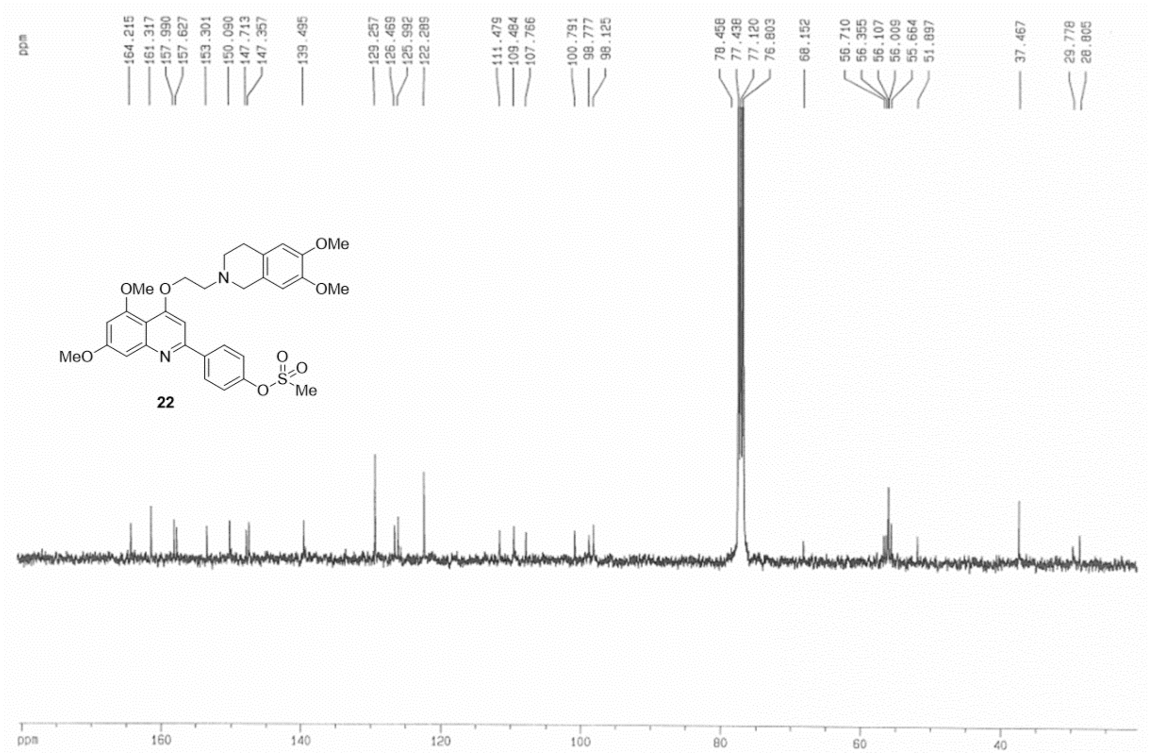

Figure S40. ^{13}C NMR (101 MHz, DMSO-d_6) spectrum of compound **22**.

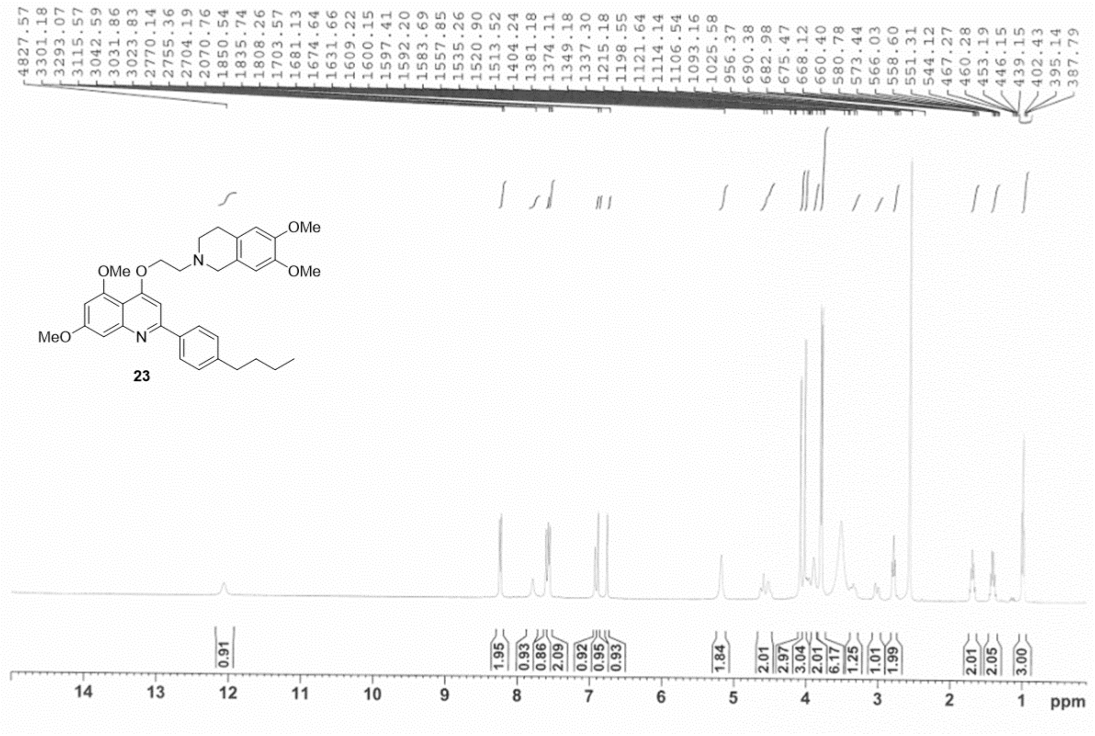

Figure S41. ^{1}H NMR (400 MHz, DMSO-d_6) spectrum of compound **23**.

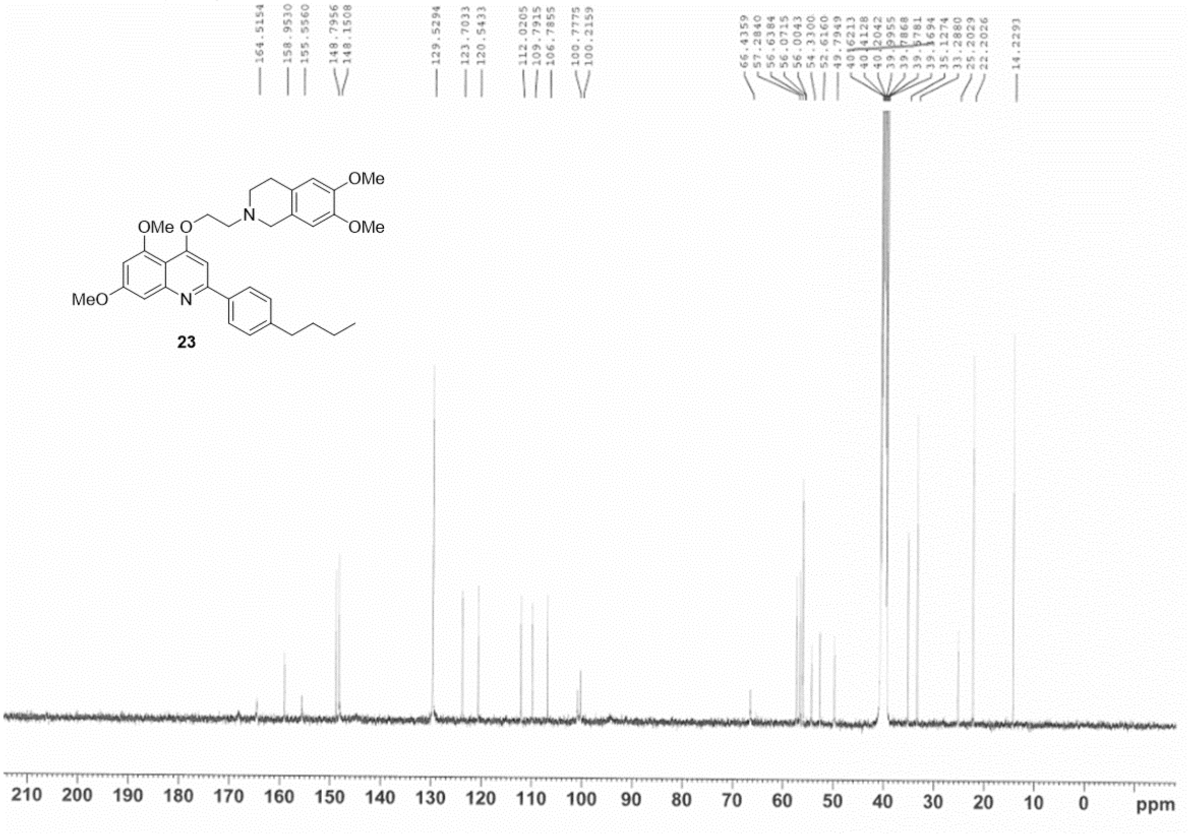

Figure S42. ^{13}C NMR (101 MHz, DMSO-d_6) spectrum of compound **23**.

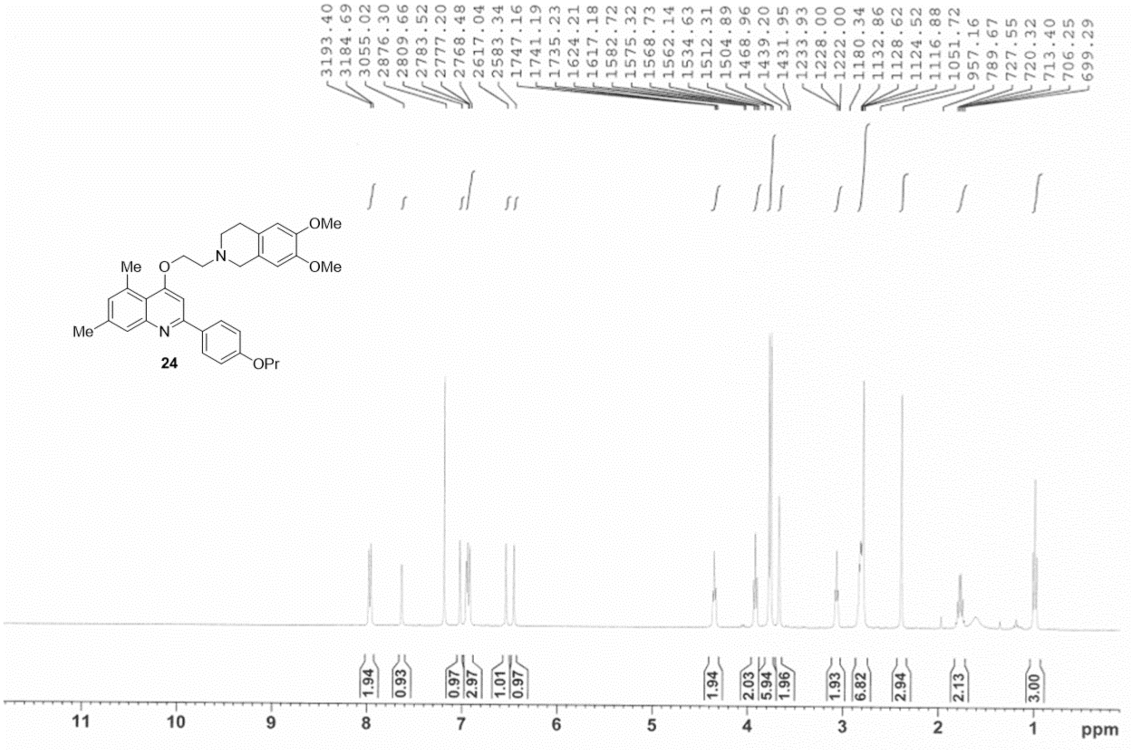

Figure S43. ^{1}H NMR (400 MHz, $CDCl_3$) spectrum of compound **24**.

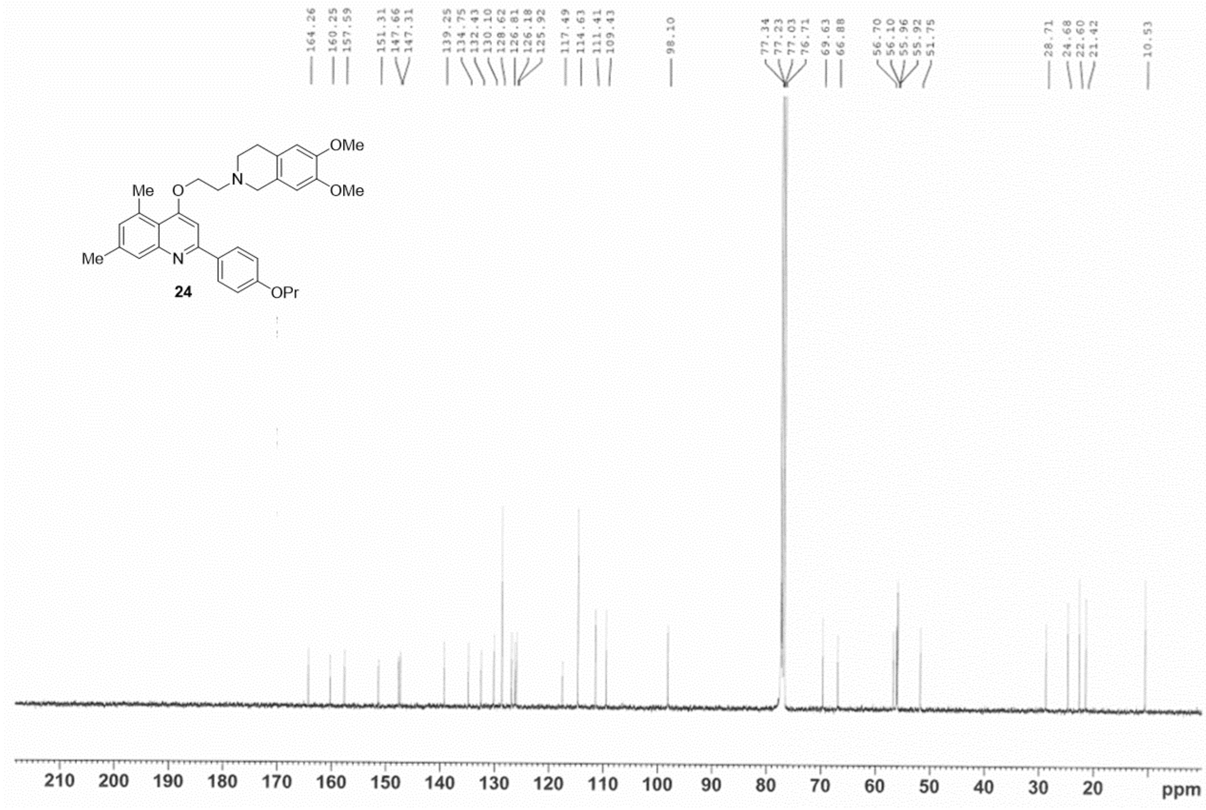

Figure S44. ^{13}C NMR (101 MHz, $CDCl_3$) spectrum of compound **24**.

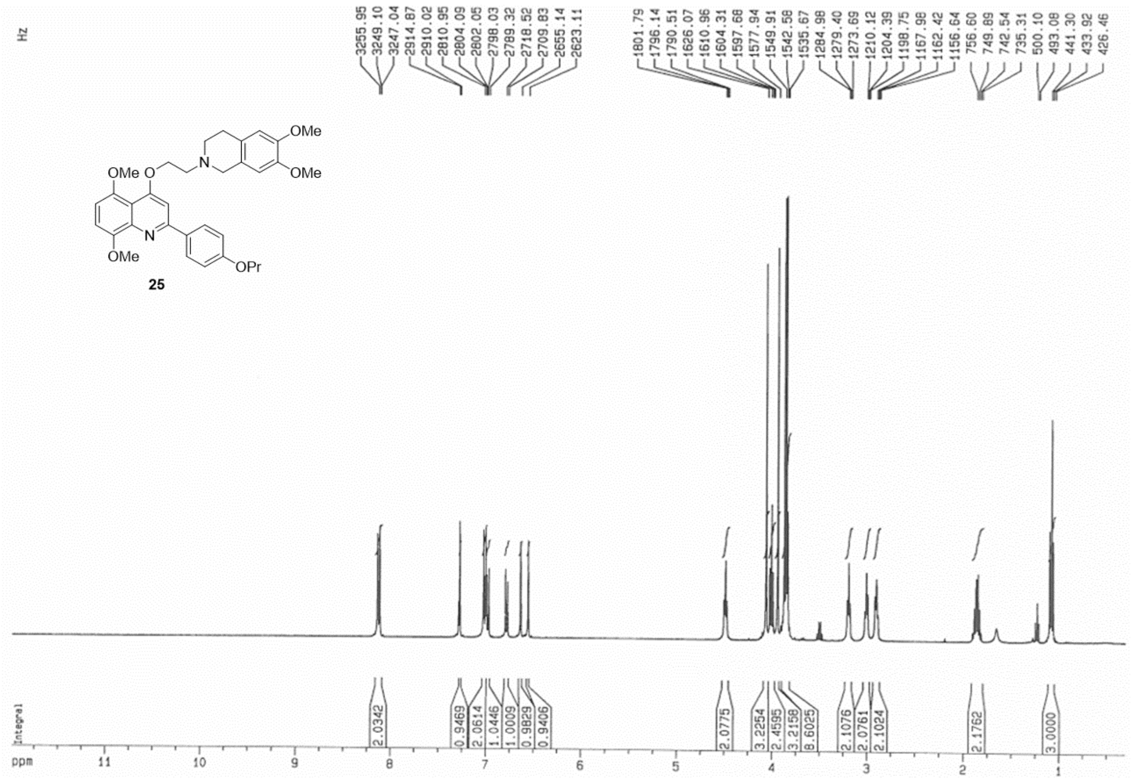

Figure S45. [1] H NMR (101 MHz, $CDCl_3$) spectrum of compound **25**.

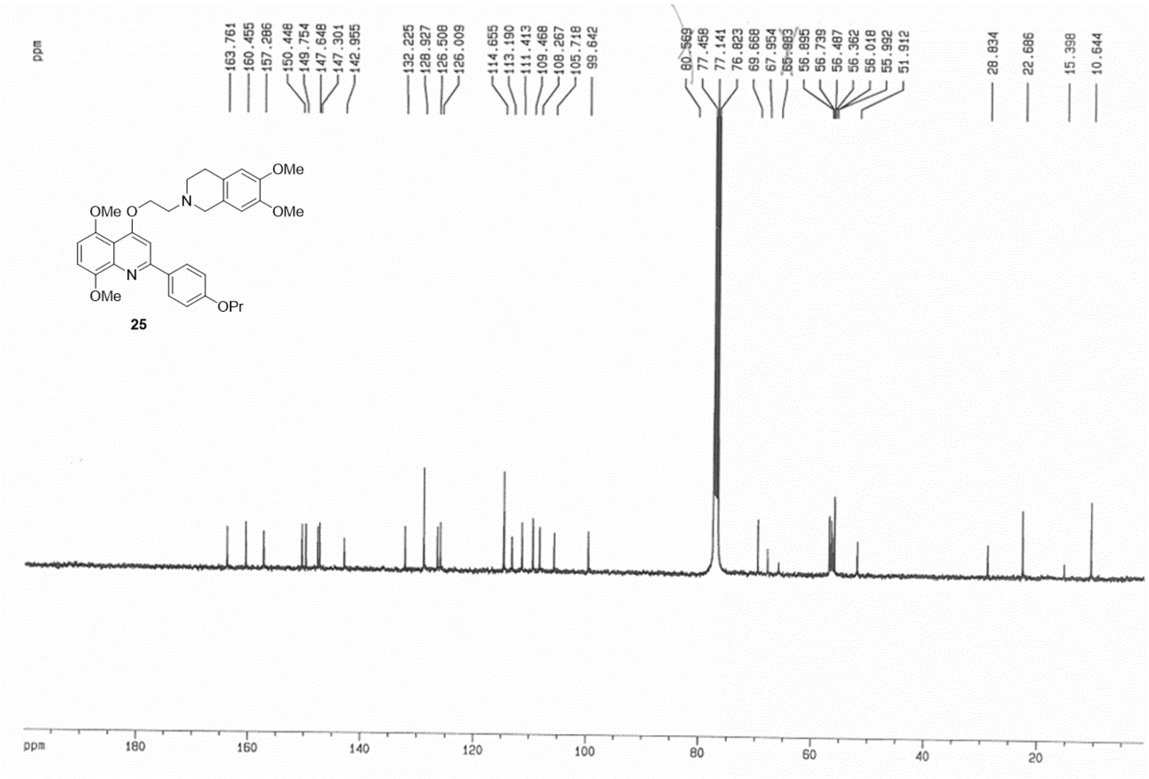

Figure S46. ^{13}C NMR (101 MHz, $CDCl_3$) spectrum of compound **25**.

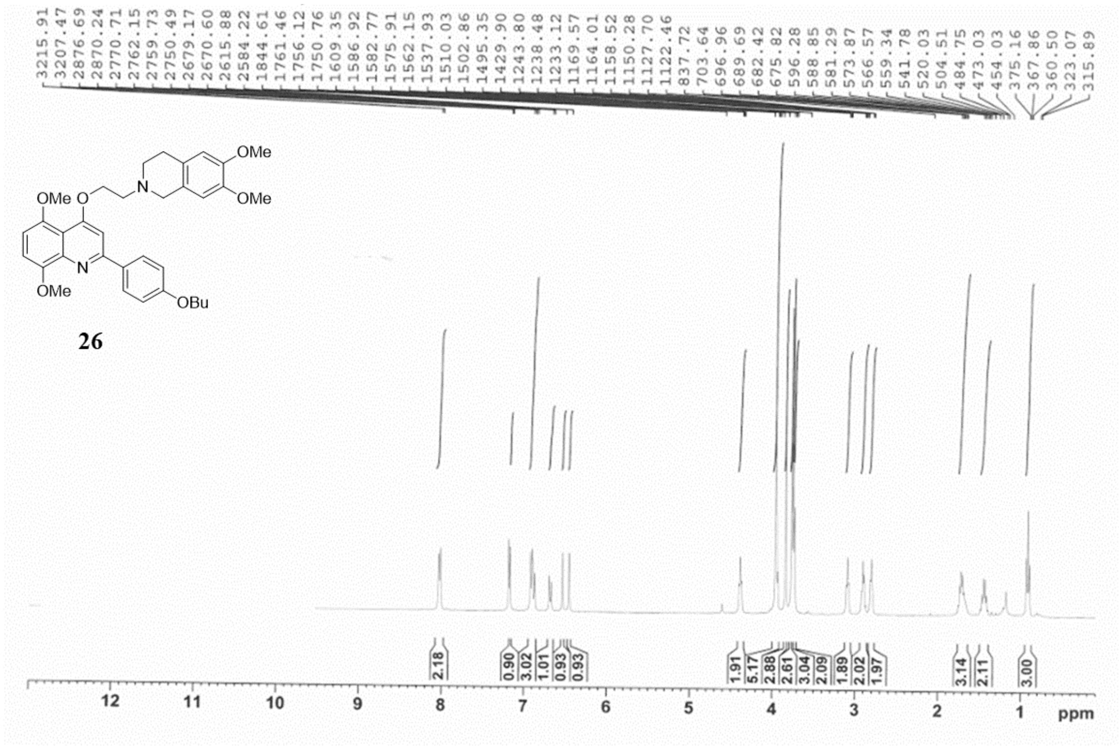

Figure S47. ^{1}H NMR (400 MHz, $CDCl_3$) spectrum of compound **26**.

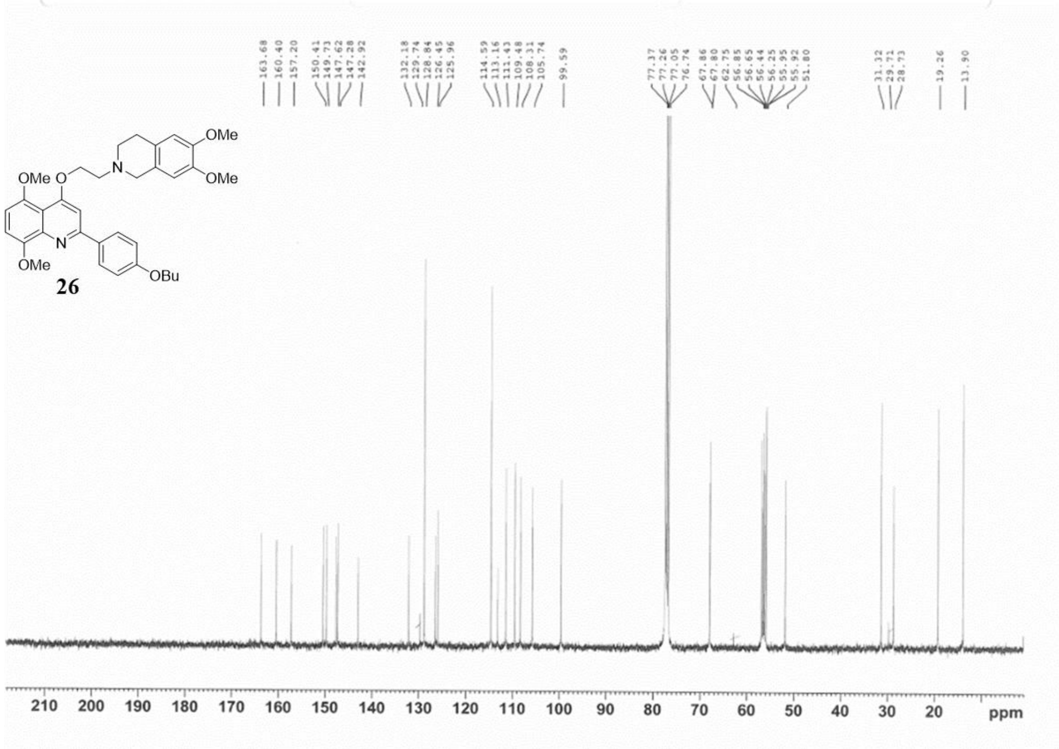

Figure S48. ^{13}C NMR (101 MHz, $CDCl_3$) spectrum of compound **26**.

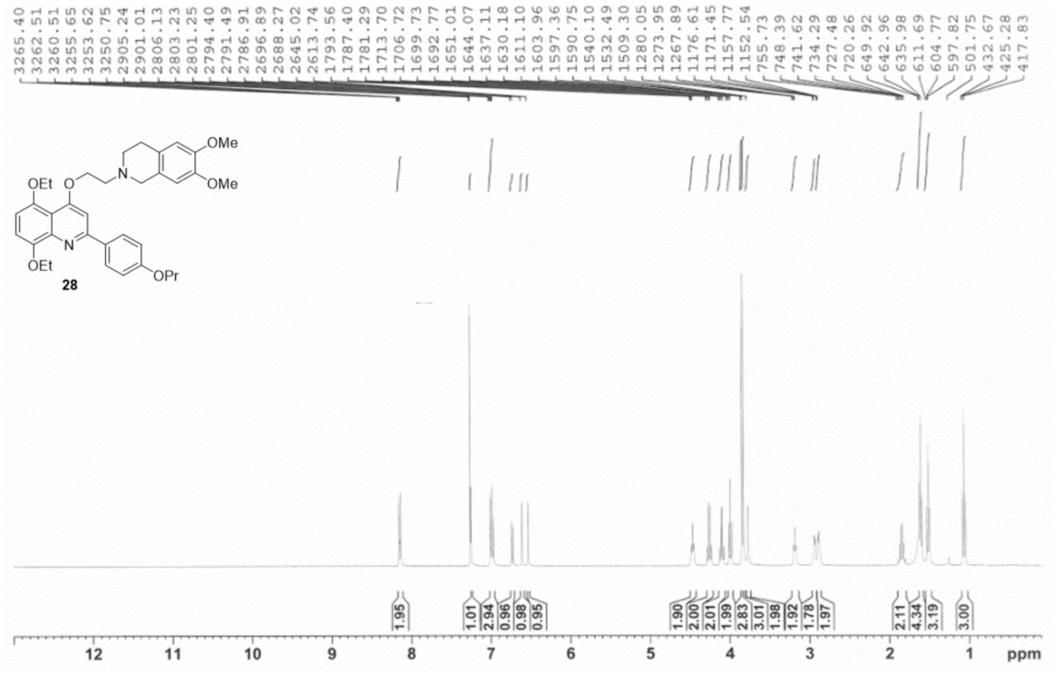

Figure S49. [1] H NMR (400 MHz, $CDCl_3$) spectrum of compound **28**.

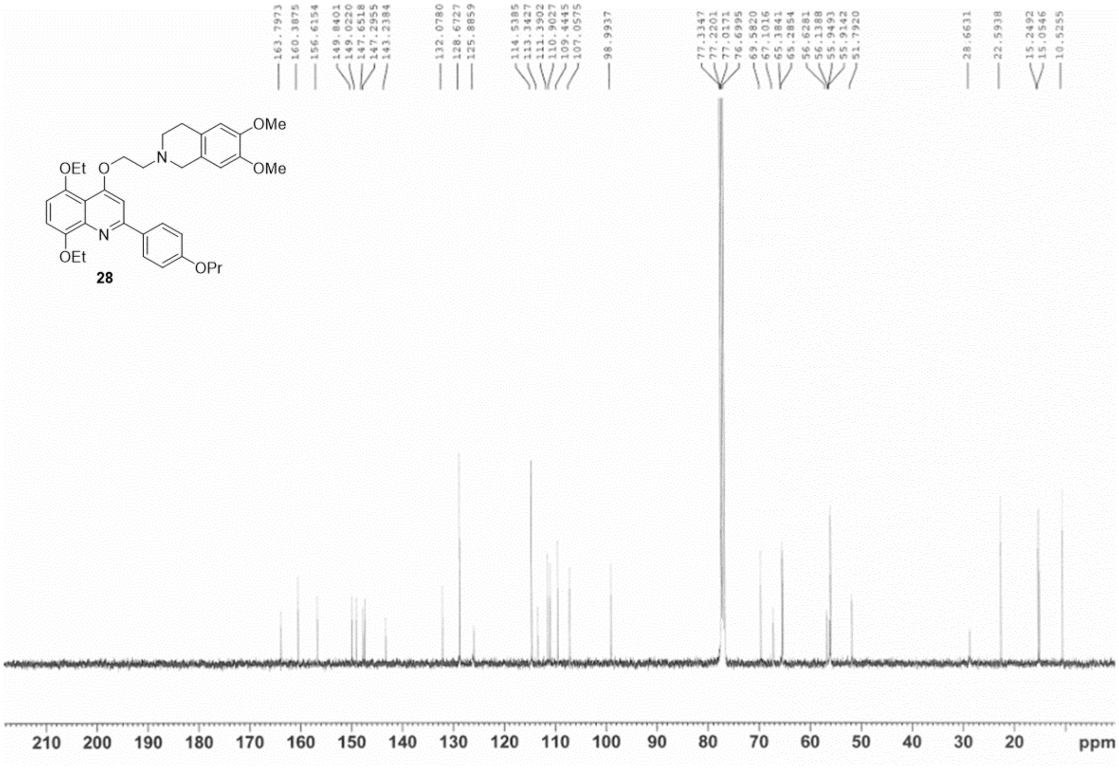

Figure S50. ^{13}C NMR (101 MHz, $CDCl_3$) spectrum of compound **28**.

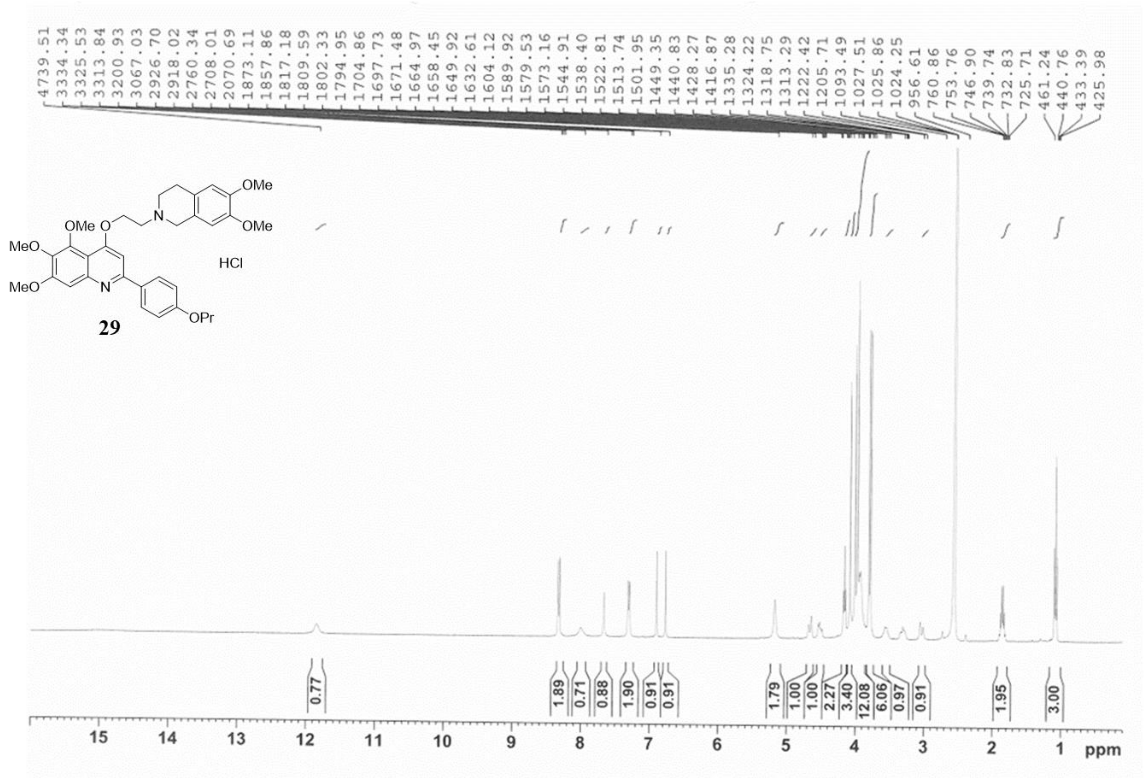

Figure S51. ^{1}H NMR (400 MHz, DMSO-d_6) spectrum of compound **29**.

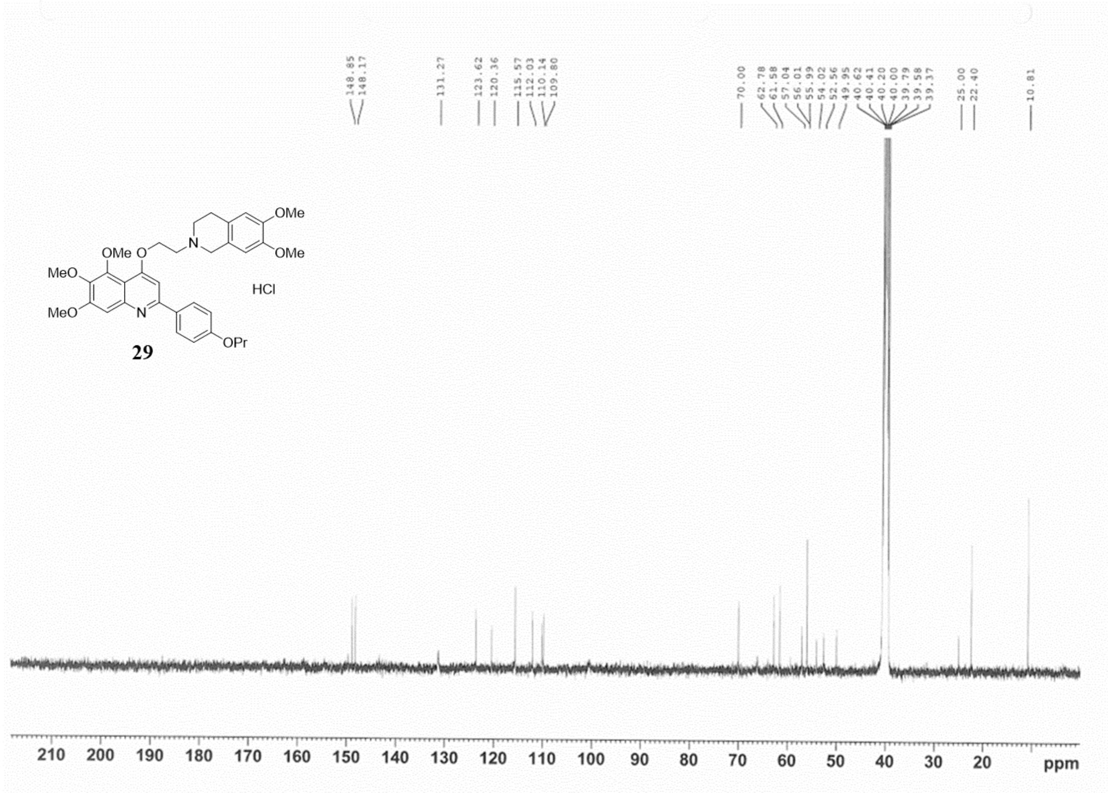

Figure S52. ^{13}C NMR (101 MHz, DMSO-d_6) spectrum of compound **29**.

1.2. HPLC analysis of exemplary target compounds

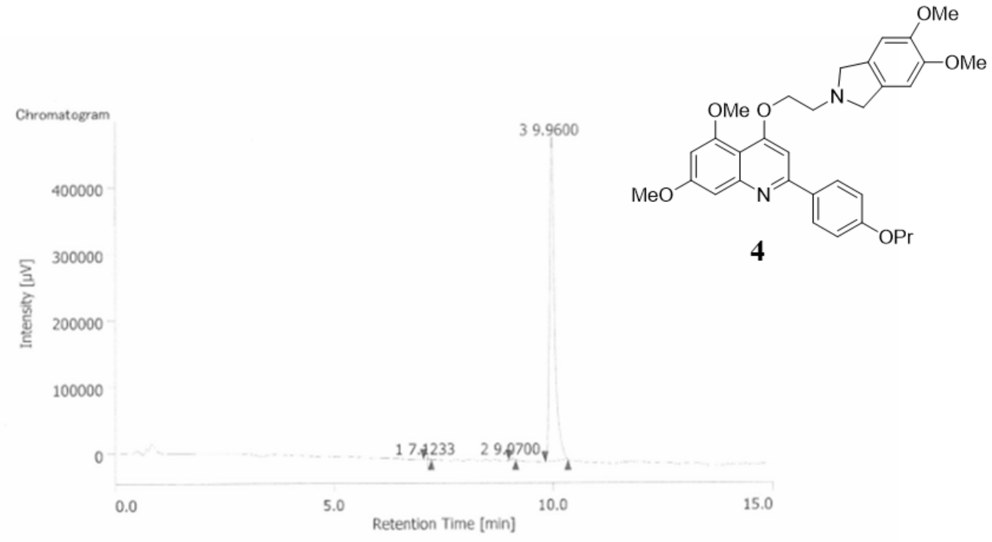

Peak Information

#	Peak Name	CH	tR [min]	Area [µV·sec]	Height [µV]	Area%	Height%	Quantity
1	Peak-001	5	7.123	14833	2837	0.373	0.575	N/A
2	Peak-002	5	9.070	18220	3536	0.458	0.717	N/A
3	Peak-003	5	9.960	3946071	487040	99.169	98.708	N/A

Figure S53. HPLC of compound **4**.

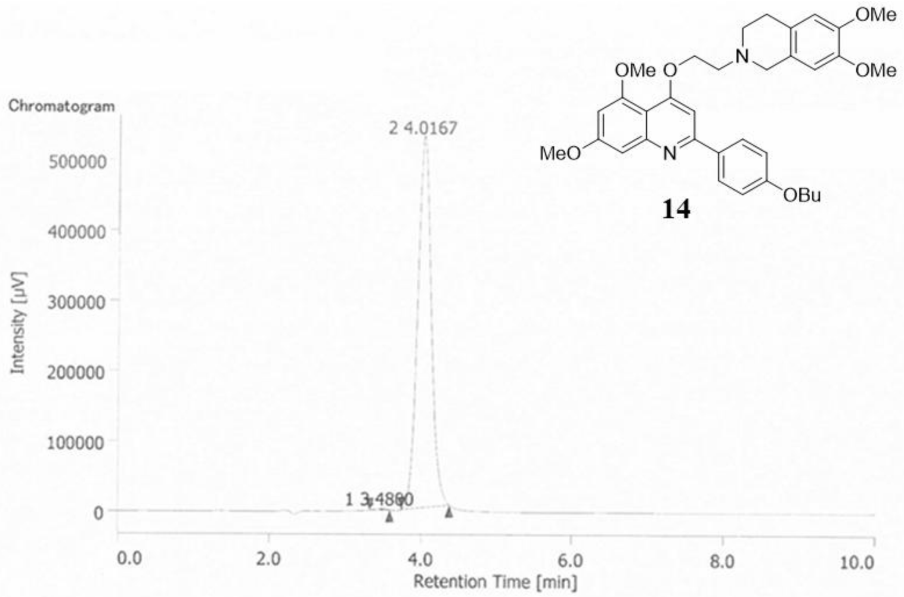

Peak Information

#	Peak Name	CH	tR [min]	Area [µV·sec]	Height [µV]	Area%	Height%	Quantity
1	Peak-001	5	3.480	15624	1550	0.236	0.292	N/A
2	Peak-002	5	4.017	6612508	529252	99.764	99.708	N/A

Figure S54. HPLC of compound **14**.

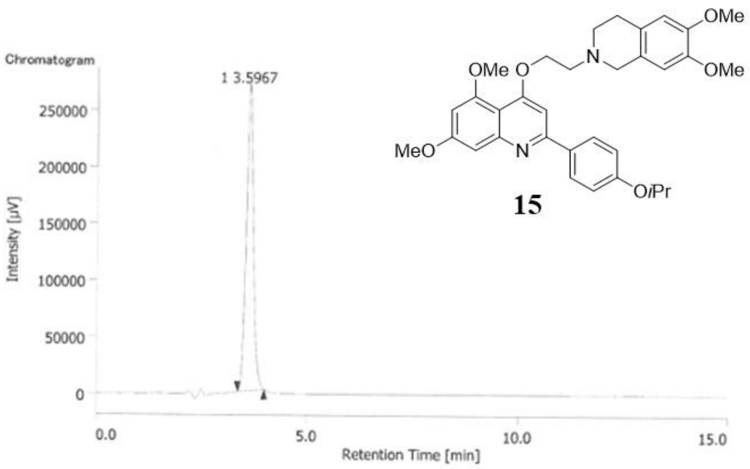

Peak Information

#	Peak Name	CH	tR [min]	Area [μV·sec]	Height [μV]	Area%	Height%	Quantity
1	Peak-001	5	3.597	3194532	270138	100.000	100.000	N/A

Figure S55. HPLC of compound **15**.

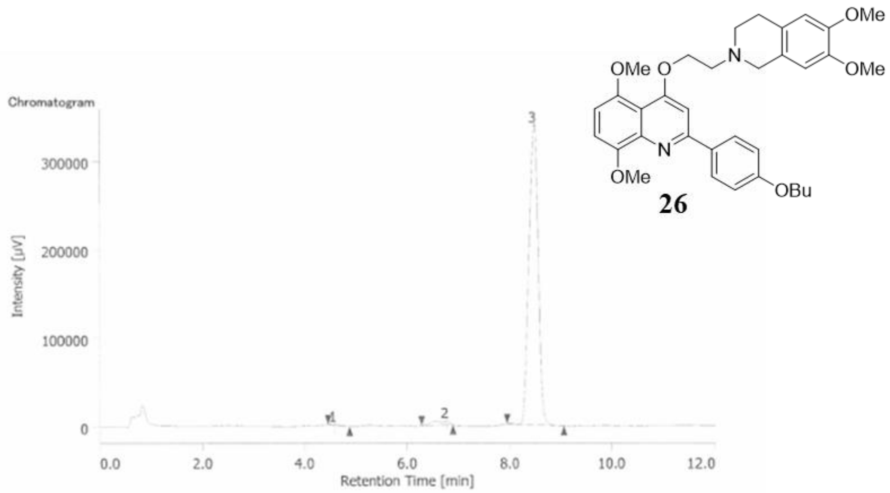

Peak Information

#	Peak Name	CH	tR [min]	Area [μV·sec]	Height [μV]	Area%	Height%	Quantity	Resolution
1	Unknown	5	4.593	13571	1408	0.308	0.406	N/A	4.511
2	Unknown	5	6.767	114793	4803	2.604	1.386	N/A	3.288
3	Unknown	5	8.470	4279688	340285	97.088	98.208	N/A	N/A

Figure S56. HPLC of compound **26**.

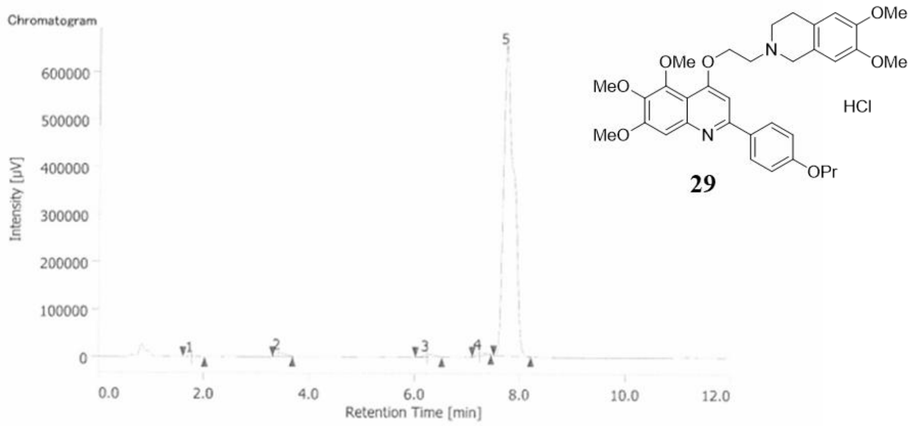

Peak Information

#	Peak Name	CH	tR [min]	Area [μV·sec]	Height [μV]	Area%	Height%	Quantity	Resolution
1	Unknown	5	1.777	30111	3381	0.328	0.497	N/A	5.287
2	Unknown	5	3.430	101322	8144	1.103	1.197	N/A	7.384
3	Unknown	5	6.240	85379	6646	0.930	0.977	N/A	3.172
4	Unknown	5	7.243	74963	7005	0.816	1.030	N/A	1.451
5	Unknown	5	7.733	8893376	655150	96.823	96.299	N/A	N/A

Figure S57. HPLC of compound **29**.

2. Biology

2.1. Cytotoxicity profiling

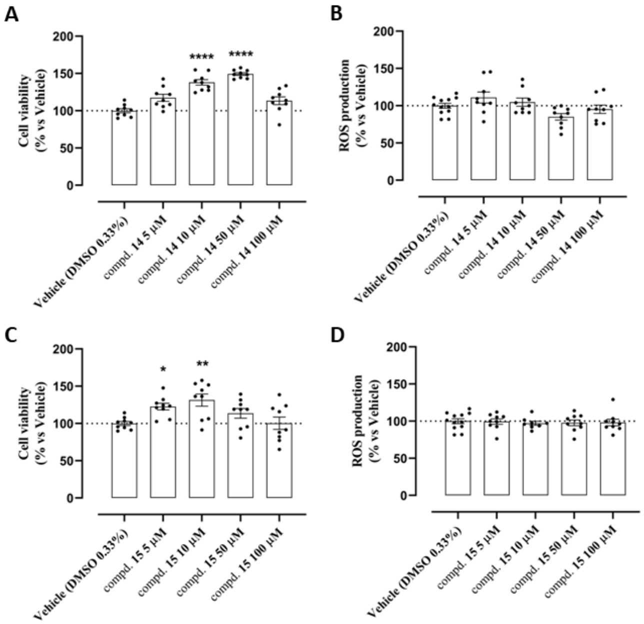

Figure S58. Evaluation of cell viability and ROS production of BEAS-2B cells after 24h incubation with vehicle (DMSO 0.33%), 14 (A, B) or 15 (C, D) (5; 10; 50; 100 μM). Data are expressed as mean ± SEM. * indicates significant statistical difference vs Vehicle (* $p<0.05$, ** $p<0.01$**** $p<0.0001$).

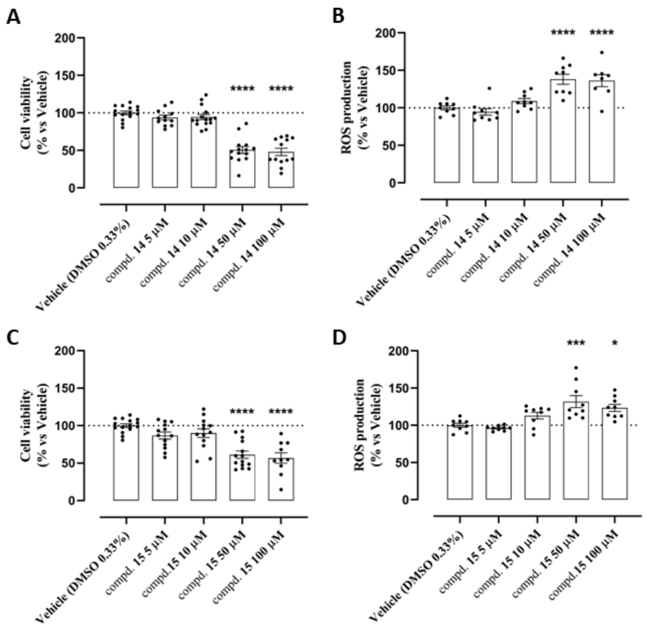

Figure S59. Evaluation of cell viability and ROS production of H9c2 cells after 24h incubation with vehicle (DMSO 0.33%) **14** (A, B) or **15** (C, D) (5; 10; 50;100 µM). Data are expressed as mean ± SEM. * indicates significant statistical difference vs Vehicle (* p<0.05, *** p<0.001; **** p<0.0001).

2.2. Preliminary In Vitro PK Evaluation of compound 15

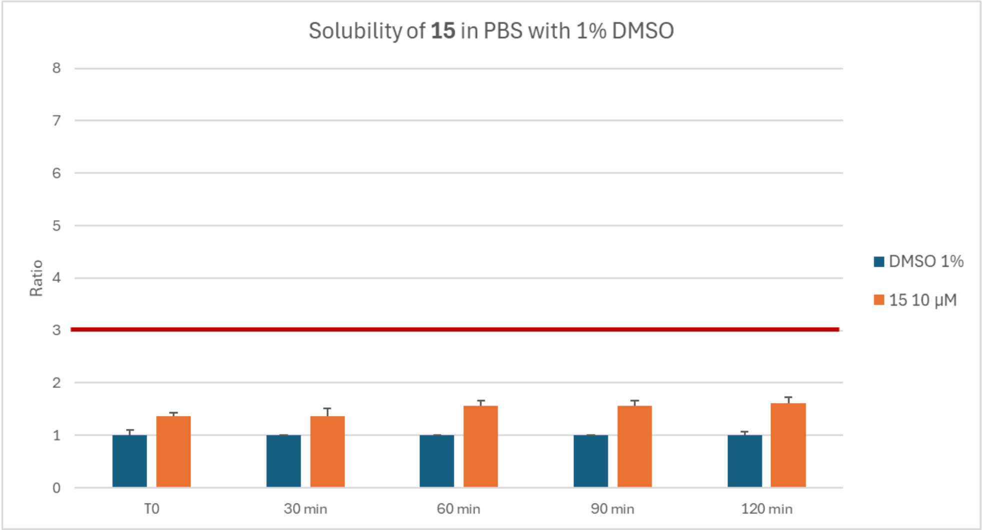

Figure S60. Solubility of **15** in PBS buffer + 1% DMSO.

Table S1. Apparent permeability value of **15**.

#	P_{app} (10^{-6} cm/s)	SD (±)
**Furosemide**	u.d.l.*	-
**Caffeine**	2.49	0.13
**Propranolol**	14.10	1.04
**15**	4.83	0.36
*u.d.l. Under detection limit		

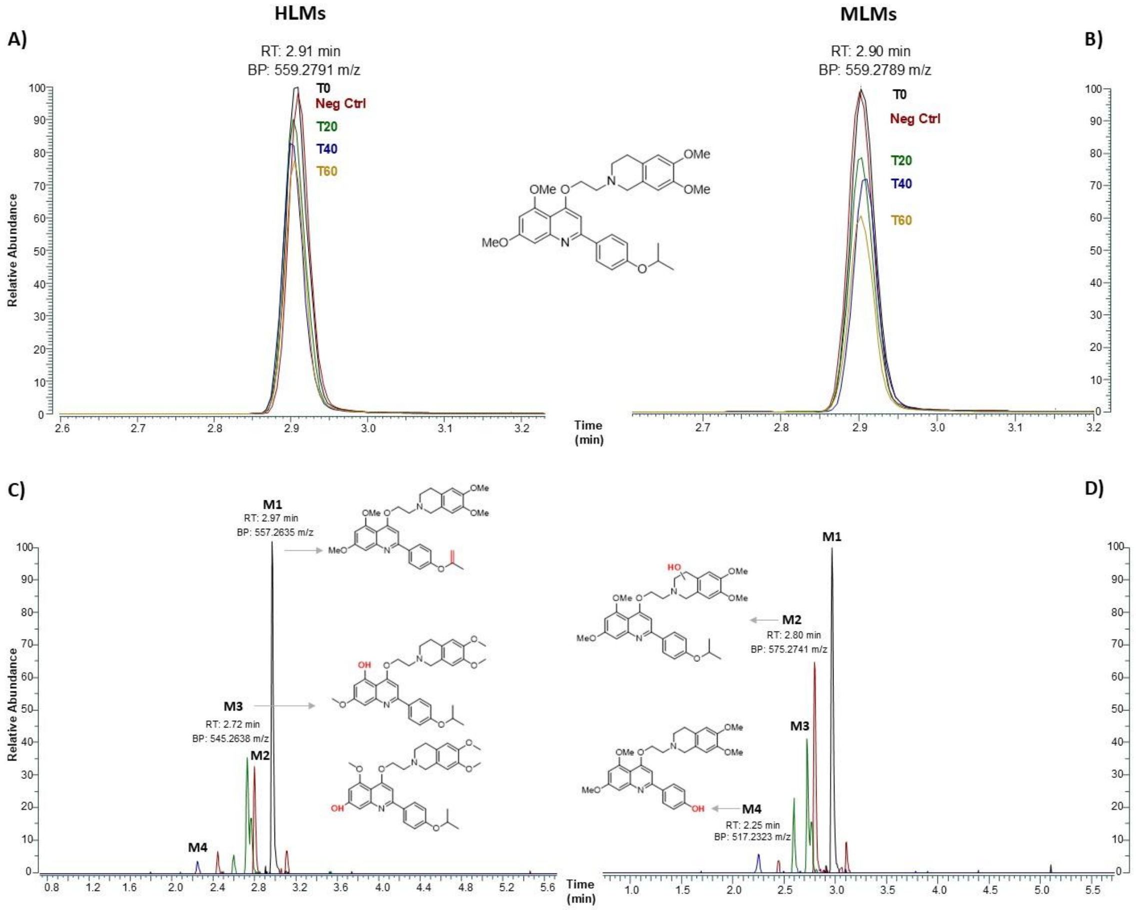

Figure S61. Time-dependent disappearance of compound **15** following incubation with human (**A**, HLMs) and mouse (**B**, MLMs) liver microsomes. Extracted ion chromatograms (XICs) of the corresponding metabolites obtained after 60 min of incubation (**C**: HLMs; **D**: MLMs).

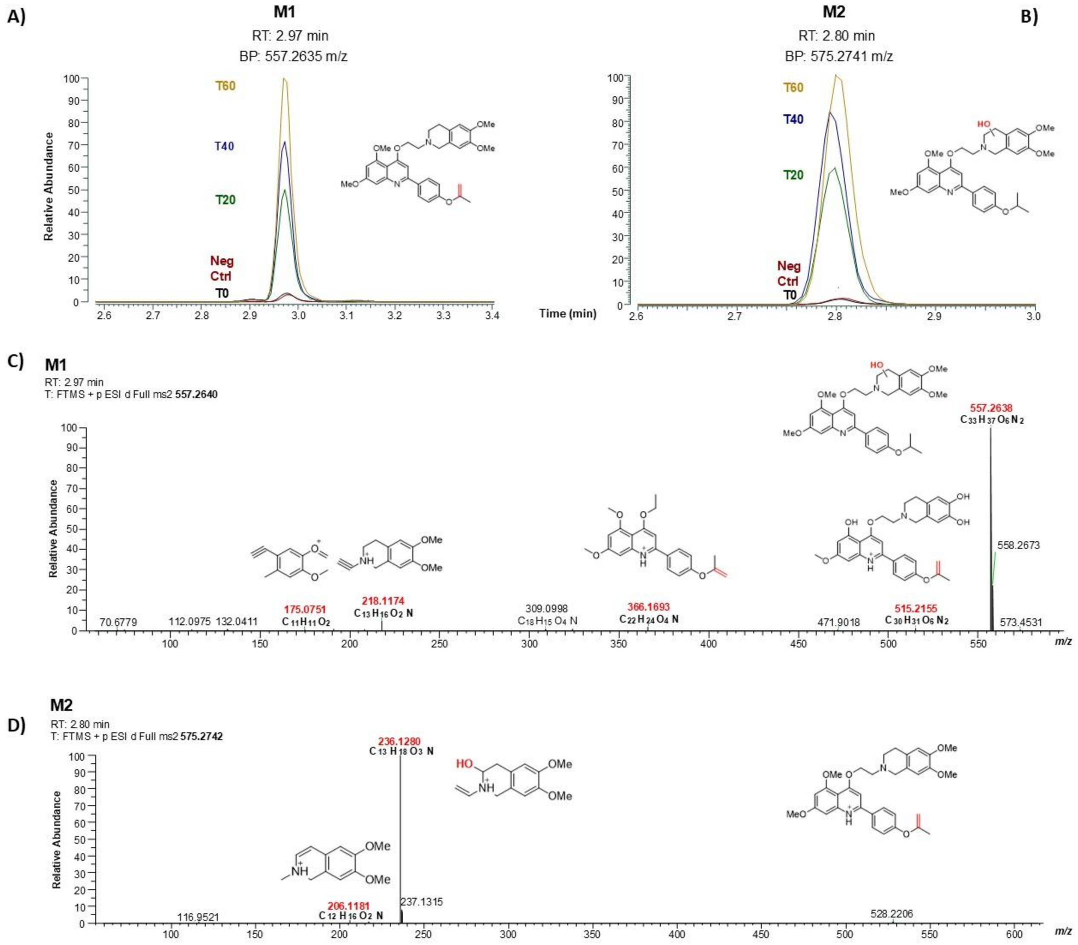

Figure S62. LC-MS profiles of the identified metabolites M1 (**A**) and M2 (**B**) at different incubation time points with liver microsomes. MS/MS spectra illustrating the fragmentation pathways of metabolites M1 (**C**) and M2 (**D**) derived from compound **15**.

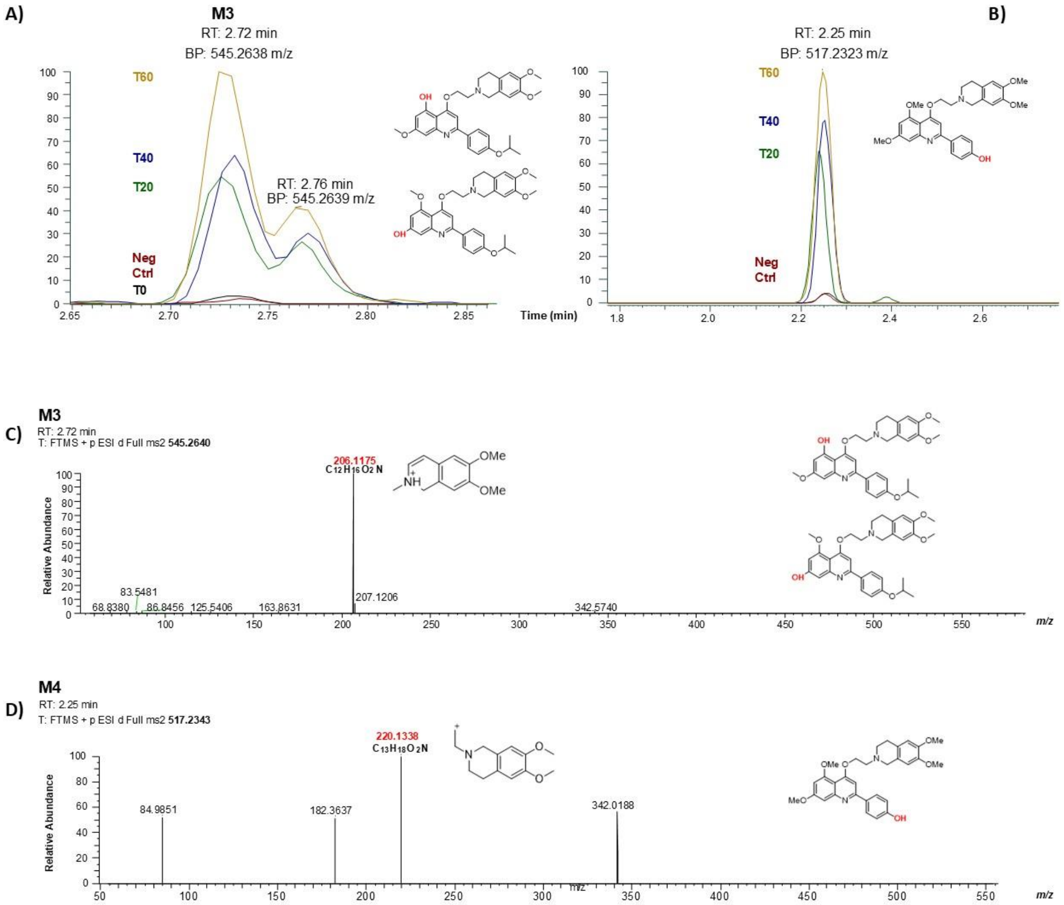

Figure S63. XICs of metabolites M3 (**A**) and M4 (**B**), and corresponding MS^2 spectra (**C**: M3; **D**: M4) following liver biotransformation of compound **15**.
